# Identification and evaluation of circulating small extracellular vesicle microRNAs as diagnostic biomarkers for patients with indeterminate pulmonary nodules

**DOI:** 10.1186/s12951-022-01366-0

**Published:** 2022-04-02

**Authors:** Di Zheng, Yuming Zhu, Jiyang Zhang, Wei Zhang, Huizhen Wang, Hao Chen, Chunyan Wu, Jian Ni, Xiaoya Xu, Baoning Nian, Sheng Chen, Beibei Wang, Xiaofang Li, Yanan Zhang, Jiatao Zhang, Wenzhao Zhong, Lei Xiong, Fugen Li, Dadong Zhang, Jianfang Xu, Gening Jiang

**Affiliations:** 1 Department of Medical Oncology, Shanghai Pulmonary Hospital, Tongji University School of Medicine, No. 507 Zhengmin Road, Yangpu District, Shanghai, 200433 China; 2 Department of Thoracic Surgery, Shanghai Pulmonary Hospital, Tongji University School of Medicine, No. 507 Zhengmin Road, Yangpu District, Shanghai, 200433 China; 3 3D Medicines Inc, Building 2A, No. 158 Xin Junhuan Road, Pujiang Hi-tech Park, Shanghai, 201114 China; 4 Department of Pathology, Shanghai Pulmonary Hospital, Tongji University School of Medicine, Shanghai, 200433 China; 5 Guangdong Lung Cancer Institute, Guangdong Provincial People’s Hospital, Guangdong Academy of Medical Sciences, Guangzhou, 510080 China

**Keywords:** Indeterminate pulmonary nodule, Low-dose computed tomography, Small extracellular vesicle, microRNA, Small RNA sequencing

## Abstract

**Background:**

The identification of indeterminate pulmonary nodules (IPNs) following a low-dose computed tomography (LDCT) is a major challenge for early diagnosis of lung cancer. The inadequate assessment of IPNs’ malignancy risk results in a large number of unnecessary surgeries or an increased risk of cancer metastases. However, limited studies on non-invasive diagnosis of IPNs have been reported.

**Methods:**

In this study, we identified and evaluated the diagnostic value of circulating small extracellular vesicle (sEV) microRNAs (miRNAs) in patients with IPNs that had been newly detected using LDCT scanning and were scheduled for surgery. Out of 459 recruited patients, 109 eligible patients with IPNs were enrolled in the training cohort (n = 47) and the test cohort (n = 62). An external cohort (n = 99) was used for validation. MiRNAs were extracted from plasma sEVs, and assessed using Small RNA sequencing. 490 lung adenocarcinoma samples and follow-up data were used to investigate the role of miRNAs in overall survival.

**Results:**

A circulating sEV miRNA (CirsEV-miR) model was constructed from five differentially expressed miRNAs (DEMs), showing 0.920 AUC in the training cohort (n = 47), and further identified in the test cohort (n = 62) and in an external validation cohort (n = 99). Among five DEMs of the CirsEV-miR model, miR-101-3p and miR-150-5p were significantly associated with better overall survival (p = 0.0001 and p = 0.0069). The CirsEV-miR scores were calculated, which significantly correlated with IPNs diameters (p < 0.05), and were able to discriminate between benign and malignant PNs (diameter ≤ 1 cm). The expression patterns of sEV miRNAs in the benign, adenocarcinoma in situ/minimally invasive adenocarcinoma, and invasive adenocarcinoma subgroups were found to gradually change with the increase in aggressiveness for the first time. Among all DEMs of the three subgroups, five miRNAs (miR-30c-5p, miR-30e-5p, miR-500a-3p, miR-125a-5p, and miR-99a-5p) were also significantly associated with overall survival of lung adenocarcinoma patients.

**Conclusions:**

Our results indicate that the CirsEV-miR model could help distinguish between benign and malignant PNs, providing insights into the feasibility of circulating sEV miRNAs in diagnostic biomarker development.

*Trial registration*: Chinese Clinical Trials: ChiCTR1800019877. Registered 05 December 2018, https://www.chictr.org.cn/showproj.aspx?proj=31346.

**Graphical Abstract:**

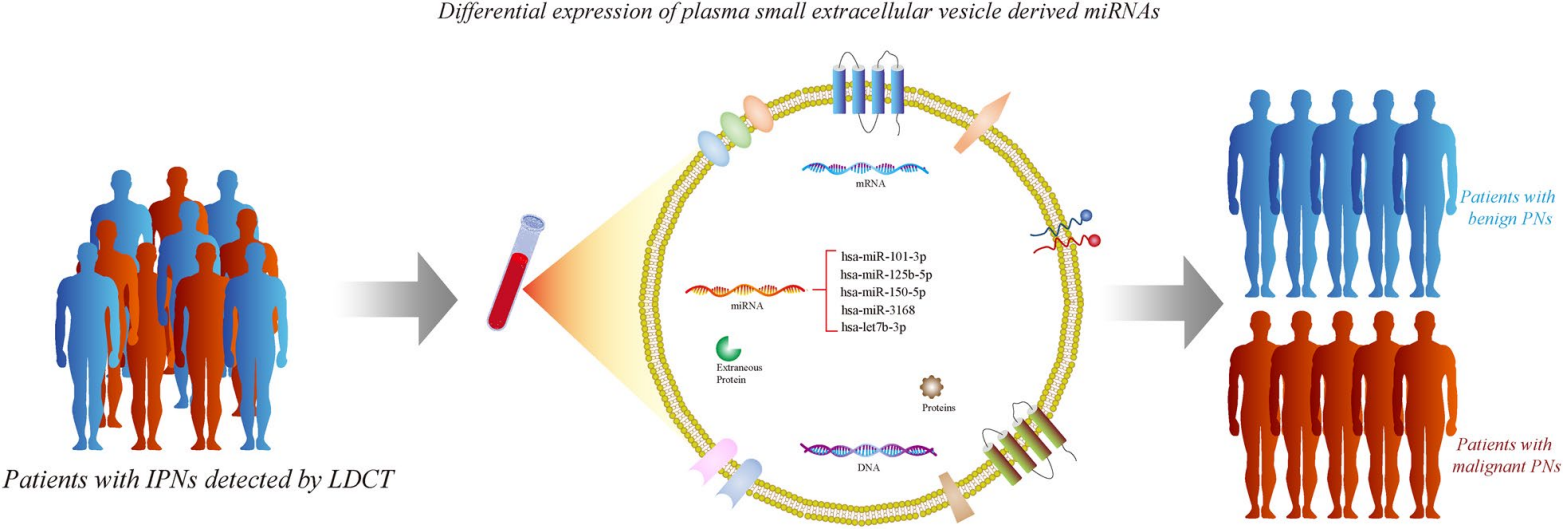

**Supplementary Information:**

The online version contains supplementary material available at 10.1186/s12951-022-01366-0.

## Background

Lung cancer is the most frequent malignancy with the highest morbidity and mortality worldwide [1]. In the U.S. National Lung Cancer Screening Test (NLST) trial [2], low-dose computed tomography (LDCT) has been proven to be a highly sensitive method to detect early stage lung cancer in patients with a smoking history. LDCT scanning has dramatically improved the ability to detect pulmonary nodules (PNs); however, a total of 96.4% of false positive screening results mandates improvements in the management of indeterminate pulmonary nodules (IPNs), which are difficult to diagnose. The inadequate assessment of IPNs’ malignancy risk is a major cause of misdiagnosis and mistreatment. Benign PNs require no surgical resection, however, the rates of benign PNs undergoing surgical resection have been reported to range from 1 to 30% in suspected lung cancer cases [3]. The invasive thoracic procedure of unnecessary operations performed in patients with benign PNs results in unnecessary costs, societal burden, and morbidity and mortality risk to the patient, with no therapeutic benefit [4, 5]. Hence, a non-invasive auxiliary diagnostic test for LDCT to improve its ability of distinguishing between benign and malignant PNs among IPNs is urgently needed.

Extensive efforts using blood biomarkers, such as DNA, RNA, and proteins to distinguish malignant from benign PNs have yielded novel insights into lung cancer diagnosis [6]. Small extracellular vesicles (sEVs), secreted by a variety of cells into the blood, contain bioactive molecules such as proteins, lipids, and nucleic acids that can mirror the cellular origin and the physiological state, and these molecules are attractive potential biomarkers representing the “fingerprint” or “signature” of the donor cell [7]. Moreover, the membranous structure of the sEVs protects the luminal contents, avoiding degradation by extracellular enzymes. The remarkable stability and activity strengthen the potential of circulating sEVs to be reservoirs for biomarker development [8, 9]. MicroRNAs (miRNAs) have been found to be the most abundant species among plasma-derived sEVs RNAs [10], and are also notably stable under different storage conditions [11]. Lung cancer cells secrete more sEVs into blood than normal tissue cells [12], and miRNAs derived from sEVs of lung cancer patients have also been found to be significantly different from those of healthy people, indicating that serum/plasma sEV miRNAs as potential biomarkers of lung cancer [13, 14]. Some studies have attempted to distinguish malignant PNs using plasma sEV miRNAs [15–17], however, these studies included healthy people as controls to develop a diagnostic model, and lacks further investigation in biological difference between benign and malignant PNs. Zhang and colleagues managed to distinguish malignant ground-glass nodules and benign nodules using plasma sEV miRNAs, but the sample sizes were relatively small [18]. The discrimination between benign and malignant PNs are quite difficult to identify, yet very important for accurate diagnosis of lung cancer after LDCT scanning in clinical practice.

In the present study, we assessed the expression levels of circulating sEV miRNAs using small RNA sequencing to detect the differences between patients with benign PNs and patients with malignant PNs (early- to mid-stage lung adenocarcinoma). We then developed a CirsEV-miR model to differentiate between benign and malignant PNs in a training cohort (n = 47) and further confirmed the model in a test cohort (n = 62) and in an external validation cohort (n = 99). Furthermore, we calculated CirsEV-miR scores and for the first time explored the relationship between CirsEV-miR scores and clinical characteristics, including the diameter of IPNs and the PN aggressiveness in the benign, adenocarcinoma in situ (AIS)/minimally invasive adenocarcinoma (MIA), and invasive adenocarcinoma. We also found that the circulating sEV miRNA signature was able to discriminate between benign and malignant PNs with the diameter ≤ 1 cm, which are otherwise difficult to distinguish in clinical practice. In total, we analyzed circulating sEV miRNAs of 208 patients with IPNs, providing the largest sample size among the available studies. Additionally, 490 lung adenocarcinoma samples and follow-up data from The Cancer Genome Atlas (TCGA) were used to investigate the role of miRNAs in overall survival. The development of such non-invasive diagnostic test, *i.e.*, the CirsEV-miR model, may complement the highly sensitive but insufficiently specific LDCT and be integrated into the diagnostic algorithm to achieve higher diagnostic accuracy for patients with IPNs.

## Results

### Participants and clinical characteristics

**Fig. 1:**
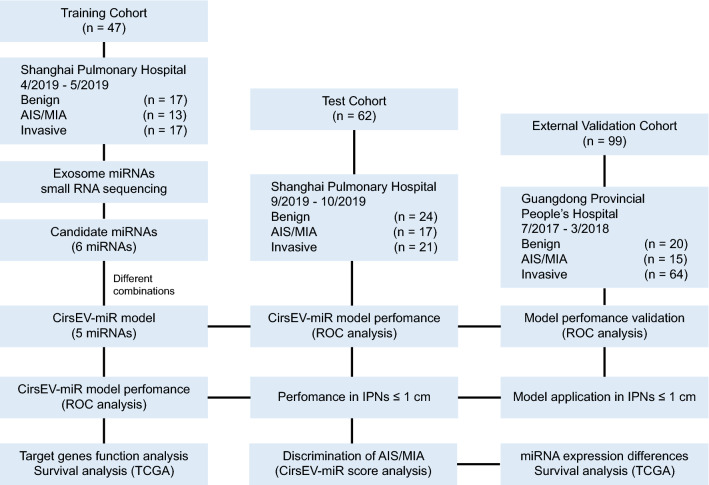
Overall study design and patients in the training, test, and external validation cohorts. *AIS* adenocarcinoma in situ; *MIA* minimally invasive carcinoma, *ROC* receiver operating characteristic

The workflow of our study is illustrated in Fig. 1 To explore circulating sEV miRNAs as diagnostic biomarkers in patients with lung IPNs, a total of 199 and 260 patients were recruited in the training phase and the test phase, respectively. After the elimination of hemolysis, non-lung adenocarcinoma (non-LUAD), failure of sequencing library construction, and limitation of the ratio of benign and malignant PNs, a total of 109 patients were included in two independent cohorts (Additional file 1: Figure S1). The training cohort consisted of 47 patients with IPNs, including 17 benign PNs (benign group) and 30 malignant PNs (malignant group) identified using pathological diagnosis; the test cohort consisted of 62 patients with IPNs, including 24 benign PNs and 38 malignant PNs. Benign PNs were used as controls. In addition, an external validation cohort (n = 99), including 20 patients with benign PNs and 79 patients with malignant PNs, was used to validate the model (Fig. 1 and Table 1), and 11 healthy people were as healthy control. More than 90% of LUAD patients (92.6%, 63/68) were in the early stage (no lymph node metastasis), and 33.0% (36/109) of patients had IPNs with a diameter less than 1 cm (Table 1). The demographic and clinicopathologic characteristics of the patients are shown in Table 1. Benign PNs included cases of atypical adenomatous hyperplasia (AAH), fibrosis, granulomas, hamartoma, organizing pneumonias (OPs), cyst, and other benign subtypes, while malignant PNs included AIS, MIA, and invasive adenocarcinoma. The pathology subtype composition of the three cohorts is shown in (Additional file 1: Figure S2). The representative imaging features of benign and malignant PNs are shown in (Additional file 1: Figure S3).

**Table 1:** The demographic and clinicopathologic characteristics of the patients in three cohorts

Characteristic	Training cohort (n = 47)	Testing cohort (n = 62)	External cohort (n = 99)
Categories—no. (%)			
Benign	17 (36.2)	24 (38.7)	20 (20.2)
Malignant	30 (63.8)	38 (61.3)	79 (79.8)
Age, mean (SD)	58.7 (12.5)	58.1 (10.7)	57.9 (11.9)
Age, no. (%)			
< 60	24 (51.1)	28 (45.2)	49 (49.5)
≥ 60	23 (48.9)	34 (54.8)	50 (50.5)
Gender, no. (%)			
Female	20 (42.6)	37 (59.7)	55 (55.6)
Male	27 (57.4)	25 (40.3)	44 (44.4)
Smoking status, no. (%)			NA
Yes	12 (25.5)	6 (9.7)	
No	35 (74.5)	56 (90.3)	
Pathology, no. (%)			
AIS	3 (6.4)	6 (9.7)	10
MIA	10 (21.3)	11 (17.7)	5
Invasive	17 (36.2)	21 (33.9)	66
AAH	2 (4.3)	2 (3.2)	0
Fibrosis	2 (4.3)	4 (6.5)	0
Granulomas	4 (8.5)	3 (4.8)	0
Hamartoma	2 (4.3)	3 (4.8)	3
OP	4 (8.5)	4 (6.5)	0
Other benign subtypes	3 (6.4)	8 (12.9)	15
Nodule diameter (cm), no. (%)			
≤ 1	12 (25.5)	24 (38.7)	21 (21.2)
> 1	35 (74.5)	38 (61.3)	78 (78.8)
Malignant stages, no. (%)			NA
0	3 (10.0)	6 (15.8)	
IA1	12 (40.0)	13 (34.2)	
IA2	5 (16.7)	11 (28.9)	
IA3	6 (20.0)	4 (10.5)	
IB	1 (3.3)	0	
IIA	2 (6.7)	0	
IIB	0	1 (2.6)	
IIIA	1 (3.3)	3 (7.9)	

*AIS* adenocarcinoma in situ, *MIA* minimally invasive adenocarcinoma, *AAH* atypical adenomatous hyperplasia, *OP* organizing pneumonias, *NA* not available

### Circulating sEV characterization

**Fig. 2:**
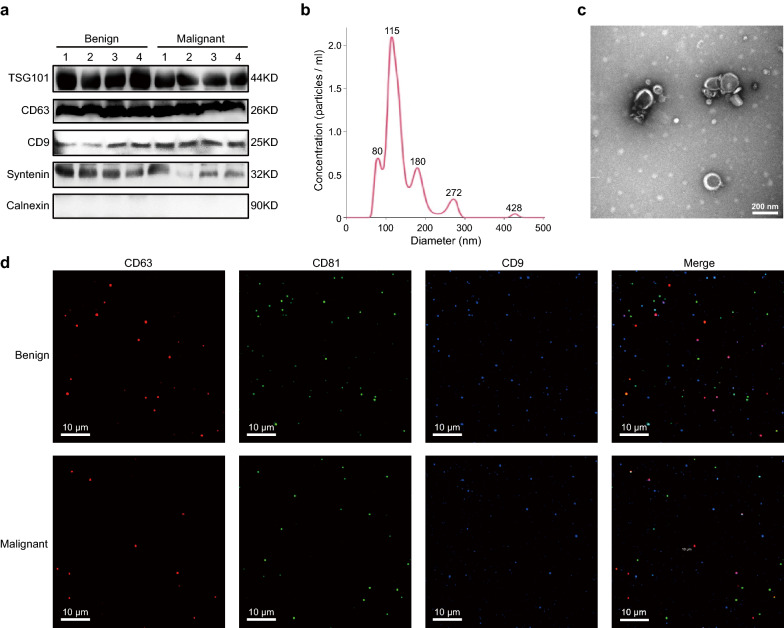
Characterization of circulating small extracellular vesicles (sEVs). **a** Western blotting identified sEV proteins of eight representative samples, including the sEV positive markers TSG101, CD63, CD9, Systenin, and the negative marker Calnexin. **b** Nanoparticle tracking analysis (NTA) results from representative sEV samples. **c** Transmission electron microscopic (TEM) images of representative sEVs. **d** Representative spots and immunofluorescence staining of CD63 (red), CD81 (green), and CD9 (blue)

Circulating sEVs were successfully isolated from patient plasma samples and characterized using western blot (WB) analysis, nanoparticle tracking analysis (NTA), and transmission electron microscopy (TEM). In accordance with the Minimal Information for Studies of Extracellular Vesicles (MISEV) 2018 [19], several protein markers were evaluated using WB in eight representative sEV samples from the patients with benign and malignant PNs. The expression levels of TSG101, CD63, CD9, and Syntenin were detected in the eight sEV samples, while a negative marker, Calnexin, was absent in all eight sEV samples (Fig. 2a). Furthermore, the majority of the isolated sEVs were around 100 nm in diameter, which is the typical size of sEVs (Fig. 2b). The TEM result from a representative sample showed that the isolated sEVs were cup-shaped (Fig. 2c), which is the typical morphology of sEVs. Furthermore, we also detected sEV transmembrane proteins (CD63, CD81, CD9) using ExoView platform. As shown in Fig. 2d, CD63, CD81, and CD9 were all detected in plasma sEVs of patients with benign or malignant PNs.

### Construction and validation of a circulating sEV miRNA (CirsEV-miR) model for IPN diagnosis

**Fig. 3:**
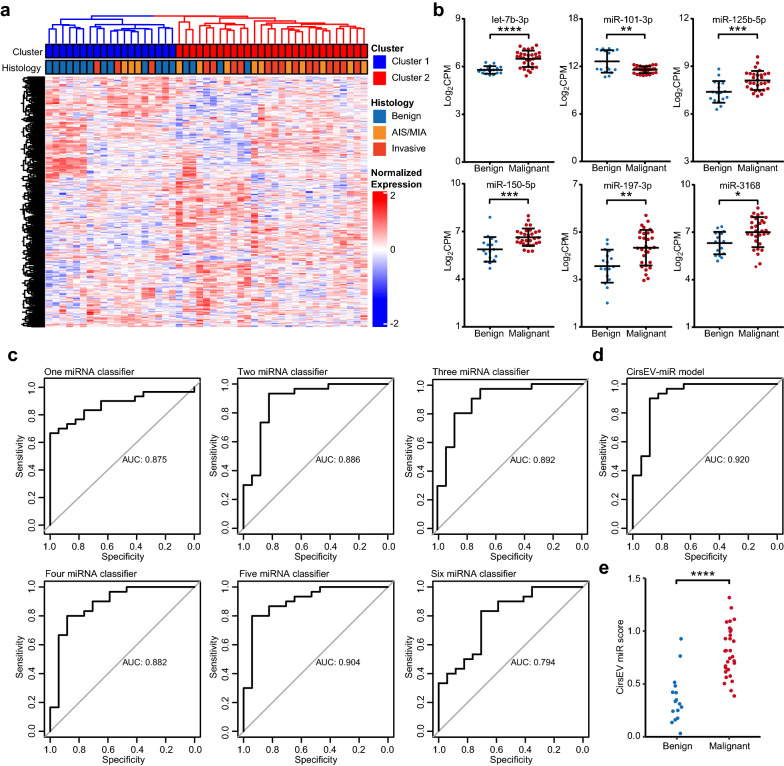
Classifier development process for IPNs. **a** Heatmap of sEV-miRNA expression in the training cohort by unsupervised hierarchical clustering. **b** Expression levels of six identified DEMs in the training cohort. **c** ROCs of classifiers constructed with different combinations of the six DEMs. Only ROCs with the best diagnostic powers of different miRNA combinations are shown. **d** ROC curve of the CirsEV-miR model in the training cohort. **e** CirsEV-miR scores of benign and malignant PNs in the training cohort

We then extracted the RNA from sEVs and performed small RNA sequencing. Raw data of small RNA sequencing were filtered and normalized. Unsupervised hierarchical clustering based on the expression levels of circulating sEV miRNAs categorized the samples in a similar pattern as the clinicopathologic classifications did, both in the training cohort (Fig. 3a) and in the test cohort (Additional file 1: Figure S4a), thereby suggesting the feasibility of predicting clinicopathologic classifications using circulating sEV miRNA expression. Six DEMs between benign and malignant PNs were identified in the training cohort (Fig. 3b), and these DEMs were selected to construct a model for distinguishing benign PNs from malignant PNs. To verify the potential of miRNAs as a suitable classifier, receiver operating characteristic (ROC) analysis was performed, and the area under curve (AUC) was calculated (Fig. 3c, Additional file 1: Table S1, Additional file 1: Table S2). Let-7b-3p exhibited an AUC of 0.875, which was the best performance of an individual miRNA. Next, we integrated two or more miRNAs to further improve the performance. Integration of let-7b-3p and miR-125b-5p led to a slightly improved AUC of 0.886; integrating three miRNAs achieved an AUC of 0.892 (let-7b-3p, miR-125b-5p, and miR-197-3p); and integrating four miRNAs achieved an AUC of 0.882 (let-7b-3p, miR-125b-5p, miR-197-3p, and miR-150-5p). Integration of five miRNAs achieved an even higher AUC of 0.904 (let-7b-3p, miR-125b-5p, miR-197-3p, miR-150-5p, and miR-3168), while the AUC of the integration of all six miRNAs dropped to 0.794. We then used LASSO-penalized regression to develop a classifier of five miRNAs (let-7b-3p, miR-125b-5p, miR-150-5p, miR-101-3p, and miR-3168), called the CirsEV-miR model, which exhibited an AUC of 0.920 in the training cohort (Fig. 3d, Additional file 1: Table S2, Additional file 1: Table S3), which was the highest AUC among all classifiers. The sensitivity reached 0.900, and the specificity was 0.882. Based on DEMs, CirsEV-miR scores were also generated through LASSO analysis. CirsEV-miR scores of malignant PNs were significantly higher than those of benign PNs in the training cohort (Fig. 3e, p < 0.0001), suggesting that CirsEV-miR scores increase with malignancy. The CirsEV-miR model was further confirmed in the test cohort, showing an AUC of 0.763 (Additional file 1: Figure S4b) and significant differences in CirsEV-miR scores between benign and malignant IPNs (Additional file 1: Figure S4c, p = 0.0004). The CirsEV-miR model was also validated in the external cohort consisting of 20 patients with benign PNs and 79 patients with malignant PNs; the AUC in that cohort was 0.781 (Additional file 1: Figure S4d). We were also interested in finding the difference in the circulating sEV miRNAs between healthy participants and lung cancer and benign pulmonary nodule patients. Therefore, to examine the discrimination value of CirsEV-miR score, the blood specimens from 11 healthy participants were collected and used to analyze the expression levels of the circulating sEV miRNAs using small RNA sequencing. The results showed that healthy controls could be significantly different from the lung cancer (Additional file 1: Figure S4e, p = 3.07E-06) and benign pulmonary nodule patients (Additional file 1: Figure S4e, p = 0.015), indicating that the CirsEV-miR score has great capability of discriminating IPNs compared to healthy people. Expression of the five miRNAs of the CirsEV-miR model was also verified by quantitative reverse-transcription PCR in 26 patients with benign or malignant PNs, showing similar patterns with sequencing results (Additional file 1: Figure S4f). These results revealed that the CirsEV-miR model may be a new promising approach to assist in the differential diagnosis of lung IPNs.

### Biological function enrichment and overall survival analysis of five DEMs in the CirsEV-miR model

**Fig. 4:**
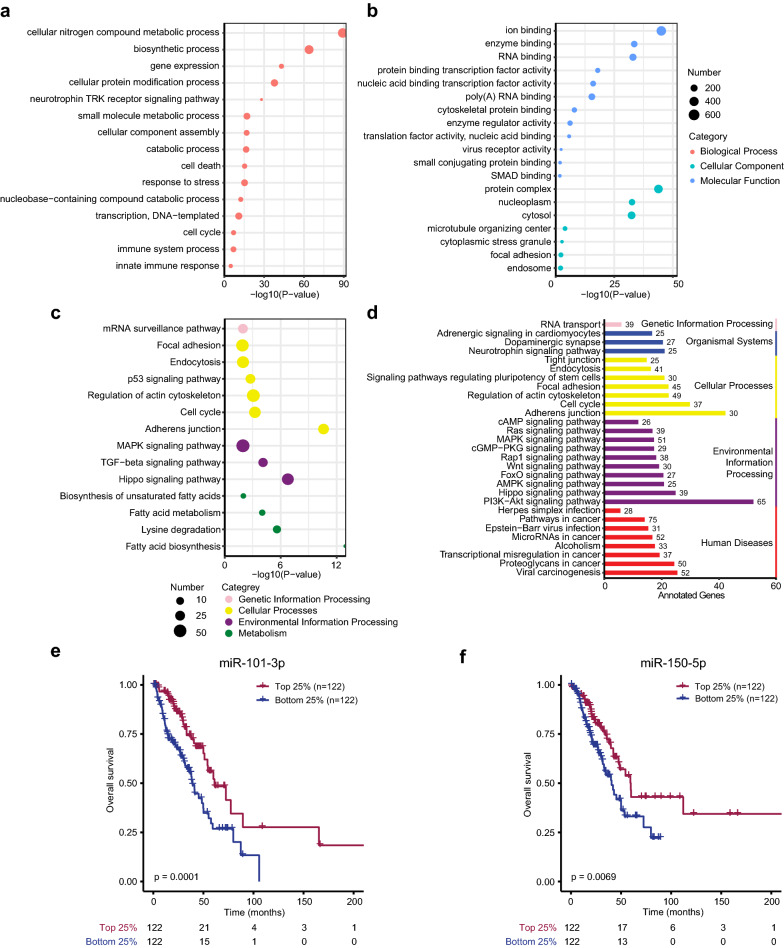
Target analysis and survival analysis of the five DEMs used for CirsEV-miR model development. A bubble plot of enriched GOs ((Biological process, (**a**), and (Cellular component and Molecular Function, (**b**)) of target genes of the five DEMs. **c** A bubble plot of enriched KEGG pathways of target genes of the five DEMs. **d** A bar plot showing the number of DEM target genes in each of the KEGG pathways (target genes > 20). **e, f** Overall survival analysis of miR-101-3p **(e)** and miR-150-5p **(f)** from the TCGA database

To evaluate the potential functions of the five miRNAs used in the CirsEV-miR model, we performed Gene Ontology (GO) and pathway analysis of their target genes (Additional file 1: Table S4). We found that 2238 mRNAs were targeted by the five miRNAs through the analysis of the miRNA target database. The target genes were enriched in biological processes (BPs), such as cellular nitrogen compound metabolic process, biosynthetic process, and cellular protein modification processes (Fig. 4a), and in molecular functions (MFs), such as ion binding, enzyme binding, and RNA binding (Fig. 4b). In addition, the target genes were significantly enriched in pathways related to tumorigenesis and progression processes, such as MAPK, TGFβ, Hippo, p53 signaling pathways, cell cycle, and adherence junction processes (Fig. 4c, d). Moreover, 490 lung adenocarcinoma samples and their follow-up data from the TCGA showed that patients with high expression of miR-101-3p or miR-150-5p in tumor tissue samples had better overall survival (Fig. 4e, f, p = 0.0001; p = 0.0069). However, the other three miRNAs, let-7b-3p, miR-125b-5p, and miR-3168, were not significantly associated with overall survival (data not shown). The functional enrichment analysis indicated that five miRNAs were involved in tumorigenesis and progression of lung cancer.

### The signature of circulating sEV miRNAs discriminated between benign and malignant PNs with diameter ≤ 1 cm.

**Fig. 5:**
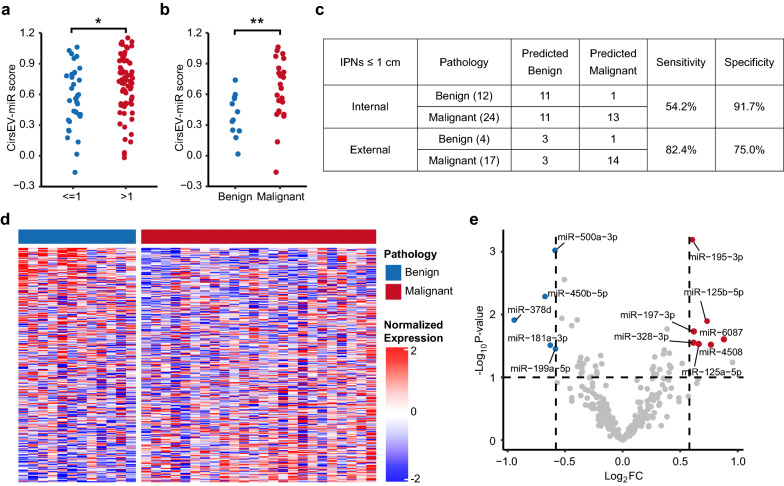
The CirsEV-miR scores increased with the increase in the diameter of IPNs. **a** CirsEV-miR scores of IPNs ≤ 1 cm and > 1 cm. **(b)** The CirsEV-miR scores of benign and malignant subgroups of IPNs ≤ 1 cm. **c** Model sensitivity and specificity of IPNs ≤ 1 cm. **d** Gene expression heatmap of benign and malignant subgroups of IPNs ≤ 1 cm. **e** Differentially expressed miRNAs between the benign and malignant subgroups of IPNs ≤ 1 cm

The size of IPNs is a key factor that is associated with their malignant potential and patients’ long term survival [20, 21]. In clinical practice, IPNs with a diameter > 1 cm are thought to need resection, whereas IPNs with a diameter ≤ 1 cm require repeated scans and a long-term follow-up [22]. Thus, we also analyzed the correlation between the nodule size and risk score of the CirsEV-miR model. All 109 eligible samples were recategorized into two subgroups: the IPNs with diameter ≤ 1 cm and IPNs with diameter > 1 cm, and re-ranked according to the CirsEV-miR scores. The CirsEV-miR scores were significantly higher in the subgroup of IPNs with diameter > 1 cm (Fig. 5a, p = 0.0170), suggesting that the CirsEV-miR scores remarkably increase with the diameter of IPNs. Moreover, among the patients with small IPNs (diameter ≤ 1 cm), malignant PNs also exhibited higher CirsEV-miR scores than benign PNs (Fig. 5b, p = 0.0088). The AUC of the CirsEV-miR model in patients with small IPNs (diameter ≤ 1 cm) was 0.767 in our cohort (Additional file 1: Figure S5a), and 0.721 in the external validation cohort (Additional file 1: Figure S5b). Moreover, we achieved the diagnostic specificity of 91.7% for the IPNs with diameter ≤ 1 cm in our cohort, and specificity of 75% in the external validation cohort (Fig. 5c). Among the IPNs with diameter ≤ 1 cm, benign and malignant subgroups showed different miRNA expression patterns (Fig. 5d). Twelve DEMs between benign and malignant PNs with diameter ≤ 1 cm were identified (Fig. 5e), and eight of them were involved in lung cancer tumorigenesis and progression, suggesting that even small IPNs were different from benign PNs. These results suggest that even in IPNs with diameter ≤ 1 cm, the circulating sEV miRNA signature can separate benign PNs from malignant PNs.

### Circulating sEV miRNA analysis reveals that AIS/MIA can alleviate the differentiation between benign and malignant PNs

**Fig. 6:**
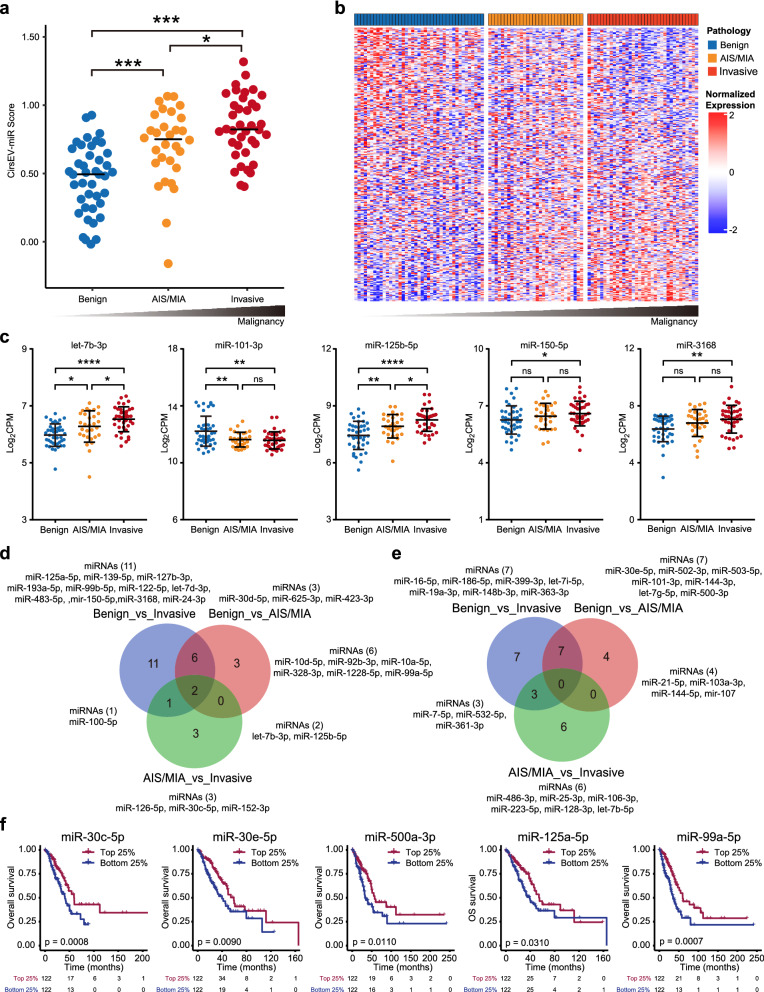
AIS/MIA are intermediate between benign and malignant PNs. **a** The CirsEV-miR scores of the benign, AIS/MIA, and invasive adenocarcinoma subgroups categorized according to the pathologies. **b** Gene expression heatmap of all genes in the benign, AIS/MIA and invasive adenocarcinoma subgroups. **c** The expression levels of the five DEMs in the benign, AIS/MIA, and invasive adenocarcinoma subgroups. **d**, **e** Venn diagrams of upregulated (**d**) or downregulated (**e**) genes of the benign, AIS/MIA, and IA subgroups. **f** Overall survival analysis of miR-30c-5p, miR-30e-5p, miR-500a-3p, miR-125a-5p, and miR-99a-5p from the TCGA database. ***, p < 0.001; **, p < 0.01; *, p < 0.05; ns, not significant

AIS and MIA represent early-stage lung cancer, and both AIS and MIA patients possess superior prognoses to invasive adenocarcinoma [23, 24]. Therefore, we speculated that the possible reason for the relatively low sensitivity of the current model was the misprediction of the clinicopathologically equivocal AIS/MIA samples. To test this hypothesis, 109 eligible samples were categorized into three subgroups: benign (41, 37.6%), AIS/MIA (30, 27.5%), and invasive adenocarcinoma (38, 34.9%). The proportions of AIS/MIA samples in the training and the test cohorts were 27.7% and 27.4%, respectively (Table 1). For all 109 samples, we calculated the CirsEV-miR scores and ranked each subgroup. The CirsEV-miR scores in the benign, AIS/MIA and invasive adenocarcinoma subgroups gradually changed with the increase in aggressiveness (Fig. 6a). We also tested the miRNA expression levels; we found that the expression pattern of the AIS/MIA subgroup was intermediate between benign and invasive adenocarcinoma subgroups (Fig. 6b). The expression levels of the five DEMs in the AIS/MIA subgroup were located between the benign and the invasive adenocarcinoma subgroups (Fig. 6c). We further compared all DEMs between the benign, AIS/MIA, and invasive adenocarcinoma subgroups (Fig. 6d, e, Additional file 1: Table S5, Additional file 1: Table S6). We only found two overlapping upregulated DEMs (let-7b-3p and miR-125b-5p), which were also elements of our CirsEV-miR model. Thirteen miRNAs (six upregulated and seven downregulated) with repeated emergence were found in Benign_vs_AIS/MIA and Benign_vs_Invasive adenocarcinoma subgroups, showing some similarity in sEV miRNA profiles of AIS/MIA and invasive adenocarcinoma. Meanwhile, three upregulated miRNAs and four downregulated miRNAs were found in Benign_vs_AIS/MIA alone, while nine miRNAs showed altered expression in invasive adenocarcinoma compared with AIS/MIA (three upregulated and six downregulated), suggesting difference in sEV-miRNA profiles between AIS/MIA and invasive adenocarcinoma. Among all DEMs, five miRNAs (miR-30c-5p, miR-30e-5p, miR-500a-3p, miR-125a-5p, and miR-99a-5p) were significantly associated with overall survival from the TCGA database (Fig. 6f, p = 0.0008; p = 0.0090; p = 0.0110; p = 0.0310, p = 0.0007). Taken together, the results of the circulating sEV miRNA analysis demonstrated that the presence of AIS/MIA could affect the differentiation between benign and malignant PNs, and that AIS/MIA presented the intermediate molecular features of circulating sEV miRNAs between benign and malignant PNs.

## Discussion

With the development of LDCT scanning for early screening and early diagnosis of lung cancer, the number of detected IPNs has dramatically increased. The inadequate assessment of IPNs’ malignancy risk is a major cause of a large number of unnecessary surgeries or an increased risk of cancer metastases; hence, the accurate diagnosis of IPNs becomes one of the major challenges in clinic. However, a non-invasive diagnostic test to assist LDCT to distinguish benign PNs from malignant PNs in IPN diagnosis is not yet available. In this study, we included 208 patients with IPNs, which is the largest sample size among the available studies; and we developed, tested, and validated the CirsEV-miR model to discriminate between benign and malignant PNs using small RNA sequencing of circulating sEV miRNAs. We also calculated a CirsEV-miR score from the model, and we found that this score remarkably increased with the diameter of IPNs, and then gradually changed with the increase in aggressiveness. Moreover, we found that the molecular features of circulating sEV miRNAs of AIS/MIA were between benign and invasive adenocarcinomas, meanwhile, AIS/MIA also possessed unique circulating sEV miRNA profiles different from the other two subgroups. Furthermore, 490 lung cancer samples and follow-up data from TCGA were used to investigate the role of miRNAs in overall survival. In summary, the CirsEV-miR model developed using circulating sEV miRNAs could serve as a non-invasive auxiliary test to help physicians to manage the IPNs for early-stage lung cancer diagnosis. The differentially expressed circulating sEV miRNAs identified in this study might further serve as potential therapeutic targets in future investigations.

This study focused on IPNs and enrolled the patients with highly suspicious malignant PNs undergoing surgery (404/459, confirmed by pathology diagnosis after surgery) from the routine work-ups in a tertiary hospital. Unlike previous studies that focused on early lung cancer screening in low-prevalence populations, our study recruited patients with a high prevalence, and we used benign PNs as controls. The discrimination between malignant and benign PNs was more difficult than that between the nodule population and non-nodule controls [25]. Benign PNs in this study contained a variety of pathological subtypes without selection, reflecting real-world clinical situations. The benign PNs in the external validation cohort contained different pathological subtypes from those in the training and test cohorts, yet yielding no significant impairment in the model performance, which indicates that our CirsEV-miR model may have broad clinical application potential.

The diameter is an important parameter in assessing the malignant potential of an IPN. The possibility of malignancy positively correlates with the diameter. Lung cancer probability was low in IPNs smaller than 5 mm, intermediate in IPNs with a diameter of 5–10 mm, and high in IPNs larger than 10 mm [22]. Clinically, IPNs > 10 mm would be considered to need resection, while IPNs ≤ 10 mm would require repeated scans. We found that CirsEV-miR score was significantly higher in IPNs > 10 mm than in IPNs ≤ 10 mm. Even in IPNs ≤ 10 mm, CirsEV-miR score of malignant PNs was significantly higher than that of benign PNs (Fig. 5a, b), suggesting that malignancy may increase with IPNs diameter. Our CirsEV-miR model also had good performance in IPNs with a diameter of less than 1 cm, exhibiting AUC of 0.767 and specificity of 91.7% in our cohorts, and AUC of 0.721 and specificity of 75.0% in the external validation cohort (Fig. 5c, Additional file 1: Figure S5). Taken together, these findings indicate that our model has the ability to distinguish between benign and malignant PNs, even in IPNs ≤ 10 mm, thereby expanding the applications widely.

According to IASLC/ATS/ERS and 2015 WHO classification of lung tumors, AIS is a tumor smaller than 3 cm with no invasion, and MIA denotes tumor smaller than 3 cm with invasion less than 0.5 cm [26]. Both AIS and MIA patients achieve 100% DFS with complete resection [27]. It is believed that LUAD develops stepwise from benign AAH to AIS, to MIA, and eventually to overt invasive adenocarcinoma [28]. However, most studies have focused on histopathological subtypes, while little is known about the genomic evolution from preinvasive to invasive adenocarcinoma of LUAD. In 2019, Zhang et al. reported that mutations in *EGFR*, *ERBB2*, *NRAS*, and *BRAF*, as well as genomic intratumor heterogeneity and immunoediting, are common and early phenomena harbored by AIS, MIA, and IA, and that mutations in *TP53*, as well as cell mobility and metastasis-related genes, may be later events during neoplastic progression [29]. Our results also demonstrated that the expression profiles of circulating sEV miRNAs in patients with AIS/MIA were intermediate between the patients with benign PNs and the patients with invasive adenocarcinoma (Fig. 6b), supporting the stepwise development hypothesis. Yet, we found seven expression-altered miRNAs unique to AIS/MIA compared with benign PNs, and nine miRNAs unique to AIS/MIA compared with invasive adenocarcinoma (Fig. 6d, e, Additional file 1: Table S5, Additional file 1: Table S6). Among them, miR-30e-5p, a well-known tumor suppressor that suppresses tumorigenesis via the Sirt1/JAK/STAT3 signaling pathway [30], was downregulated in both AIS/MIA and invasive adenocarcinoma compared with benign PNs. However, miR-25-3p and miR-128-3p, which have been reported to promote metastasis, exhibited higher expression in AIS/MIA than in invasive adenocarcinoma [31, 32]. These findings suggest that AIS/MIA exhibit unique features different from benign PNs or invasive adenocarcinoma. It is also proposed that due to a lack of signals in AIS/MIA specimens, the difficulty in distinguishing benign PNs from AIS/MIA IPNs is an intrinsic feature [33]. This study is the first report that revealed the circulating sEV miRNA signatures of benign, AIS/MIA, and invasive adenocarcinoma, suggesting that the presence of AIS/MIA could impact the differentiation between benign and malignant PNs.

miRNAs bind to the 3ʹ-untraslated region (UTR) of target mRNA, resulting in post-translational gene silencing either by mRNA degeneration or by inhibition of translation. Thousands of miRNAs have been linked to various human diseases, including cancers [34]. In this study, we identified differentially expressed miRNAs between benign and malignant PNs and constructed the diagnostic model composed of five DEMs using LASSO-penalized regression. Functional enrichment analysis revealed that the target genes of the five miRNAs of our diagnostic model were involved in many cancer-related pathways, such as MAPK, TGFβ, Hippo, p53 signaling pathway, focal adhesion, and cell cycle (Fig. 4c, d). We also found sEV-miRNAs enriched in immune system processes and metabolic processes, in accordance with the previous report that intratumor heterogeneity and immunoediting are early phenomena in PNs [29]. Moreover, we found seven miRNAs (miR-101-3p, miR-150-5p, miR-30c-5p, miR-30e-5p, miR-500a-3p, miR-125a-5p, and miR-99a-5p) that were associated with better overall survival based on the TCGA databank (Figs. 4e, f and 6f). Interestingly, these miRNAs have all been reported to function as tumor suppressors [35–40], although some of them were upregulated in tumor tissues [37, 39], and in circulating sEVs of patients with malignant PNs in our study, indicating that further research is required to clarify the molecular mechanism underlying benign and malignant PNs.

Several limitations of this study are worth mentioning. First, this was a single-center case control study that was performed in a high prevalence population. Thus, the performance of the CirsEV-miR model needs to be explored further in a community where a low prevalence is expected. Second, a prospective validating study was not conducted. Third, the study lacks in vitro experiments to verify the miRNA expression level in lung adenocarcinoma tissues. To overcome the above limitations, a prospective multicenter clinical trial in a larger population-based setting is ongoing, and the expression profiles and biological functions of the identified miRNAs need to be validated in vitro in lung adenocarcinoma tissues or in lung adenocarcinoma cell lines.

## Conclusion

In summary, our study profiled circulating sEV miRNAs in patients with IPNs and provided a diagnostic model, the CirsEV-miR model, based on the measurement of circulating sEV miRNAs to distinguish benign PNs from malignant PNs to assist LDCT scanning for early-stage lung cancer diagnosis. Five circulating sEV miRNAs (let-7b-3p, miR-101-3p, miR-125b-5p, miR-150-5p, and miR-3168) were revealed, and the CirsEV-miR model consisting of these miRNAs was established and validated in 208 patients with IPNs, which is the largest sample size so far. Moreover, CirsEV-miR could discriminate between benign and malignant PNs with diameter ≤ 1 cm, which are mostly difficult to distinguish in clinical setting. We primarily revealed that the molecular feature of AIS/MIA was intermediate of benign and invasive PNs, while exhibiting its own characteristics. These results suggest that circulating sEV miRNAs as diagnostic biomarkers could be integrated with LDCT scan to obtain further evaluation work-ups for IPNs.

## Methods

### Patient enrollment and study design

This study was approved by the Institutional Review Board in Shanghai Pulmonary Hospital affiliated with Tongji University (K18-199Y) and registered at the Chinese Clinical Trial Registry (http://www.chictr.org.cn/) with registration number ChiCTR1800019877. All patients were from Shanghai Pulmonary Hospital affiliated with Tongji University and had signed written consents for their blood samples and clinical information to be used in this study.

Patients with IPNs detected using LDCT scanning, who subsequently underwent surgical resections and diagnosed with LUAD (malignant PNs) and various benign PNs, were enrolled in this study. 199 patients with IPNs were recruited in the training phase from April to May 2019, including 20 patients with benign PNs and 179 with malignant PNs diagnosed using pathological examination. 260 patients with IPNs were recruited in the test phase from September to October 2019, including 35 patients with benign PNs and 225 patients with malignant PNs. Plasma samples had been prospectively collected in a vacutainer with anticoagulant (REF367863; Becton Dickinson, Franklin Lakes, NJ, USA) prior to surgical operation. After the elimination of pathology samples from patients with non-LUAD (n = 65), serious hemolysis (above or equal to grade 5, n = 107), failure to meet the ratio of benign and malignant PNs set at 1:2 (n = 155), and failure in construction of sequencing libraries (n = 23), finally, the training cohort consisted of 47 patients with IPNs (17 benign PNs and 30 malignant PNs), and the test cohort consisted of 62 patients with IPNs (24 benign PNs and 38 malignant PNs). In addition, an external cohort consisting of 20 patients with benign PNs and 79 patients with malignant PNs was used for validation. In this case–control study, a variety of benign PNs without selection served as controls. Additionally, 11 healthy people were enrolled as healthy control.The whole study design and inclusion/exclusion criteria are depicted in Fig. 1 and (Additional file 1: Figure S1).

The LUAD consisted of three pathological subtypes, namely, AIS, MIA, and invasive adenocarcinoma. Nine patients were diagnosed with AIS, which is technically not a malignant disease. Considering the perspective of the pathological progression of lung adenocarcinoma, we still classified AIS as a type of malignant nodule. The pathological subtypes of benign PNs were without selection and comprised more than 10 subtypes. The pathological information of all of the samples was obtained from surgically resected tissue sections in accordance with the 2015 WHO Histological Classification of Lung Cancer [26]. The pathological diagnosis of each patient was confirmed by two pathologists. The tumor–node–metastasis (TNM) stage was determined in accordance with the 8th edition International Association for the Study of Lung Cancer (IASLC) lung cancer staging system [41]. The pathological subtypes of our training and test cohorts, and those of the external validation cohort, are shown in (Additional file 2: Figure S2).

The accuracy of a diagnostic test is usually measured by its sensitivity and specificity [42]. In this study, sensitivity represented the model’s ability to correctly identify individuals with malignant PNs, and specificity represented the model’s ability to correctly identify individuals with benign PNs.

### Plasma isolation and sEVs isolation

Blood samples were collected from patients in 10-mL vacutainer tubes containing an anticoagulant of K2EDTA (REF367863; Becton Dickinson, Franklin Lakes, NJ, USA), mixed by gently inverting several times, stored with the tubes placed upright, and then transported on ice within 1 h after collection. To harvest the plasma, the samples were centrifuged at 1600×*g* for 10 min at 4 °C, after which the hemolysis level was determined and recorded. Samples with hemolysis grade of no more than 4 were used [43]. The collected supernatant was centrifuged again at 16,000×*g* for 15 min at 4 °C, and then the 1 mL supernatant was transferred into a fresh 1.5 mL tube and stored at − 80 °C prior to use.

For the sEV isolation from plasma, a polyethylene glycol-based 3D Medicine isolation reagent [18] (L3525; 3DMed, Shanghai, China) was used. This isolation reagent has been modified and improved based on the work of Rider [44], and has been registered to the National Medical Products Administration as a Class I medical device (#HMXB20190091), specifically for the isolation of sEVs in the clinical setting. The plasma samples were centrifuged at 12,000×*g* for 10 min at 4 °C after a static water bath incubation at 37 ℃ for 5 min. The supernatant was transferred to a 0.45 µm tube filter (CLS8163-100EA; Costar, Corning, NY, USA), followed by transfer to a 0.22 µm tube filter (CLS8161-100EA; Costar) and then centrifuged at 12,000×*g* for 5 min at 4 °C. The filtered supernatant was transferred to a fresh 1.5 mL tube. One-quarter volume of an isolation reagent (L3525) was added to the supernatant; gently inverted and incubated for 30 min at 4 °C and then centrifuged at 4700×*g* for 30 min at 4 °C. Finally, the supernatant was removed and the pellets containing the total sEVs were re-suspended in 0.2 mL phosphate-buffered saline (PBS).

### Western blot analysis

The isolated sEVs were lysed in 200 μL lysis buffer (P0013B, Beyotime, Shanghai, China); next, the proteins were extracted using an isolation reagent (N3525, 3DMed, Shanghai, China). The protein concentration of the sEVs was measured using a Pierce™ BCA Protein Assay Kit (Thermo Fisher Scientific, USA). 20 µg of total protein was resolved on a 12% SDS-PAGE gel, electrotransferred onto a PVDF membrane (Millipore, USA). The membranes were blocked in 5% non-fat milk for 60 min, and incubated with anti- CD9 antibody (diluted 1:500; cat. no. ab92726; Abcam, Cambridge, UK), anti-CD63 antibody (1:2000, ab216130; Abcam, Cambridge, UK), anti-Syntenin antibody (diluted 1:500; cat. no. ab19903; Abcam, Cambridge, UK), anti-TSG101 polyclonal antibody (diluted 1:500; cat. no. abs115706; Absin Bioscience Inc., Shanghai, China), and anti-Calnexin antibody (diluted 1:1000; cat. no. 2679; Cell Signaling Technology, Danvers, MA, USA) primary antibodies overnight at 4 °C. Horseradish peroxidase-conjugated goat anti-rabbit IgG and goat anti-mouse IgG antibodies (Beyotime Biotechnology, China) were used as secondary antibodies. Antibody binding was detected using an enhanced chemilluminescence system according to the manufacturer’s protocol (Tanon-5200 Multi; Tanon Science & Technology Co. Ltd., Shanghai, China).

### Nanoparticle tracking analysis (NTA)

Nanosight NS 300 system (NanoSight Technology, Malvern, UK) was used to characterize the number and size of EVs. Isolated sEVs were resuspended in PBS at a concentration of 5 μg/mL and were further diluted 100- to 1000-fold, to achieve between 20 and 100 objects/frame. Samples were manually injected into the sample chamber at ambient temperature. Each sample was configured using a 488 nm laser and a high-sensitivity scientific complementary metal-oxide semiconductor camera, and the measurements were performed in triplicate at a camera setting of 13 with an acquisition time of 30 s and a detection threshold setting of 7. At least 200 completed tracks were analyzed and obtained per video. Finally, the NTA analytical software (version 2.3) was used to analyze the nanoparticle tracking data of the sEV samples in this study.

### Transmission electron microscopy (TEM)

For TEM analysis, plasma sEVs were suspended in PBS prior to fixing in 4% paraformaldehyde and transferred to the carbon-coated electron microscopy grids. They were washed with PBS twice, and the third time with PBS containing glycine (50 mM), each for 3 min; then, they were incubated with PBS containing BSA (0.5%) for 10 min. Finally, the grids were stained with 2% uranyl acetate. After the staining, TEM (H-7650, Hitachi High-Technologies, Japan) was used to analyze the morphology of sEVs.

### ExoView analysis

Plasma sEVs were detected using ExoView chips (NanoView Biosciences, Brighton, MA) printed with antibodies against CD63, CD81, CD9, and mouse IgG1 as a negative control. 35 μL samples were dropped onto the chip and incubated for 16 h. After washing, chips were incubated with a fluorescence antibody cocktail of anti-CD9 (CF^®^ 488), anti- CD81 (CF^®^ 555), and anti-CD63 (CF^®^ 647) for 1 h at room temperature. Chips were then imaged in the ExoView R100 Scanner (NanoView Biosciences, Brighton, MA). Data were analysed using NanoViewer Software (NanoView Biosciences, Brighton, MA).

### RNA isolation from sEVs

RNA was extracted from sEVs using the miRNeasy Serum/Plasma Kit (217184; QIAGEN, Shanghai, China) in accordance with the manufacturer’s protocol. The miRNA quality, yield, and distribution were analyzed using the Agilent 2100 Bioanalyzer with Small RNA Chips (5067-1548; Agilent, Savage, MD, USA).

### Small RNA libraries preparation and sequencing

To prepare and construct the small RNA sequencing libraries, a NEB Next Multiplex Small RNA Library Prep Set for Illumina (E7300L; New England Biolabs, Ipswich, MA, USA) was used in accordance with the manufacturer’s protocol. Briefly, the reverse transcription primer was hybridized after 3ʹ adaptor ligation of 100 ng RNA per sample, following 5ʹ adaptor ligation. A total of 18 PCR cycles were performed with Illumina feasible barcode primers after the first strand cDNA synthesis. The prepared libraries were resolved on NucleoSpin Gel and PCR Clean-up (740609.50; MACHEREY–NAGEL, Germany) and recovered in 30 μL DNase- and RNase-free water. The DNA quality, yield, and distribution were analyzed using the LabChip^®^ GX Touch™ HT Nucleic Acid Analyzer with DNA High Sensitivity Reagent Kit (CLS760672; PerkinElmer, Waltham, MA, USA) and the DNA Extended Range LabChip (CLS138948; PerkinElmer). A total of 20–25 libraries were pooled into a single sequencing lane and sequenced using an Illumina HiSeq PE150 analyzer.

### Bioinformatics analysis of small RNA sequencing data

The 3′ adaptors of reads were cleaved using a custom program. Subsequently, the reads were aligned to the human genome hg19 assembly (http://hgdownload.soe.ucsc.edu/goldenPath/hg19/bigZips/) using BWA 0.7.12 [45]. An individual Small RNA-Seq dataset is required to have a minimum of 5,000,000 reads with minimum mapping rate 80% that mapped with any annotated RNA transcript in the human genome. The annotations were generated from Gencode v25 [46] and miRBase v21 [47] for statistical analysis and to determine expression levels. The annotation includes all small RNAs, such as miRNAs, rRNAs, tRNAs, and piRNAs, as well as long transcripts from GENCODE, which includes both protein coding genes and long non-coding RNAs (lincRNAs). The percentage of reads that mapped to the annotated miRNAs should be greater than 25% (Additional file 1: Table S7). The miRNA expressions were determined by counting the number of reads mapped to the regions annotated by mature miRNAs. The miRNA mapped by at least two reads in each of the samples and with length less than 30 nt was saved for miRNA expression analysis. The miRNA expression analysis was performed using the voom function in the limma package [48], with normalization by Trimmed Mean of M-values (TMM) via the edgeR package, and the miRNA expression level was converted to log2-counts-per-million (logCPM) [49]. The Empirical Bayes algorithm implemented in ComBat was applied to the training and the test cohort data sets adjusted for batch effects [50, 51].

### Quantitative reverse-transcription PCR

Total RNA extraction from sEVs were as previously described. miRNA were reverse transcribed using TaqMan™ Advanced miRNA cfDNA Synthesis Kit (A28007, Applied Biosystems™, USA) according to the manufacturer’s protocol. qPCR was performed on Applied Biosystems 7500 Fast Real-Time PCR systems with specific (miR-451a, miR-125b-5p, miR-101-3p, miR-3168, miR-150-5p and let-7b-3p) probes (A25576, Applied Biosystems™, USA). The expression level of miR-451a were used as control as previously reported [52]. Relative expression were calculated with mean Ct values using 2^−ΔΔCt^ method.

### Statistical analysis

The samples in the training and test cohorts in this study and the samples in the external validation cohort from another study were analyzed [18]. The diagnostic model was constructed using least absolute shrinkage and selection operator (LASSO) in the training cohort. The test cohort and the external cohort were used to test and validate the diagnostic model. We selected the differentially expressed sEV-miRNAs (DEMs) determined according to the stringent statistical threshold (Student’s t-test p-value ≤ 0.05, 1.5-fold change, and the mean expression CPM ≥ 50) between the benign and malignant PNs. Based on DEMs, the risk scores were generated using LASSO analysis, and the best parameters of the model constructed using LASSO were ultimately selected using tenfold cross-validation.

Statistical analysis was performed using the statistical programming language R (version 3.6). The dendextend package [53] in R was used to perform average linkage hierarchical clustering of genes and cases. The heatmap was constructed using the ComplexHeatmap package [54] in R/Bioconductor. The biological processes of Gene Ontology (GO) and the Kyoto Encyclopedia of Genes and Genomes (KEGG) pathways enrichment of the experimentally validated targets of miRNAs were examined using mirPath v.3, which provided the Expression Analysis Systematic Explorer (EASE) score and false-discovery rates using the Fisher’s exact tests and unbiased empirical distributions [55]. The Kaplan–Meier plot analysis of the TCGA data was performed using OncoLnc [56].

## Supplementary Information

**Additional file 1: Figure S1**. Cohorts’ details and inclusion/exclusion criteria. **Figure S2**. Pathology subtypes of the training (a), test (b), and external validation (c) cohorts. **Figure S3**. Representative pathological images of the benign and malignant pulmonary nodule subtypes. Malignant nodule subtypes: adenocarcinoma in situ (AIS), minimally invasive adenocarcinoma (MIA), and invasive adenocarcinoma (IA); Benign nodule subtypes: granulomas, atypical adenomatous hyperplasia (AAH), hamartoma, cyst, fibrosis, organizing pneumonias (OP). Magnification, ×400; Formalin Fixed Paraffin-Embedded (FFPE) tissues. **Figure S4**. CirsEV-miR model performance in the test cohort and the external cohort. **(a)** Circulating sEV miRNA heatmap of the test cohort by unsupervised hierarchical clustering. **(b)** ROC curve of the CirsEV-miR model in the test cohort. **(c)** The CirsEV-miR scores of benign and malignant PNs in the test cohort. **(d)** ROC curve of the CirsEV-miR model in the external validation cohort. **(e)** The CirsEV-miR scores of healthy people and patients with benign or malignant PNs. **(f)** Expression level of the five miRNAs used in CirsEV-miR model. For each group n = 13. All data is presented with mean ± SD, except let-7b-3p which is presented with mean ± SEM. *p < 0.05; **p < 0.01; ***, p < 0.001. Figure S5. CirsEV-miR model performance of IPNs ≤ 1 cm. (a) CirsEV-miR model performance in our cohorts. **(b)** CirsEV-miR model performance in the external validation cohort. **Table S1.** Classifiers constructed from the six identified DEMs. **Table S2.** Performance of classifiers in the training and test cohorts. **Table S3.** The five sEV-miRNAs and their corresponding coefficients in the CirsEV-miR model. **Table S4.** Target genes of the five sEV-miRNAs. **Table S5.** Upregulated DEMs shared between benign PNs, AIS/MIA, and invasive adenocarcinomas. **Table S6.** Downregulated DEMs shared between benign PNs, AIS/MIA, and invasive adenocarcinomas. **Table S7.** Quality control of small RNA sequencing.

## Data Availability

The datasets used during the current study are available from the corresponding author on reasonable request.

## Abbreviations

AAH: Atypical adenomatous hyperplasia
AIS: Adenocarcinoma in situ
AUC: Area under curve
BPs: Biological functions
CPM: Counts-per-million
DEMs: Differentially expressed miRNAs
GO: Gene ontology
IPNs: Indeterminate pulmonary nodules
ISEV: International Society for Extracellular Vesicles
LASSO: Least absolute shrinkage and selection operator
LDCT: Low-dose computed tomography
LUAD: Lung adenocarcinoma
MFs: Molecular functions
MIA: Minimally invasive adenocarcinoma
miRNAs: MicroRNAs
MISEV: Minimal Information for Studies of Extracellular Vesicles
NTA: Nanoparticle tracking analysis
OPs: Organizing pneumonias
OS: Overall survival
PNs: Pulmonary nodules
ROC: Receiver operating characteristic
sEV: Small extracellular vesicles
TCGA: The Cancer Genome Atlas
TEM: Transmission electron microscopy
UTR: Untranslated region
WB: Western blot
