# Supplementary material for: Identification and evaluation of circulating small extracellular vesicle microRNAs as diagnostic biomarkers for patients with indeterminate pulmonary nodules
Source: J Nanobiotechnology. 2022 Apr 2;20:172. doi: 10.1186/s12951-022-01366-0 (PMC8976298; doi:10.1186/s12951-022-01366-0)
Supplement: Supplementary file 1 — Additional file 1: Figure S1. Cohorts’ details and inclusion/exclusion criteria. Figure S2. Pathology subtypes of the training (a), test (b), and external validation (c) cohorts. Figure S3. Representative pathological images of the benign and malignant pulmonary nodule subtypes. Malignant nodule subtypes: adenocarcinoma in situ (AIS), minimally invasive adenocarcinoma (MIA), and invasive adenocarcinoma (IA); Benign nodule subtypes: granulomas, atypical adenomatous hyperplasia (AAH), hamartoma, cyst, fibrosis, organizing pneumonias (OP). Magnification, ×400; Formalin Fixed Paraffin-Embedded (FFPE) tissues. Figure S4. CirsEV-miR model performance in the test cohort and the external cohort. (a) Circulating sEV miRNA heatmap of the test cohort by unsupervised hierarchical clustering. (b) ROC curve of the CirsEV-miR model in the test cohort. (c) The CirsEV-miR scores of benign and malignant PNs in the test cohort. (d) ROC curve of the CirsEV-miR model in the external validation cohort. (e) The CirsEV-miR scores of healthy people and patients with benign or malignant PNs. (f) Expression level of the five miRNAs used in CirsEV-miR model. For each group n = 13. All data is presented with mean ± SD, except let-7b-3p which is presented with mean ± SEM. *p < 0.05; **p < 0.01; ***, p < 0.001. Figure S5. CirsEV-miR model performance of IPNs ≤ 1 cm. (a) CirsEV-miR model performance in our cohorts. (b) CirsEV-miR model performance in the external validation cohort. Table S1. Classifiers constructed from the six identified DEMs. Table S2. Performance of classifiers in the training and test cohorts. Table S3. The five sEV-miRNAs and their corresponding coefficients in the CirsEV-miR model. Table S4. Target genes of the five sEV-miRNAs. Table S5. Upregulated DEMs shared between benign PNs, AIS/MIA, and invasive adenocarcinomas. Table S6. Downregulated DEMs shared between benign PNs, AIS/MIA, and invasive adenocarcinomas. Table S7. Quality control of small RNA sequencing. [file 12951_2022_1366_MOESM1_ESM.pdf]

Figure S1

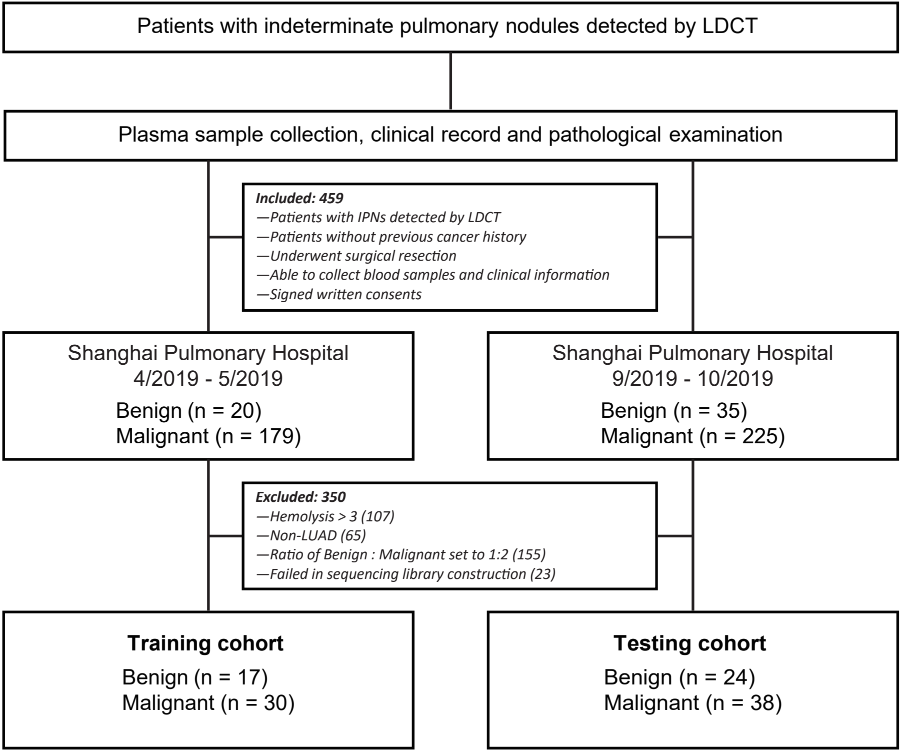
Patients with indeterminate pulmonary nodules detected by LDCT
Plasma sample collection, clinical record and pathological examination
Included: 459
—Patients with IPNs detected by LDCT
—Patients without previous cancer history
—Underwent surgical resection
—Able to collect blood samples and clinical information
—Signed written consents
Shanghai Pulmonary Hospital
4/2019 - 5/2019
Benign (n = 20)
Malignant (n = 179)
Shanghai Pulmonary Hospital
9/2019 - 10/2019
Benign (n = 35)
Malignant (n = 225)
Excluded: 350
—Hemolysis > 3 (107)
—Non-LUAD (65)
—Ratio of Benign : Malignant set to 1:2 (155)
—Failed in sequencing library construction (23)
Training cohort
Benign (n = 17)
Malignant (n = 30)
Testing cohort
Benign (n = 24)
Malignant (n = 38)

Figure S2

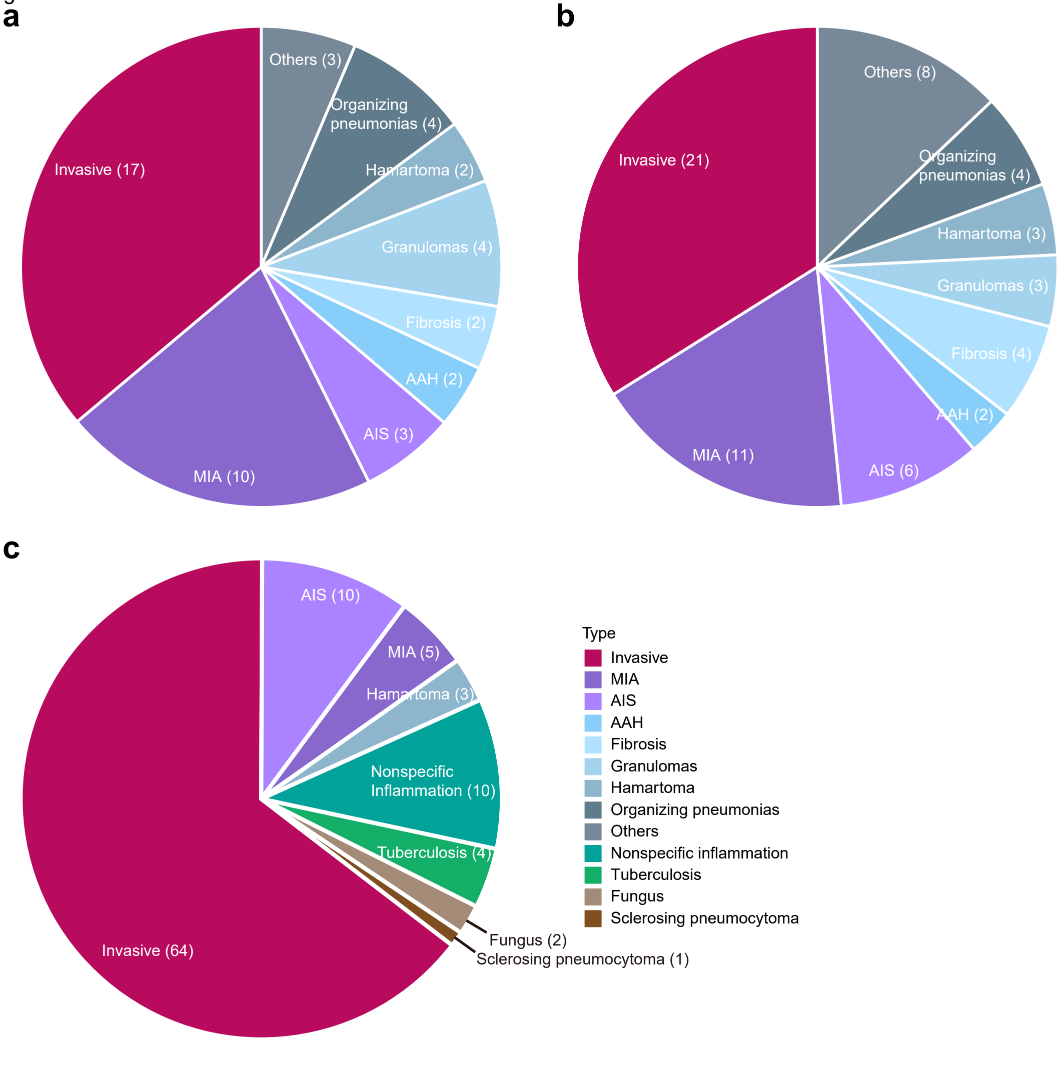
a
Others (3)
Organizing pneumonias (4)
Hamartoma (2)
Granulomas (4)
Fibrosis (2)
AAH (2)
AIS (3)
MIA (10)
Invasive (17)
b
Others (8)
Organizing pneumonias (4)
Hamartoma (3)
Granulomas (3)
Fibrosis (4)
AAH (2)
AIS (6)
MIA (11)
Invasive (21)
c
AIS (10)
MIA (5)
Hamartoma (3)
Nonspecific Inflammation (10)
Tuberculosis (4)
Fungus (2)
Sclerosing pneumocytoma (1)
Invasive (64)
Type
Invasive
MIA
AIS
AAH
Fibrosis
Granulomas
Hamartoma
Organizing pneumonias
Others
Nonspecific inflammation
Tuberculosis
Fungus
Sclerosing pneumocytoma

Figure S3

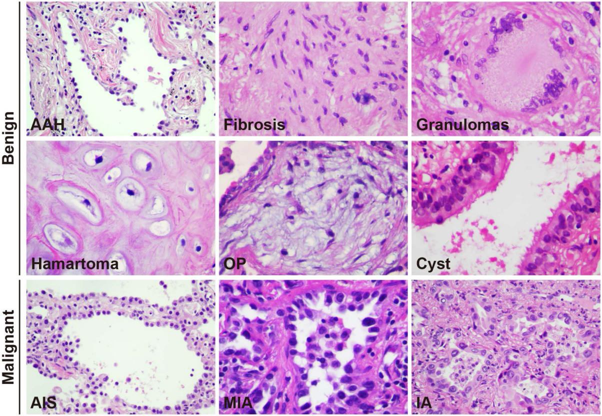

Benign
Malignant
AAH
Fibrosis
Granulomas
Hamartoma
OP
Cyst
AIS
MIA
IA

Figure S4

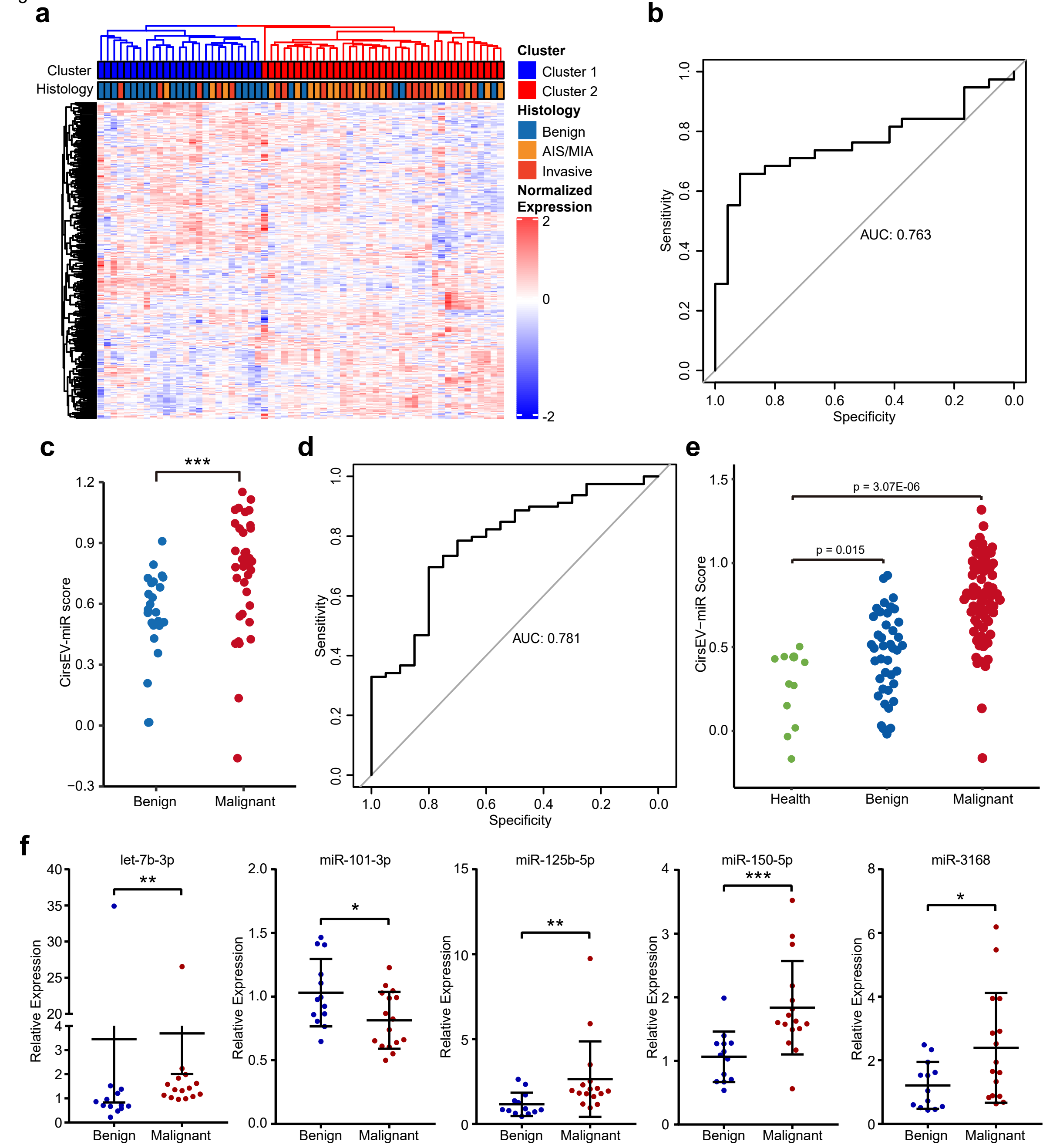

a
Cluster
Histology
Cluster
Cluster 1
Cluster 2
Histology
Benign
AIS/MIA
Invasive
Normalized Expression
2
0
-2
b
Sensitivity
Specificity
AUC: 0.763
c
***
CirsEV-miR score
Benign
Malignant
d
Sensitivity
Specificity
AUC: 0.781
e
p = 3.07E-06
p = 0.015
CirsEV−miR Score
Health
Benign
Malignant
f
let-7b-3p
miR-101-3p
miR-125b-5p
miR-150-5p
miR-3168
Relative Expression
**
*
**
***
*
Benign
Malignant

Figure S5

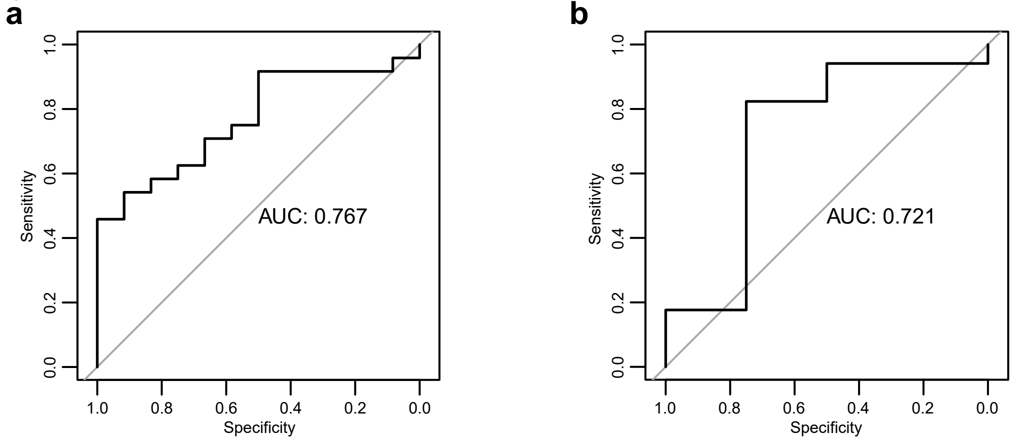
a
1.0
0.8
0.6
0.4
0.2
0.0
Sensitivity
AUC: 0.767
1.0
0.8
0.6
0.4
0.2
0.0
Specificity
b
1.0
0.8
0.6
0.4
0.2
0.0
Sensitivity
AUC: 0.721
1.0
0.8
0.6
0.4
0.2
0.0
Specificity

Table S1. Classifiers constructed from six identified DEMs.

Classifier	Elements
One-miRNA	let-7b-3p
Two-miRNA	let-7b-3p, miR-125b-5p
Three-miRNA	let-7b-3p, miR-125b-5p, miR-197-3p
Four-miRNA	let-7b-3p, miR-125b-5p, miR-197-3p, miR-150-5p
Five-miRNA	let-7b-3p, miR-125b-5p, miR-197-3p, miR-150-5p, miR-3168
Six-miRNA	let-7b-3p, miR-125b-5p, miR-197-3p, miR-150-5p, miR-3168, miR-101-3p

Table S2. Performance of classifiers in the training and test cohorts.

Classifier	Training cohort			Testing cohort		
	AUC	Sensitivity	Specificity	AUC	Sensitivity	Specificity
One-miRNA	0.875	0.667	1.000	0.680	0.737	0.583
Two-miRNA	0.886	0.933	0.824	0.754	0.632	0.833
Three-miRNA	0.892	0.800	0.882	0.773	0.658	0.833
Four-miRNA	0.882	0.800	0.882	0.720	0.684	0.792
Five-miRNA	0.904	0.800	0.941	0.743	0.632	0.917
Six-miRNA	0.794	0.833	0.706	0.729	0.684	0.833
CirsEV-miR	0.920	0.900	0.882	0.763	0.658	0.917

Table S3. The five sEV-miRNAs and their corresponding coefficient in the CirsEV-miR model.

Intercept	-1.75739839
let-7b-3p	0.28001472
miR-101-3p	-0.09222138
miR-125b-5p	0.12801717
miR-150-5p	0.05133272
miR-3168	0.06252532

Table S4. Target genes of the five sEV-miRNAs

Gene Name	Gene Ensembl id	Tarbase Methods
SLK	ENSG00000065613	HITS-CLIP
IRS2	ENSG00000185950	HITS-CLIP
BRAF	ENSG00000157764	HITS-CLIP
POU2F1	ENSG00000143190	PAR-CLIP
CNOT6	ENSG00000113300	HITS-CLIP
ASAH1	ENSG00000104763	HITS-CLIP
GSK3B	ENSG00000082701	Multiple
C5orf30	ENSG00000181751	HITS-CLIP
CCAR2	ENSG00000158941	HITS-CLIP
ERBB2IP	ENSG00000112851	HITS-CLIP
TOB2	ENSG00000183864	HITS-CLIP
LEPROTL1	ENSG00000104660	HITS-CLIP
FEM1B	ENSG00000169018	HITS-CLIP
TROVE2	ENSG00000116747	HITS-CLIP
ZNF273	ENSG00000198039	Multiple
ZNF616	ENSG00000204611	HITS-CLIP
TTYH3	ENSG00000136295	HITS-CLIP
ITGB1	ENSG00000150093	HITS-CLIP
YAP1	ENSG00000137693	Multiple
WASL	ENSG00000106299	HITS-CLIP
CDS2	ENSG00000101290	HITS-CLIP
ANGEL2	ENSG00000174606	HITS-CLIP
STXBP5L	ENSG00000145087	HITS-CLIP
RHOBTB3	ENSG00000164292	PAR-CLIP
PTPLB	ENSG00000206527	HITS-CLIP
NUCKS1	ENSG00000069275	HITS-CLIP
TPM1	ENSG00000140416	HITS-CLIP
USP25	ENSG00000155313	HITS-CLIP
KBTBD8	ENSG00000163376	HITS-CLIP
MGAT5	ENSG00000152127	HITS-CLIP
ADORA2B	ENSG00000170425	HITS-CLIP
CD59	ENSG00000085063	HITS-CLIP
ELK4	ENSG00000158711	HITS-CLIP
RAI1	ENSG00000108557	HITS-CLIP
NUP153	ENSG00000124789	HITS-CLIP
ARF3	ENSG00000134287	HITS-CLIP
THBS1	ENSG00000137801	Multiple
STOM	ENSG00000148175	Multiple
LGR4	ENSG00000205213	HITS-CLIP
ANKRD52	ENSG00000139645	HITS-CLIP
LRRC8B	ENSG00000197147	HITS-CLIP

PAFAH1B1	ENSG00000007168	HITS-CLIP
NAP1L1	ENSG00000187109	HITS-CLIP
ZDHHC20	ENSG00000180776	HITS-CLIP
TMEM135	ENSG00000166575	HITS-CLIP
ALDH1A3	ENSG00000184254	HITS-CLIP
RABGAP1	ENSG00000011454	HITS-CLIP
TTC33	ENSG00000113638	HITS-CLIP
ARPP19	ENSG00000128989	HITS-CLIP
SPRED1	ENSG00000166068	HITS-CLIP
PTPN14	ENSG00000152104	HITS-CLIP
BACH1	ENSG00000156273	HITS-CLIP
ITCH	ENSG00000078747	HITS-CLIP
WAC	ENSG00000095787	Multiple
DNAJB5	ENSG00000137094	HITS-CLIP
PBX1	ENSG00000185630	HITS-CLIP
IRGQ	ENSG00000167378	HITS-CLIP
BPTF	ENSG00000171634	HITS-CLIP
ITGA3	ENSG00000005884	HITS-CLIP
PLCL2	ENSG00000154822	HITS-CLIP
CCT4	ENSG00000115484	HITS-CLIP
VPS36	ENSG00000136100	HITS-CLIP
APPBP2	ENSG00000062725	HITS-CLIP
TMED5	ENSG00000117500	HITS-CLIP
RHPN2	ENSG00000131941	HITS-CLIP
PHB	ENSG00000167085	HITS-CLIP
HBP1	ENSG00000105856	HITS-CLIP
DSTYK	ENSG00000133059	HITS-CLIP
TPBG	ENSG00000146242	HITS-CLIP
ZC3H8	ENSG00000144161	PAR-CLIP
ATP11B	ENSG00000058063	HITS-CLIP
RALBP1	ENSG00000017797	HITS-CLIP
KIF13A	ENSG00000137177	HITS-CLIP
PPP6C	ENSG00000119414	HITS-CLIP
DCUN1D4	ENSG00000109184	HITS-CLIP
MEF2D	ENSG00000116604	HITS-CLIP
MCU	ENSG00000156026	HITS-CLIP
RAB21	ENSG00000080371	HITS-CLIP
SETD5	ENSG00000168137	HITS-CLIP
ZNF736	ENSG00000234444	HITS-CLIP
OSBPL3	ENSG00000070882	HITS-CLIP
KPNA2	ENSG00000182481	HITS-CLIP
ZFHX3	ENSG00000140836	HITS-CLIP
ARF1	ENSG00000143761	HITS-CLIP
TMOD3	ENSG00000138594	HITS-CLIP

NOTCH2	ENSG00000134250	HITS-CLIP
PTMS	ENSG00000159335	HITS-CLIP
GLS	ENSG00000115419	HITS-CLIP
VCL	ENSG00000035403	HITS-CLIP
ARFGEF2	ENSG00000124198	HITS-CLIP
PPP2R5C	ENSG00000078304	HITS-CLIP
RHOA	ENSG00000067560	HITS-CLIP
SEC61A1	ENSG00000058262	HITS-CLIP
CACUL1	ENSG00000151893	HITS-CLIP
GTF2B	ENSG00000137947	HITS-CLIP
CHD7	ENSG00000171316	HITS-CLIP
MED17	ENSG00000042429	HITS-CLIP
PMP22	ENSG00000109099	HITS-CLIP
IPO7	ENSG00000205339	HITS-CLIP
DDX3X	ENSG00000215301	HITS-CLIP
RSRP1	ENSG00000117616	HITS-CLIP
FSTL1	ENSG00000163430	HITS-CLIP
MARK3	ENSG00000075413	HITS-CLIP
FKBP1A	ENSG00000088832	HITS-CLIP
CALM2	ENSG00000143933	HITS-CLIP
MTMR12	ENSG00000150712	HITS-CLIP
ASF1B	ENSG00000105011	HITS-CLIP
ZNF107	ENSG00000196247	HITS-CLIP
NCOA5	ENSG00000124160	HITS-CLIP
CDK2AP1	ENSG00000111328	HITS-CLIP
PDS5B	ENSG00000083642	Multiple
APP	ENSG00000142192	Multiple
CKAP5	ENSG00000175216	HITS-CLIP
TAOK1	ENSG00000160551	Multiple
TAB3	ENSG00000157625	HITS-CLIP
KBTBD6	ENSG00000165572	HITS-CLIP
TMEM123	ENSG00000152558	HITS-CLIP
PLEKHA8	ENSG00000106086	HITS-CLIP
RNF220	ENSG00000187147	HITS-CLIP
RPS6KA5	ENSG00000100784	HITS-CLIP
TNRC6B	ENSG00000100354	HITS-CLIP
ZSWIM6	ENSG00000130449	HITS-CLIP
PTER	ENSG00000165983	HITS-CLIP
ZNF548	ENSG00000188785	HITS-CLIP
PRDM1	ENSG00000057657	HITS-CLIP
TMEM66	ENSG00000133872	HITS-CLIP
ETV5	ENSG00000244405	HITS-CLIP
ASAP1	ENSG00000153317	HITS-CLIP
SLC7A1	ENSG00000139514	HITS-CLIP

ICE1	ENSG00000164151	HITS-CLIP
FOXP1	ENSG00000114861	HITS-CLIP
TES	ENSG00000135269	HITS-CLIP
ZBTB37	ENSG00000185278	HITS-CLIP
LYPD3	ENSG00000124466	HITS-CLIP
ETNK1	ENSG00000139163	HITS-CLIP
PEG10	ENSG00000242265	HITS-CLIP
ZNF217	ENSG00000171940	HITS-CLIP
ITGAV	ENSG00000138448	HITS-CLIP
LARP4B	ENSG00000107929	HITS-CLIP
CROT	ENSG00000005469	HITS-CLIP
PSD3	ENSG00000156011	HITS-CLIP
RNF145	ENSG00000145860	HITS-CLIP
GPR37	ENSG00000170775	HITS-CLIP
NFAT5	ENSG00000102908	HITS-CLIP
PPP1R12A	ENSG00000058272	HITS-CLIP
TET3	ENSG00000187605	HITS-CLIP
KLHDC10	ENSG00000128607	PAR-CLIP
SLC5A3	ENSG00000198743	HITS-CLIP
HNRNPAB	ENSG00000197451	HITS-CLIP
CLIP1	ENSG00000130779	HITS-CLIP
PHC3	ENSG00000173889	HITS-CLIP
FBN1	ENSG00000166147	HITS-CLIP
ATP6AP2	ENSG00000182220	HITS-CLIP
FNDC3B	ENSG00000075420	HITS-CLIP
CTNNB1	ENSG00000168036	HITS-CLIP
PUM1	ENSG00000134644	Multiple
ACTR3	ENSG00000115091	Multiple
GFPT1	ENSG00000198380	Multiple
FAR1	ENSG00000197601	HITS-CLIP
WWC3	ENSG00000047644	HITS-CLIP
NUFIP2	ENSG00000108256	Multiple
ST8SIA4	ENSG00000113532	Multiple
DYNC1LI2	ENSG00000135720	Multiple
VPS37A	ENSG00000155975	HITS-CLIP
UBE3A	ENSG00000114062	Multiple
ATL2	ENSG00000119787	HITS-CLIP
HERC2	ENSG00000128731	HITS-CLIP
CSNK1A1	ENSG00000113712	HITS-CLIP
CREBRF	ENSG00000164463	HITS-CLIP
MYC	ENSG00000136997	Multiple
TEX30	ENSG00000151287	HITS-CLIP
DEDD	ENSG00000158796	HITS-CLIP
NR4A3	ENSG00000119508	HITS-CLIP

AHCTF1	ENSG00000153207	HITS-CLIP
AHDC1	ENSG00000126705	HITS-CLIP
CCL7	ENSG00000108688	HITS-CLIP
KLF3	ENSG00000109787	HITS-CLIP
ARID1A	ENSG00000117713	HITS-CLIP
TFRC	ENSG00000072274	HITS-CLIP
RNF34	ENSG00000170633	HITS-CLIP
RPS16	ENSG00000105193	HITS-CLIP
SETD8	ENSG00000183955	HITS-CLIP
ZC3H12C	ENSG00000149289	HITS-CLIP
ECT2	ENSG00000114346	HITS-CLIP
SET	ENSG00000119335	HITS-CLIP
SLC38A2	ENSG00000134294	HITS-CLIP
RNF19A	ENSG00000034677	HITS-CLIP
RAB11A	ENSG00000103769	HITS-CLIP
RAB18	ENSG00000099246	HITS-CLIP
FOSL2	ENSG00000075426	Multiple
C5orf51	ENSG00000205765	HITS-CLIP
PTGS2	ENSG00000073756	HITS-CLIP
PABPC1	ENSG00000070756	HITS-CLIP
RBM47	ENSG00000163694	HITS-CLIP
CSNK2A1	ENSG00000101266	HITS-CLIP
TMPO	ENSG00000120802	HITS-CLIP
FAM3C	ENSG00000196937	HITS-CLIP
OTUD4	ENSG00000164164	HITS-CLIP
DNAJB14	ENSG00000164031	HITS-CLIP
FSCN1	ENSG00000075618	Multiple
TRIO	ENSG00000038382	HITS-CLIP
CS	ENSG00000062485	HITS-CLIP
RDH10	ENSG00000121039	Multiple
TSC22D2	ENSG00000196428	Multiple
PDCL	ENSG00000136940	HITS-CLIP
IGF2BP3	ENSG00000136231	HITS-CLIP
ZC3H11A	ENSG00000058673	HITS-CLIP
ADNP	ENSG00000101126	HITS-CLIP
CCNY	ENSG00000108100	HITS-CLIP
CD2BP2	ENSG00000169217	HITS-CLIP
FGF5	ENSG00000138675	HITS-CLIP
YY1	ENSG00000100811	HITS-CLIP
ARID5B	ENSG00000150347	Multiple
COL1A2	ENSG00000164692	HITS-CLIP
SSR3	ENSG00000114850	HITS-CLIP
UBE2O	ENSG00000175931	HITS-CLIP
ARHGAP29	ENSG00000137962	HITS-CLIP

ARRDC3	ENSG00000113369	HITS-CLIP
NFE2L2	ENSG00000116044	HITS-CLIP
UBE2H	ENSG00000186591	HITS-CLIP
PRKAR1A	ENSG00000108946	HITS-CLIP
AMOTL2	ENSG00000114019	HITS-CLIP
MGEA5	ENSG00000198408	Multiple
PUM2	ENSG00000055917	Multiple
ATXN7L3B	ENSG00000253719	HITS-CLIP
AHR	ENSG00000106546	HITS-CLIP
REV3L	ENSG00000009413	HITS-CLIP
GOLGA1	ENSG00000136935	HITS-CLIP
MAGI1	ENSG00000151276	HITS-CLIP
EPM2AIP1	ENSG00000178567	PAR-CLIP
RBBP7	ENSG00000102054	HITS-CLIP
SOD2	ENSG00000112096	Multiple
AKAP13	ENSG00000170776	HITS-CLIP
PJA2	ENSG00000198961	HITS-CLIP
RTF1	ENSG00000137815	HITS-CLIP
UBR7	ENSG00000012963	HITS-CLIP
RLF	ENSG00000117000	HITS-CLIP
FGD4	ENSG00000139132	HITS-CLIP
BASP1	ENSG00000176788	HITS-CLIP
RAB22A	ENSG00000124209	HITS-CLIP
ENPP5	ENSG00000112796	HITS-CLIP
PPIP5K2	ENSG00000145725	HITS-CLIP
USP9X	ENSG00000124486	HITS-CLIP
WDR43	ENSG00000163811	HITS-CLIP
HNRNPA2B1	ENSG00000122566	HITS-CLIP
HIPK1	ENSG00000163349	HITS-CLIP
RAB11B	ENSG00000185236	Multiple
KCTD15	ENSG00000153885	HITS-CLIP
ZNF367	ENSG00000165244	PAR-CLIP
TSN	ENSG00000211460	HITS-CLIP
ZDHHC17	ENSG00000186908	HITS-CLIP
NCKAP1	ENSG00000061676	Multiple
KHNYN	ENSG00000100441	HITS-CLIP
CTDSPL2	ENSG00000137770	HITS-CLIP
HEBP2	ENSG00000051620	HITS-CLIP
CLDN12	ENSG00000157224	HITS-CLIP
ITGA6	ENSG00000091409	HITS-CLIP
VAV3	ENSG00000134215	HITS-CLIP
LSM14B	ENSG00000149657	HITS-CLIP
CPEB3	ENSG00000107864	HITS-CLIP
ZC3H4	ENSG00000130749	HITS-CLIP

6-Mar	ENSG00000145495	HITS-CLIP
RAD21	ENSG00000164754	HITS-CLIP
ESRP1	ENSG00000104413	HITS-CLIP
SCARB2	ENSG00000138760	HITS-CLIP
KMT2C	ENSG00000055609	HITS-CLIP
BTG1	ENSG00000133639	Multiple
SLFN5	ENSG00000166750	HITS-CLIP
PARD6B	ENSG00000124171	HITS-CLIP
SLC23A2	ENSG00000089057	HITS-CLIP
DIS3	ENSG00000083520	HITS-CLIP
C20orf194	ENSG00000088854	HITS-CLIP
KLF6	ENSG00000067082	HITS-CLIP
GPR176	ENSG00000166073	HITS-CLIP
CBX5	ENSG00000094916	Multiple
SETX	ENSG00000107290	Multiple
C3orf52	ENSG00000114529	HITS-CLIP
RAB14	ENSG00000119396	Multiple
TFG	ENSG00000114354	PAR-CLIP
GOLIM4	ENSG00000173905	HITS-CLIP
UGDH	ENSG00000109814	HITS-CLIP
NIN	ENSG00000100503	HITS-CLIP
N4BP1	ENSG00000102921	HITS-CLIP
BMPR2	ENSG00000204217	HITS-CLIP
ARHGAP12	ENSG00000165322	HITS-CLIP
BCL2L11	ENSG00000153094	HITS-CLIP
ERLEC1	ENSG00000068912	HITS-CLIP
TFAM	ENSG00000108064	HITS-CLIP
COL4A1	ENSG00000187498	HITS-CLIP
SYNCRIP	ENSG00000135316	HITS-CLIP
ASAP2	ENSG00000151693	Multiple
INO80D	ENSG00000114933	HITS-CLIP
EFNA1	ENSG00000169242	HITS-CLIP
ZNF670	ENSG00000277462	HITS-CLIP
MARCKS	ENSG00000277443	HITS-CLIP
MLLT6	ENSG00000275023	HITS-CLIP
ARHGAP1	ENSG00000175220	HITS-CLIP
RNF41	ENSG00000181852	PAR-CLIP
CCNT2	ENSG00000082258	Multiple
VAPB	ENSG00000124164	HITS-CLIP
B2M	ENSG00000166710	HITS-CLIP
CELF2	ENSG00000048740	HITS-CLIP
SRRT	ENSG00000087087	HITS-CLIP
RNF114	ENSG00000124226	HITS-CLIP
HSPA13	ENSG00000155304	PAR-CLIP

RNF11	ENSG00000123091	Multiple
POU2F1	ENSG00000143190	Multiple
ANP32E	ENSG00000143401	Multiple
FASN	ENSG00000169710	HITS-CLIP
CCNG1	ENSG00000113328	PAR-CLIP
EIF3L	ENSG00000100129	PAR-CLIP
SLC38A1	ENSG00000111371	HITS-CLIP
LONRF3	ENSG00000175556	Multiple
PRLR	ENSG00000113494	HITS-CLIP
PPP1R2	ENSG00000184203	Multiple
NEK7	ENSG00000151414	Multiple
MSI2	ENSG00000153944	HITS-CLIP
TENM3	ENSG00000218336	HITS-CLIP
SEC16A	ENSG00000148396	HITS-CLIP
FOS	ENSG00000170345	HITS-CLIP
PDLIM5	ENSG00000163110	HITS-CLIP
MLK4	ENSG00000143674	Multiple
ZMAT3	ENSG00000172667	PAR-CLIP
NETO2	ENSG00000171208	PAR-CLIP
CNOT6	ENSG00000113300	PAR-CLIP
BICD2	ENSG00000185963	Multiple
GSK3B	ENSG00000082701	PAR-CLIP
RNF44	ENSG00000146083	PAR-CLIP
RBM25	ENSG00000119707	Multiple
ARAP2	ENSG00000047365	PAR-CLIP
FBXL3	ENSG00000005812	PAR-CLIP
WAPAL	ENSG00000062650	HITS-CLIP
PKIA	ENSG00000171033	PAR-CLIP
RBL2	ENSG00000103479	PAR-CLIP
DAZAP2	ENSG00000183283	PAR-CLIP
ITPRIP	ENSG00000148841	HITS-CLIP
AVPI1	ENSG00000119986	PAR-CLIP
RBM27	ENSG00000091009	HITS-CLIP
VWA9	ENSG00000138614	PAR-CLIP
UGCG	ENSG00000148154	HITS-CLIP
TNPO1	ENSG00000083312	Multiple
EFCAB14	ENSG00000159658	Multiple
UBE2Z	ENSG00000159202	Multiple
TVP23B	ENSG00000171928	HITS-CLIP
ZNF283	ENSG00000167637	PAR-CLIP
CDH2	ENSG00000170558	PAR-CLIP
G3BP1	ENSG00000145907	PAR-CLIP
HSPA1A	ENSG00000204389	Multiple
RPS23	ENSG00000186468	PAR-CLIP

PDCD4	ENSG00000150593	HITS-CLIP
MASTL	ENSG00000120539	PAR-CLIP
ADAM10	ENSG00000137845	HITS-CLIP
KL	ENSG00000133116	Multiple
EMP1	ENSG00000134531	HITS-CLIP
OTUD3	ENSG00000169914	Multiple
DSC2	ENSG00000134755	HITS-CLIP
IRF4	ENSG00000137265	PAR-CLIP
PDHX	ENSG00000110435	HITS-CLIP
RAP2B	ENSG00000181467	Multiple
TCP11L2	ENSG00000166046	HITS-CLIP
GTF2E2	ENSG00000197265	Multiple
MEGF9	ENSG00000106780	HITS-CLIP
UBP1	ENSG00000153560	HITS-CLIP
RP4-695O20__B.10	ENSG00000197182	PAR-CLIP
ANO6	ENSG00000177119	HITS-CLIP
RPL17	ENSG00000265681	PAR-CLIP
GRAMD1B	ENSG00000023171	PAR-CLIP
NUP62	ENSG00000213024	Multiple
BCL9	ENSG00000116128	PAR-CLIP
CIC	ENSG00000079432	Multiple
FBXL5	ENSG00000118564	PAR-CLIP
ZBED4	ENSG00000100426	PAR-CLIP
TPD52	ENSG00000076554	Multiple
AREL1	ENSG00000119682	PAR-CLIP
ATXN7L3	ENSG00000087152	HITS-CLIP
FHL1	ENSG00000022267	PAR-CLIP
ZBTB38	ENSG00000177311	HITS-CLIP
PLA2R1	ENSG00000153246	HITS-CLIP
PLEC	ENSG00000178209	HITS-CLIP
ANKRD11	ENSG00000167522	PAR-CLIP
ITGB8	ENSG00000105855	Multiple
NUCKS1	ENSG00000069275	Multiple
CLASP2	ENSG00000163539	Multiple
UBAC2	ENSG00000134882	HITS-CLIP
ZNF12	ENSG00000164631	Multiple
RAD23B	ENSG00000119318	Multiple
SMAD2	ENSG00000175387	PAR-CLIP
CAMSAP1	ENSG00000130559	PAR-CLIP
SKOR1	ENSG00000188779	HITS-CLIP
DICER1	ENSG00000100697	Multiple
CBX2	ENSG00000173894	HITS-CLIP
DNM2	ENSG00000079805	HITS-CLIP
EN2	ENSG00000164778	PAR-CLIP

SCAI	ENSG00000173611	PAR-CLIP
MAST4	ENSG00000069020	HITS-CLIP
TMEM33	ENSG00000109133	Multiple
CBFA2T2	ENSG00000078699	Multiple
MMGT1	ENSG00000169446	PAR-CLIP
NRAS	ENSG00000213281	HITS-CLIP
DNAJC3	ENSG00000102580	HITS-CLIP
LPHN2	ENSG00000117114	Multiple
HN1	ENSG00000189159	Multiple
MTHFD2	ENSG00000065911	Multiple
THUMPD3	ENSG00000134077	PAR-CLIP
PPP2R3A	ENSG00000073711	HITS-CLIP
MAP7D3	ENSG00000129680	PAR-CLIP
PRRC1	ENSG00000164244	PAR-CLIP
CD38	ENSG00000004468	PAR-CLIP
CHST15	ENSG00000182022	HITS-CLIP
CLIC4	ENSG00000169504	HITS-CLIP
COPS8	ENSG00000198612	PAR-CLIP
MSMO1	ENSG00000052802	PAR-CLIP
KBTBD2	ENSG00000170852	Multiple
CHD1	ENSG00000153922	PAR-CLIP
KIF1B	ENSG00000054523	PAR-CLIP
ABT1	ENSG00000146109	Multiple
SPPL2A	ENSG00000138600	HITS-CLIP
TMED7-TICAM2	ENSG00000251201	PAR-CLIP
RAB3D	ENSG00000105514	HITS-CLIP
DR1	ENSG00000117505	HITS-CLIP
FAM78A	ENSG00000126882	PAR-CLIP
EXOC5	ENSG00000070367	HITS-CLIP
FBXO30	ENSG00000118496	Multiple
ERO1L	ENSG00000197930	Multiple
C16orf87	ENSG00000155330	PAR-CLIP
FAM115A	ENSG00000198420	HITS-CLIP
YOD1	ENSG00000180667	Multiple
HEATR6	ENSG00000068097	Multiple
TMED7	ENSG00000134970	PAR-CLIP
SMAD9	ENSG00000120693	PAR-CLIP
RRN3	ENSG00000085721	PAR-CLIP
SLC25A3	ENSG00000075415	Multiple
SOX11	ENSG00000176887	HITS-CLIP
PTPN9	ENSG00000169410	PAR-CLIP
CRK	ENSG00000167193	HITS-CLIP
KIAA1598	ENSG00000187164	HITS-CLIP
FBXW7	ENSG00000109670	Multiple

GATA3	ENSG00000107485	Multiple
TRIM37	ENSG00000108395	Multiple
COL12A1	ENSG00000111799	HITS-CLIP
TRIAP1	ENSG00000170855	PAR-CLIP
ARRDC4	ENSG00000140450	HITS-CLIP
THBS1	ENSG00000137801	HITS-CLIP
RUNX1	ENSG00000159216	PAR-CLIP
TMEM167A	ENSG00000174695	Multiple
RPL3	ENSG00000100316	PAR-CLIP
MTMR6	ENSG00000139505	PAR-CLIP
VANGL1	ENSG00000173218	PAR-CLIP
PHACTR2	ENSG00000112419	HITS-CLIP
UBTD2	ENSG00000168246	PAR-CLIP
OPN3	ENSG00000054277	PAR-CLIP
AGO3	ENSG00000126070	Multiple
PIP5K1C	ENSG00000186111	Multiple
MEX3B	ENSG00000183496	PAR-CLIP
CLCN3	ENSG00000109572	HITS-CLIP
PAK2	ENSG00000180370	HITS-CLIP
C11orf30	ENSG00000158636	Multiple
SAMD8	ENSG00000156671	PAR-CLIP
PVRL2	ENSG00000130202	HITS-CLIP
SRP19	ENSG00000153037	HITS-CLIP
SPATA2	ENSG00000158480	Multiple
TADA2B	ENSG00000173011	Multiple
ELK3	ENSG00000111145	Multiple
SETD7	ENSG00000145391	Multiple
LMNB2	ENSG00000176619	Multiple
USP37	ENSG00000135913	Multiple
SEL1L	ENSG00000071537	HITS-CLIP
FAM175A	ENSG00000163322	PAR-CLIP
CPEB4	ENSG00000113742	PAR-CLIP
HABP4	ENSG00000130956	PAR-CLIP
EXTL3	ENSG00000012232	HITS-CLIP
COPS7B	ENSG00000144524	HITS-CLIP
EXPH5	ENSG00000110723	PAR-CLIP
DIDO1	ENSG00000101191	Multiple
UBQLN1	ENSG00000135018	HITS-CLIP
IFNGR2	ENSG00000159128	Multiple
CSNK1G3	ENSG00000151292	HITS-CLIP
PURA	ENSG00000185129	PAR-CLIP
TEP1	ENSG00000129566	HITS-CLIP
CYP51A1	ENSG00000001630	Multiple
PAFAH1B1	ENSG00000007168	Multiple

ATAD1	ENSG00000138138	HITS-CLIP
SLTM	ENSG00000137776	HITS-CLIP
NAP1L1	ENSG00000187109	Multiple
GCLM	ENSG00000023909	PAR-CLIP
SGPL1	ENSG00000166224	PAR-CLIP
REEP3	ENSG00000165476	Multiple
YWHAG	ENSG00000170027	HITS-CLIP
SEC23A	ENSG00000100934	HITS-CLIP
PPP1CC	ENSG00000186298	Multiple
MCL1	ENSG00000143384	Multiple
PTPN4	ENSG00000088179	HITS-CLIP
MAPK14	ENSG00000112062	HITS-CLIP
GNA13	ENSG00000120063	Multiple
CCND2	ENSG00000118971	HITS-CLIP
NDNL2	ENSG00000185115	PAR-CLIP
ADAT1	ENSG00000065457	HITS-CLIP
TSPAN3	ENSG00000140391	PAR-CLIP
ZNF260	ENSG00000254004	Multiple
MKL2	ENSG00000186260	Multiple
HNRNPDL	ENSG00000152795	PAR-CLIP
HOXA9	ENSG00000078399	Multiple
TM9SF3	ENSG00000077147	PAR-CLIP
RC3H2	ENSG00000056586	Multiple
MTMR4	ENSG00000108389	HITS-CLIP
HNRNPF	ENSG00000169813	Multiple
NIP7	ENSG00000132603	PAR-CLIP
PGM2	ENSG00000169299	HITS-CLIP
ZNF850	ENSG00000267041	PAR-CLIP
BRIP1	ENSG00000136492	HITS-CLIP
ARHGAP17	ENSG00000140750	PAR-CLIP
SYPL1	ENSG00000008282	Multiple
BACH1	ENSG00000156273	Multiple
SAMSN1	ENSG00000155307	PAR-CLIP
CPSF2	ENSG00000165934	Multiple
GNG12	ENSG00000172380	PAR-CLIP
HUWE1	ENSG00000086758	Multiple
FRS2	ENSG00000166225	HITS-CLIP
CBX4	ENSG00000141582	HITS-CLIP
BCL9L	ENSG00000186174	Multiple
STX3	ENSG00000166900	HITS-CLIP
KLF12	ENSG00000118922	Multiple
MRPS12	ENSG00000128626	PAR-CLIP
EPB41L4B	ENSG00000095203	HITS-CLIP
CAMSAP2	ENSG00000118200	Multiple

DIP2B	ENSG00000066084	Multiple
ANKRD50	ENSG00000151458	Multiple
LCOR	ENSG00000196233	Multiple
ACLY	ENSG00000131473	HITS-CLIP
ZC3H6	ENSG00000188177	PAR-CLIP
KIAA0319L	ENSG00000142687	Multiple
FZD6	ENSG00000164930	Multiple
ZNF24	ENSG00000172466	PAR-CLIP
PPP3R1	ENSG00000221823	HITS-CLIP
HIST1H4C	ENSG00000197061	PAR-CLIP
TOB1	ENSG00000141232	HITS-CLIP
UBE2E2	ENSG00000182247	PAR-CLIP
WAC	ENSG00000095787	PAR-CLIP
GAN	ENSG00000261609	Multiple
WNK1	ENSG00000060237	Multiple
GSE1	ENSG00000131149	HITS-CLIP
PMF1	ENSG00000160783	PAR-CLIP
PHF20	ENSG00000025293	PAR-CLIP
B4GALT6	ENSG00000118276	PAR-CLIP
SEMA3C	ENSG00000075223	Multiple
CA12	ENSG00000074410	HITS-CLIP
PREPL	ENSG00000138078	HITS-CLIP
ADO	ENSG00000181915	Multiple
SOD1	ENSG00000142168	PAR-CLIP
TMEM64	ENSG00000180694	HITS-CLIP
EYA4	ENSG00000112319	PAR-CLIP
SLC4A4	ENSG00000080493	PAR-CLIP
CCT5	ENSG00000150753	Multiple
ZNF664	ENSG00000179195	Multiple
ATG4D	ENSG00000130734	PAR-CLIP
IER2	ENSG00000160888	Multiple
ERLIN2	ENSG00000147475	HITS-CLIP
ZSCAN22	ENSG00000182318	PAR-CLIP
URB2	ENSG00000135763	HITS-CLIP
ZNF507	ENSG00000168813	HITS-CLIP
CPSF6	ENSG00000111605	Multiple
APPBP2	ENSG00000062725	PAR-CLIP
SMC3	ENSG00000108055	HITS-CLIP
SLC1A1	ENSG00000106688	PAR-CLIP
TMED5	ENSG00000117500	Multiple
SPG11	ENSG00000104133	HITS-CLIP
DSTYK	ENSG00000133059	Multiple
STYX	ENSG00000198252	HITS-CLIP
AP4E1	ENSG00000081014	HITS-CLIP

MED13	ENSG00000108510	Multiple
CCNF	ENSG00000162063	PAR-CLIP
SNTB2	ENSG00000168807	HITS-CLIP
ADNP2	ENSG00000101544	HITS-CLIP
TMTC3	ENSG00000139324	Multiple
PAPPA	ENSG00000182752	HITS-CLIP
SQLE	ENSG00000104549	HITS-CLIP
RALBP1	ENSG00000017797	Multiple
PTPN1	ENSG00000196396	HITS-CLIP
PPTC7	ENSG00000196850	Multiple
NUP88	ENSG00000108559	HITS-CLIP
HIPK3	ENSG00000110422	HITS-CLIP
KIF13A	ENSG00000137177	Multiple
DCBLD2	ENSG00000057019	Multiple
PAPOLA	ENSG00000090060	PAR-CLIP
DLG5	ENSG00000151208	HITS-CLIP
FZR1	ENSG00000105325	HITS-CLIP
AFF4	ENSG00000072364	HITS-CLIP
ANKRD17	ENSG00000132466	Multiple
STAG2	ENSG00000101972	Multiple
TIMM8A	ENSG00000126953	PAR-CLIP
HNRNPA0	ENSG00000177733	Multiple
N4BP2L2	ENSG00000244754	Multiple
CSDE1	ENSG00000009307	HITS-CLIP
IRF2BP2	ENSG00000168264	HITS-CLIP
DGCR2	ENSG00000070413	HITS-CLIP
RBM22	ENSG00000086589	PAR-CLIP
LMNB1	ENSG00000113368	Multiple
MEF2D	ENSG00000116604	Multiple
HMGB2	ENSG00000164104	Multiple
TNKS2	ENSG00000107854	Multiple
SP3	ENSG00000172845	HITS-CLIP
MOB1B	ENSG00000173542	PAR-CLIP
ATP1B1	ENSG00000143153	Multiple
URI1	ENSG00000105176	Multiple
SERINC3	ENSG00000132824	HITS-CLIP
GPATCH2L	ENSG00000089916	HITS-CLIP
C19orf12	ENSG00000131943	PAR-CLIP
PPP2R5D	ENSG00000112640	HITS-CLIP
ARID4A	ENSG00000032219	HITS-CLIP
ZNF292	ENSG00000188994	PAR-CLIP
WEE1	ENSG00000166483	Multiple
CDK1	ENSG00000170312	PAR-CLIP
SFT2D2	ENSG00000213064	HITS-CLIP

LRP12	ENSG00000147650	PAR-CLIP
PHF3	ENSG00000118482	Multiple
AGFG1	ENSG00000173744	Multiple
IGIP	ENSG00000182700	Multiple
IGF1R	ENSG00000140443	PAR-CLIP
LSM14A	ENSG00000257103	PAR-CLIP
PIK3C2B	ENSG00000133056	HITS-CLIP
FMR1	ENSG00000102081	Multiple
BTBD3	ENSG00000132640	Multiple
PSME4	ENSG00000068878	HITS-CLIP
KPNA2	ENSG00000182481	PAR-CLIP
AGO4	ENSG00000134698	PAR-CLIP
EVI5	ENSG00000067208	Multiple
HSPD1	ENSG00000144381	HITS-CLIP
ARF1	ENSG00000143761	Multiple
FAM174A	ENSG00000174132	HITS-CLIP
NACC1	ENSG00000160877	Multiple
CLTC	ENSG00000141367	Multiple
KPNA4	ENSG00000186432	HITS-CLIP
DNAJC7	ENSG00000168259	Multiple
IFITM3	ENSG00000142089	HITS-CLIP
SQSTM1	ENSG00000161011	PAR-CLIP
TSPAN12	ENSG00000106025	Multiple
ZNF800	ENSG00000048405	HITS-CLIP
ID4	ENSG00000172201	PAR-CLIP
VCL	ENSG00000035403	PAR-CLIP
USP47	ENSG00000170242	HITS-CLIP
GNB1	ENSG00000078369	Multiple
SNN	ENSG00000184602	Multiple
LUC7L3	ENSG00000108848	HITS-CLIP
NHLRC2	ENSG00000196865	Multiple
ETF1	ENSG00000120705	HITS-CLIP
RHOA	ENSG00000067560	Multiple
B3GALNT2	ENSG00000162885	Multiple
PCDH10	ENSG00000138650	HITS-CLIP
MAP3K13	ENSG00000073803	HITS-CLIP
CHD7	ENSG00000171316	PAR-CLIP
TJP1	ENSG00000104067	Multiple
FAM91A1	ENSG00000176853	Multiple
EED	ENSG00000074266	HITS-CLIP
CD2AP	ENSG00000198087	HITS-CLIP
RAB1A	ENSG00000138069	Multiple
NARS	ENSG00000134440	PAR-CLIP
PSAP	ENSG00000197746	HITS-CLIP

TMEM179B	ENSG00000185475	PAR-CLIP
HNRNPU	ENSG00000153187	Multiple
TCF19	ENSG00000137310	PAR-CLIP
HSDL1	ENSG00000103160	Multiple
MGARP	ENSG00000137463	PAR-CLIP
ZKSCAN8	ENSG00000198315	PAR-CLIP
EZH2	ENSG00000106462	Multiple
CDK6	ENSG00000105810	PAR-CLIP
HEG1	ENSG00000173706	Multiple
MAP3K9	ENSG00000006432	PAR-CLIP
NISCH	ENSG00000010322	HITS-CLIP
FYN	ENSG00000010810	PAR-CLIP
7-Sep	ENSG00000122545	PAR-CLIP
SERINC1	ENSG00000111897	HITS-CLIP
NDFIP1	ENSG00000131507	PAR-CLIP
CALM2	ENSG00000143933	PAR-CLIP
PRKCE	ENSG00000171132	HITS-CLIP
SMARCD1	ENSG00000066117	Multiple
MBD1	ENSG00000141644	PAR-CLIP
BUB3	ENSG00000154473	PAR-CLIP
GCC1	ENSG00000179562	PAR-CLIP
MSN	ENSG00000147065	PAR-CLIP
ZCCHC2	ENSG00000141664	PAR-CLIP
RPS20	ENSG00000008988	Multiple
EPC1	ENSG00000120616	HITS-CLIP
CDK2AP1	ENSG00000111328	PAR-CLIP
TANK	ENSG00000136560	Multiple
VHL	ENSG00000134086	HITS-CLIP
PTPN22	ENSG00000134242	HITS-CLIP
MAT2B	ENSG00000038274	HITS-CLIP
7-Mar	ENSG00000136536	PAR-CLIP
LBR	ENSG00000143815	Multiple
DST	ENSG00000151914	Multiple
KIAA2026	ENSG00000183354	PAR-CLIP
AMMECR1L	ENSG00000144233	PAR-CLIP
TMEM65	ENSG00000164983	Multiple
NKX6-1	ENSG00000163623	PAR-CLIP
FOXK2	ENSG00000141568	HITS-CLIP
ZNF746	ENSG00000181220	Multiple
SIKE1	ENSG00000052723	Multiple
ADIPOR2	ENSG00000006831	Multiple
IER3	ENSG00000137331	HITS-CLIP
RFX7	ENSG00000181827	Multiple
FAT4	ENSG00000196159	Multiple

ATP2B1	ENSG00000070961	PAR-CLIP
ANLN	ENSG00000011426	Multiple
FAM96A	ENSG00000166797	Multiple
NSD1	ENSG00000165671	Multiple
RCN2	ENSG00000117906	HITS-CLIP
PLEKHA8	ENSG00000106086	Multiple
NCL	ENSG00000115053	HITS-CLIP
ATP5B	ENSG00000110955	Multiple
FAM222B	ENSG00000173065	Multiple
CHEK1	ENSG00000149554	Multiple
C7orf49	ENSG00000122783	HITS-CLIP
KLF10	ENSG00000155090	HITS-CLIP
MPP5	ENSG00000072415	Multiple
MRFAP1	ENSG00000179010	HITS-CLIP
TMEM181	ENSG00000146433	Multiple
PPP2R5A	ENSG00000066027	PAR-CLIP
MAML3	ENSG00000196782	PAR-CLIP
TNRC6B	ENSG00000100354	Multiple
PDE4D	ENSG00000113448	HITS-CLIP
PRRC2C	ENSG00000117523	HITS-CLIP
ZNF197	ENSG00000186448	PAR-CLIP
MCTS1	ENSG00000232119	HITS-CLIP
DCAF13	ENSG00000164934	HITS-CLIP
ASH1L	ENSG00000116539	PAR-CLIP
TOP2B	ENSG00000077097	HITS-CLIP
ZSWIM6	ENSG00000130449	PAR-CLIP
IMPA1	ENSG00000133731	Multiple
PANK3	ENSG00000120137	Multiple
MTMR7	ENSG00000003987	HITS-CLIP
EDEM1	ENSG00000134109	HITS-CLIP
SRSF1	ENSG00000136450	HITS-CLIP
PURB	ENSG00000146676	Multiple
MRPL35	ENSG00000132313	PAR-CLIP
EVL	ENSG00000196405	HITS-CLIP
HIF1AN	ENSG00000166135	HITS-CLIP
SENP6	ENSG00000112701	HITS-CLIP
NMI	ENSG00000123609	PAR-CLIP
OSBPL9	ENSG00000117859	PAR-CLIP
ZFP82	ENSG00000181007	PAR-CLIP
SLC35F5	ENSG00000115084	PAR-CLIP
TMEM170B	ENSG00000205269	HITS-CLIP
ZNF207	ENSG00000010244	Multiple
RFC1	ENSG00000035928	HITS-CLIP
TRIP12	ENSG00000153827	HITS-CLIP

C19orf82	ENSG00000267106	HITS-CLIP
DYRK2	ENSG00000127334	Multiple
TXNDC16	ENSG00000087301	HITS-CLIP
H2AFV	ENSG00000105968	Multiple
DCAF7	ENSG00000136485	Multiple
CDK8	ENSG00000132964	Multiple
RIOK2	ENSG00000058729	PAR-CLIP
ARL4C	ENSG00000188042	Multiple
C7orf73	ENSG00000243317	PAR-CLIP
HECTD1	ENSG00000092148	Multiple
ASAP1	ENSG00000153317	PAR-CLIP
MAPK1IP1L	ENSG00000168175	HITS-CLIP
GREB1L	ENSG00000141449	Multiple
ACVR2B	ENSG00000114739	PAR-CLIP
EDEM3	ENSG00000116406	Multiple
FAM46A	ENSG00000112773	Multiple
CD81	ENSG00000110651	Multiple
RAB5A	ENSG00000144566	HITS-CLIP
FAM69B	ENSG00000165716	HITS-CLIP
PCGF5	ENSG00000180628	PAR-CLIP
SCAMP1	ENSG00000085365	Multiple
EIF5	ENSG00000100664	Multiple
ARID2	ENSG00000189079	HITS-CLIP
PRICKLE1	ENSG00000139174	PAR-CLIP
ATM	ENSG00000149311	PAR-CLIP
XPO7	ENSG00000130227	PAR-CLIP
SCAP	ENSG00000114650	HITS-CLIP
MAVS	ENSG00000088888	HITS-CLIP
HECTD2	ENSG00000165338	PAR-CLIP
EIF2S3	ENSG00000130741	PAR-CLIP
ISOC1	ENSG00000066583	HITS-CLIP
ETNK1	ENSG00000139163	Multiple
MLEC	ENSG00000110917	Multiple
ARHGAP18	ENSG00000146376	HITS-CLIP
PISD	ENSG00000241878	PAR-CLIP
ZNF143	ENSG00000166478	PAR-CLIP
ZNF217	ENSG00000171940	Multiple
LARP4B	ENSG00000107929	Multiple
ABHD10	ENSG00000144827	PAR-CLIP
ANK3	ENSG00000151150	HITS-CLIP
RASA1	ENSG00000145715	HITS-CLIP
SH3BP4	ENSG00000130147	HITS-CLIP
FH	ENSG00000091483	HITS-CLIP
ARHGEF11	ENSG00000132694	HITS-CLIP

SNX31	ENSG00000174226	PAR-CLIP
DERL2	ENSG00000072849	PAR-CLIP
KANSL1	ENSG00000120071	HITS-CLIP
CERS2	ENSG00000143418	PAR-CLIP
SLC9A1	ENSG00000090020	HITS-CLIP
REPIN1	ENSG00000214022	HITS-CLIP
KIAA1432	ENSG00000107036	Multiple
SMC4	ENSG00000113810	HITS-CLIP
HDGF	ENSG00000143321	HITS-CLIP
ZFX	ENSG00000005889	Multiple
DDIT4	ENSG00000168209	Multiple
SFXN1	ENSG00000164466	PAR-CLIP
TFAP4	ENSG00000090447	Multiple
PIKFYVE	ENSG00000115020	HITS-CLIP
AEBP2	ENSG00000139154	Multiple
TET3	ENSG00000187605	Multiple
CMIP	ENSG00000153815	HITS-CLIP
ZNF655	ENSG00000197343	Multiple
CSNK1D	ENSG00000141551	Multiple
ATP5G3	ENSG00000154518	Multiple
AGO1	ENSG00000092847	PAR-CLIP
AP1S2	ENSG00000182287	Multiple
PCNX	ENSG00000100731	HITS-CLIP
HNRNPAB	ENSG00000197451	Multiple
CLIP1	ENSG00000130779	Multiple
EEF2	ENSG00000167658	PAR-CLIP
TMEM161B	ENSG00000164180	HITS-CLIP
BTG2	ENSG00000159388	Multiple
CASP3	ENSG00000164305	Western Blot
FBN1	ENSG00000166147	Multiple
STX16	ENSG00000124222	PAR-CLIP
PDCD10	ENSG00000114209	HITS-CLIP
PAQR7	ENSG00000182749	PAR-CLIP
AK6	ENSG00000273841	HITS-CLIP
JUN	ENSG00000177606	PAR-CLIP
PPP1R15B	ENSG00000158615	HITS-CLIP
WDR33	ENSG00000136709	PAR-CLIP
DCP2	ENSG00000172795	Multiple
ATP6AP2	ENSG00000182220	Multiple
ZNF649	ENSG00000198093	PAR-CLIP
FANCM	ENSG00000187790	HITS-CLIP
GOLGA8A	ENSG00000175265	Multiple
HMGXB4	ENSG00000100281	PAR-CLIP
SLC20A2	ENSG00000168575	PAR-CLIP

CCND1	ENSG00000110092	Multiple
GRPEL2	ENSG00000164284	Multiple
PFN2	ENSG00000070087	PAR-CLIP
FAM117B	ENSG00000138439	PAR-CLIP
C10orf12	ENSG00000155640	Multiple
UBE2A	ENSG00000077721	Multiple
ZNF678	ENSG00000181450	PAR-CLIP
PUM1	ENSG00000134644	HITS-CLIP
PPP3CB	ENSG00000107758	HITS-CLIP
TBC1D24	ENSG00000162065	PAR-CLIP
SNX27	ENSG00000143376	PAR-CLIP
PTBP3	ENSG00000119314	HITS-CLIP
TUBA1C	ENSG00000167553	PAR-CLIP
GPD2	ENSG00000115159	PAR-CLIP
NR3C1	ENSG00000113580	PAR-CLIP
TAPBP	ENSG00000231925	HITS-CLIP
SSFA2	ENSG00000138434	Multiple
PIGC	ENSG00000135845	HITS-CLIP
KCTD2	ENSG00000180901	PAR-CLIP
PUS7L	ENSG00000129317	HITS-CLIP
IFFO2	ENSG00000169991	HITS-CLIP
SKP2	ENSG00000145604	Multiple
ZNF264	ENSG00000083844	PAR-CLIP
CIAPIN1	ENSG00000005194	HITS-CLIP
PGBD4	ENSG00000182405	PAR-CLIP
GCN1L1	ENSG00000089154	HITS-CLIP
FAR1	ENSG00000197601	PAR-CLIP
TMEM19	ENSG00000139291	Multiple
NEK4	ENSG00000114904	HITS-CLIP
TMEM87A	ENSG00000103978	HITS-CLIP
BLOC1S6	ENSG00000104164	Multiple
SLC19A2	ENSG00000117479	Multiple
EAPP	ENSG00000129518	PAR-CLIP
LRRC58	ENSG00000163428	PAR-CLIP
FAM8A1	ENSG00000137414	Multiple
HELZ	ENSG00000198265	Multiple
MCC	ENSG00000171444	PAR-CLIP
RGMB	ENSG00000174136	PAR-CLIP
SACS	ENSG00000151835	Multiple
C8orf4	ENSG00000176907	HITS-CLIP
ATP5A1	ENSG00000152234	HITS-CLIP
ITM2C	ENSG00000135916	HITS-CLIP
PPAP2B	ENSG00000162407	HITS-CLIP
CSNK1A1	ENSG00000113712	Multiple

CGGBP1	ENSG00000163320	Multiple
LNPEP	ENSG00000113441	HITS-CLIP
CEBPA	ENSG00000245848	PAR-CLIP
SMU1	ENSG00000122692	HITS-CLIP
UBE3C	ENSG00000009335	PAR-CLIP
PARVA	ENSG00000197702	HITS-CLIP
CD46	ENSG00000117335	PAR-CLIP
RAPGEF6	ENSG00000158987	PAR-CLIP
MANF	ENSG00000145050	PAR-CLIP
SMIM15	ENSG00000188725	HITS-CLIP
SACM1L	ENSG00000211456	Multiple
PPM1A	ENSG00000100614	PAR-CLIP
MYO9A	ENSG00000066933	HITS-CLIP
CDC14B	ENSG00000081377	HITS-CLIP
VCAN	ENSG00000038427	HITS-CLIP
USP46	ENSG00000109189	PAR-CLIP
WSB1	ENSG00000109046	HITS-CLIP
WDR36	ENSG00000134987	PAR-CLIP
GPRC5A	ENSG00000013588	PAR-CLIP
LIFR	ENSG00000113594	Multiple
SUB1	ENSG00000113387	Multiple
DIAPH1	ENSG00000131504	Multiple
IGF2	ENSG00000167244	HITS-CLIP
C21orf91	ENSG00000154642	Multiple
ZDHHC4	ENSG00000136247	HITS-CLIP
GOLGA4	ENSG00000144674	HITS-CLIP
KLF3	ENSG00000109787	Multiple
ATXN1	ENSG00000124788	PAR-CLIP
ARID1A	ENSG00000117713	Multiple
DEGS1	ENSG00000143753	PAR-CLIP
CLDND1	ENSG00000080822	Multiple
DENND5B	ENSG00000170456	HITS-CLIP
ZNF778	ENSG00000170100	PAR-CLIP
IGF2R	ENSG00000197081	Multiple
MYPOP	ENSG00000176182	HITS-CLIP
ANKRD42	ENSG00000137494	Multiple
SLC16A1	ENSG00000155380	Multiple
EIF4A2	ENSG00000156976	HITS-CLIP
MTPN	ENSG00000105887	HITS-CLIP
SKIL	ENSG00000136603	Multiple
BIRC6	ENSG00000115760	HITS-CLIP
PCNXL2	ENSG00000135749	PAR-CLIP
DLG3	ENSG00000082458	HITS-CLIP
BMP2K	ENSG00000138756	PAR-CLIP

JRKL	ENSG00000183340	HITS-CLIP
REL	ENSG00000162924	Multiple
CEP350	ENSG00000135837	Multiple
FRMD6	ENSG00000139926	Multiple
HAUS6	ENSG00000147874	HITS-CLIP
MBD2	ENSG00000134046	PAR-CLIP
ARNTL2	ENSG00000029153	PAR-CLIP
ATRX	ENSG00000085224	HITS-CLIP
ZFP41	ENSG00000264668	PAR-CLIP
SPRTN	ENSG00000010072	PAR-CLIP
MAFK	ENSG00000198517	PAR-CLIP
ZNF687	ENSG00000143373	HITS-CLIP
ZMYM2	ENSG00000121741	Multiple
APLP2	ENSG00000084234	Multiple
ENPP2	ENSG00000136960	Multiple
CD86	ENSG00000114013	PAR-CLIP
GDE1	ENSG00000006007	PAR-CLIP
GMPS	ENSG00000163655	PAR-CLIP
HSP90AB1	ENSG00000096384	HITS-CLIP
ZBTB5	ENSG00000168795	PAR-CLIP
MYO5A	ENSG00000197535	Multiple
SLC38A2	ENSG00000134294	Multiple
RNF19A	ENSG00000034677	Multiple
ZNF100	ENSG00000197020	Multiple
PPP4R1	ENSG00000154845	Multiple
NSA2	ENSG00000164346	HITS-CLIP
KDM3B	ENSG00000120733	PAR-CLIP
JMJD1C	ENSG00000171988	HITS-CLIP
TIMP3	ENSG00000100234	HITS-CLIP
STAU2	ENSG00000040341	PAR-CLIP
H2AFZ	ENSG00000164032	HITS-CLIP
GNG2	ENSG00000186469	HITS-CLIP
ATP11A	ENSG00000068650	PAR-CLIP
HIATL1	ENSG00000148110	PAR-CLIP
CREBZF	ENSG00000137504	PAR-CLIP
SFPQ	ENSG00000116560	Multiple
DAG1	ENSG00000173402	Multiple
RPRD1B	ENSG00000101413	HITS-CLIP
CFL2	ENSG00000165410	PAR-CLIP
SEC24A	ENSG00000113615	Multiple
TEAD1	ENSG00000187079	Multiple
SCYL2	ENSG00000136021	HITS-CLIP
BAZ2A	ENSG00000076108	Multiple
STRN3	ENSG00000196792	PAR-CLIP

TGIF1	ENSG00000177426	HITS-CLIP
MOB4	ENSG00000115540	Multiple
STARD3NL	ENSG00000010270	PAR-CLIP
FKBP8	ENSG00000105701	HITS-CLIP
MARCKSL1	ENSG00000175130	Multiple
POMT1	ENSG00000130714	HITS-CLIP
CPEB2	ENSG00000137449	Multiple
RORA	ENSG00000069667	Multiple
SLC22A23	ENSG00000137266	HITS-CLIP
CDC42SE2	ENSG00000158985	Multiple
ZFAND6	ENSG00000086666	HITS-CLIP
C6ORF174	ENSG00000255330	PAR-CLIP
GLCCI1	ENSG00000106415	HITS-CLIP
HDAC2	ENSG00000196591	PAR-CLIP
NRP1	ENSG00000099250	Multiple
NAA30	ENSG00000139977	HITS-CLIP
RFX3	ENSG00000080298	HITS-CLIP
MBD6	ENSG00000166987	HITS-CLIP
TST	ENSG00000128311	PAR-CLIP
ARL5A	ENSG00000162980	HITS-CLIP
PSMB2	ENSG00000126067	HITS-CLIP
DHTKD1	ENSG00000181192	Multiple
RAP2C	ENSG00000123728	Multiple
TMPO	ENSG00000120802	PAR-CLIP
PRIM1	ENSG00000198056	HITS-CLIP
DCUN1D3	ENSG00000188215	HITS-CLIP
JAZF1	ENSG00000153814	HITS-CLIP
GNPNAT1	ENSG00000100522	HITS-CLIP
ASCC3	ENSG00000112249	HITS-CLIP
LARP4	ENSG00000161813	HITS-CLIP
CUX1	ENSG00000257923	HITS-CLIP
PTPRJ	ENSG00000149177	HITS-CLIP
TNFRSF21	ENSG00000146072	PAR-CLIP
ZBTB43	ENSG00000169155	Multiple
MNX1	ENSG00000130675	Multiple
RNASE4	ENSG00000258818	PAR-CLIP
RAC1	ENSG00000136238	PAR-CLIP
CHRAC1	ENSG00000104472	PAR-CLIP
C6orf62	ENSG00000112308	HITS-CLIP
OTUD4	ENSG00000164164	Multiple
STC2	ENSG00000113739	HITS-CLIP
MAK16	ENSG00000198042	PAR-CLIP
PEBP1	ENSG00000089220	HITS-CLIP
MESDC2	ENSG00000117899	HITS-CLIP

FGF2	ENSG00000138685	PAR-CLIP
ATP6V1A	ENSG00000114573	Multiple
RNF219	ENSG00000152193	Multiple
THAP2	ENSG00000173451	PAR-CLIP
KDM6B	ENSG00000132510	Multiple
MCUR1	ENSG00000050393	HITS-CLIP
ZFP64	ENSG00000020256	Multiple
HOXC13	ENSG00000123364	HITS-CLIP
SESN2	ENSG00000130766	Multiple
SDE2	ENSG00000143751	HITS-CLIP
HSPA4L	ENSG00000164070	Multiple
EBAG9	ENSG00000147654	HITS-CLIP
PSMD1	ENSG00000173692	HITS-CLIP
SURF4	ENSG00000148248	HITS-CLIP
C16orf72	ENSG00000182831	Multiple
MRGBP	ENSG00000101189	Multiple
PYGO2	ENSG00000163348	Multiple
SSX2IP	ENSG00000117155	PAR-CLIP
ZBTB21	ENSG00000173276	Multiple
DHX15	ENSG00000109606	PAR-CLIP
EMP2	ENSG00000213853	HITS-CLIP
DYNLL2	ENSG00000264364	HITS-CLIP
ZC3H11A	ENSG00000058673	PAR-CLIP
ADNP	ENSG00000101126	Multiple
MAD2L1	ENSG00000164109	Multiple
PSEN1	ENSG00000080815	Multiple
CCNY	ENSG00000108100	Multiple
ZNF430	ENSG00000118620	PAR-CLIP
TPI1	ENSG00000111669	Multiple
EIF2S1	ENSG00000134001	PAR-CLIP
RRM1	ENSG00000167325	HITS-CLIP
RANBP2	ENSG00000153201	HITS-CLIP
MGRN1	ENSG00000102858	HITS-CLIP
POLR3K	ENSG00000161980	PAR-CLIP
SP1	ENSG00000185591	PAR-CLIP
RARS	ENSG00000113643	PAR-CLIP
OVGP1	ENSG00000085465	PAR-CLIP
FAM98A	ENSG00000119812	HITS-CLIP
PSMB1	ENSG00000008018	PAR-CLIP
ACTN4	ENSG00000130402	Multiple
KRT80	ENSG00000167767	Multiple
FNIP1	ENSG00000217128	HITS-CLIP
SLC39A6	ENSG00000141424	HITS-CLIP
FUT11	ENSG00000196968	PAR-CLIP

SMIM13	ENSG00000224531	Multiple
KLC1	ENSG00000126214	PAR-CLIP
NPTN	ENSG00000156642	HITS-CLIP
PTPLAD1	ENSG00000074696	PAR-CLIP
ZNF451	ENSG00000112200	Multiple
GNPTAB	ENSG00000111670	Multiple
TSPYL4	ENSG00000187189	PAR-CLIP
NFE2L2	ENSG00000116044	Multiple
EPC2	ENSG00000135999	PAR-CLIP
FAS	ENSG00000026103	Multiple
ARID4B	ENSG00000054267	HITS-CLIP
PRICKLE2	ENSG00000163637	HITS-CLIP
FOXF1	ENSG00000103241	Multiple
RPS6KA3	ENSG00000177189	HITS-CLIP
MKI67	ENSG00000148773	PAR-CLIP
SCOC	ENSG00000153130	HITS-CLIP
FXR2	ENSG00000129245	PAR-CLIP
TGFBR3	ENSG00000069702	Multiple
UBE2F	ENSG00000184182	PAR-CLIP
SUMO3	ENSG00000184900	HITS-CLIP
TLK1	ENSG00000198586	HITS-CLIP
CD200R1	ENSG00000163606	PAR-CLIP
EP300	ENSG00000100393	HITS-CLIP
POM121	ENSG00000196313	HITS-CLIP
PRDM2	ENSG00000116731	PAR-CLIP
ZNF714	ENSG00000160352	Multiple
DDAH1	ENSG00000153904	HITS-CLIP
GAB1	ENSG00000109458	HITS-CLIP
PGRMC2	ENSG00000164040	PAR-CLIP
MPRIP	ENSG00000133030	HITS-CLIP
ACP2	ENSG00000134575	HITS-CLIP
TFB2M	ENSG00000162851	Multiple
SLC44A1	ENSG00000070214	PAR-CLIP
DCUN1D1	ENSG00000043093	Multiple
CHSY1	ENSG00000131873	PAR-CLIP
GPCPD1	ENSG00000125772	HITS-CLIP
CEBPD	ENSG00000221869	HITS-CLIP
FAM73A	ENSG00000180488	Multiple
RASSF9	ENSG00000198774	Multiple
CDK17	ENSG00000059758	Multiple
ZBTB7A	ENSG00000178951	PAR-CLIP
PLAG1	ENSG00000181690	PAR-CLIP
PUM2	ENSG00000055917	HITS-CLIP
NFIB	ENSG00000147862	Multiple

REV3L	ENSG00000009413	Multiple
AKT3	ENSG00000117020	PAR-CLIP
CLDN4	ENSG00000189143	HITS-CLIP
GLTSCR1	ENSG00000063169	Multiple
PITPNB	ENSG00000180957	Multiple
STON2	ENSG00000140022	HITS-CLIP
ATF7IP	ENSG00000171681	Multiple
HPRT1	ENSG00000165704	HITS-CLIP
OSGIN2	ENSG00000164823	HITS-CLIP
CHMP3	ENSG00000115561	HITS-CLIP
HNRNPH2	ENSG00000126945	HITS-CLIP
TSPYL1	ENSG00000189241	Multiple
SCAF11	ENSG00000139218	HITS-CLIP
POLR1E	ENSG00000137054	PAR-CLIP
MPHOSPH9	ENSG00000051825	PAR-CLIP
UBXN1	ENSG00000162191	HITS-CLIP
STAMBP	ENSG00000124356	HITS-CLIP
UBE2D1	ENSG00000072401	Multiple
G3BP2	ENSG00000138757	HITS-CLIP
RBBP7	ENSG00000102054	Multiple
PABPN1	ENSG00000100836	PAR-CLIP
PCDH7	ENSG00000169851	HITS-CLIP
MFF	ENSG00000168958	PAR-CLIP
AHCY	ENSG00000101444	HITS-CLIP
ARCN1	ENSG00000095139	Multiple
KPNA6	ENSG00000025800	HITS-CLIP
EIF3M	ENSG00000149100	HITS-CLIP
NCBP2	ENSG00000114503	Multiple
LONRF2	ENSG00000170500	PAR-CLIP
SPINT1	ENSG00000166145	HITS-CLIP
CYP1B1	ENSG00000138061	HITS-CLIP
ACSL4	ENSG00000068366	PAR-CLIP
UBE2D2	ENSG00000131508	Multiple
UBE2G1	ENSG00000132388	Multiple
SDC2	ENSG00000169439	HITS-CLIP
TFIP11	ENSG00000100109	HITS-CLIP
PAPD7	ENSG00000112941	HITS-CLIP
RLIM	ENSG00000131263	HITS-CLIP
ZNF512B	ENSG00000196700	HITS-CLIP
C8orf76	ENSG00000189376	Multiple
PLEKHA1	ENSG00000107679	Multiple
ZNRF2	ENSG00000180233	PAR-CLIP
ZNF585A	ENSG00000196967	PAR-CLIP
METTL14	ENSG00000145388	Multiple

NDC1	ENSG00000058804	HITS-CLIP
SLC7A2	ENSG00000003989	Multiple
TULP4	ENSG00000130338	PAR-CLIP
FMN2	ENSG00000155816	Multiple
MYCN	ENSG00000134323	Multiple
2-Sep	ENSG00000168385	Multiple
DLX1	ENSG00000144355	PAR-CLIP
C22orf39	ENSG00000242259	HITS-CLIP
GFRA1	ENSG00000151892	Multiple
DIMT1	ENSG00000086189	Multiple
PKN2	ENSG00000065243	HITS-CLIP
TMEM126B	ENSG00000171204	HITS-CLIP
USP7	ENSG00000187555	HITS-CLIP
APOOL	ENSG00000155008	Multiple
MATR3	ENSG00000015479	PAR-CLIP
LRIF1	ENSG00000121931	Multiple
TMEM30A	ENSG00000112697	HITS-CLIP
CDKN1A	ENSG00000124762	Multiple
ERH	ENSG00000100632	HITS-CLIP
STMN1	ENSG00000117632	Multiple
UHMK1	ENSG00000152332	Multiple
WTAP	ENSG00000146457	Multiple
BDP1	ENSG00000145734	HITS-CLIP
CCNJ	ENSG00000107443	PAR-CLIP
C3orf38	ENSG00000179021	Multiple
CLSPN	ENSG00000092853	PAR-CLIP
HIAT1	ENSG00000156875	PAR-CLIP
TNRC6A	ENSG00000090905	Multiple
MAP3K2	ENSG00000169967	PAR-CLIP
ARF6	ENSG00000165527	PAR-CLIP
USP9X	ENSG00000124486	Multiple
MBNL1	ENSG00000152601	Multiple
PRC1	ENSG00000198901	PAR-CLIP
ZFYVE21	ENSG00000100711	HITS-CLIP
HEXIM1	ENSG00000186834	PAR-CLIP
TMEM201	ENSG00000188807	PAR-CLIP
MON2	ENSG00000061987	HITS-CLIP
PDXDC1	ENSG00000179889	PAR-CLIP
NDUFA2	ENSG00000131495	Multiple
ZFHX4	ENSG00000091656	Multiple
OXR1	ENSG00000164830	PAR-CLIP
CNEP1R1	ENSG00000205423	PAR-CLIP
TNFAIP1	ENSG00000109079	PAR-CLIP
ZBTB18	ENSG00000179456	PAR-CLIP

GFPT2	ENSG00000131459	HITS-CLIP
INA	ENSG00000148798	PAR-CLIP
SUV39H2	ENSG00000152455	PAR-CLIP
SLC39A10	ENSG00000196950	HITS-CLIP
CKAP2	ENSG00000136108	PAR-CLIP
TGIF2	ENSG00000118707	PAR-CLIP
PTX3	ENSG00000163661	HITS-CLIP
OTULIN	ENSG00000154124	HITS-CLIP
HNRNPA2B1	ENSG00000122566	Multiple
DCAF12	ENSG00000198876	HITS-CLIP
RPRD1A	ENSG00000141425	Multiple
MOB3C	ENSG00000142961	HITS-CLIP
ACYP1	ENSG00000119640	PAR-CLIP
ZBTB1	ENSG00000126804	PAR-CLIP
CCDC186	ENSG00000165813	HITS-CLIP
MORC3	ENSG00000159256	Multiple
BCL11B	ENSG00000127152	PAR-CLIP
GORASP2	ENSG00000115806	HITS-CLIP
AASDHPPT	ENSG00000149313	Multiple
BCOR	ENSG00000183337	PAR-CLIP
DDX1	ENSG00000079785	HITS-CLIP
SHANK2	ENSG00000162105	HITS-CLIP
ZFP41	ENSG00000181638	PAR-CLIP
CCNI	ENSG00000118816	Multiple
BTBD7	ENSG00000011114	Multiple
PHF13	ENSG00000116273	Multiple
CKS2	ENSG00000123975	HITS-CLIP
HIPK1	ENSG00000163349	Multiple
HIST2H2BE	ENSG00000184678	Multiple
NRIP1	ENSG00000180530	HITS-CLIP
ZBTB34	ENSG00000177125	Multiple
RGL1	ENSG00000143344	Multiple
AP1G1	ENSG00000166747	Multiple
SMAD7	ENSG00000101665	HITS-CLIP
RAB8B	ENSG00000166128	PAR-CLIP
CAAP1	ENSG00000120159	PAR-CLIP
POMP	ENSG00000132963	HITS-CLIP
DCLRE1B	ENSG00000118655	Multiple
ZNF367	ENSG00000165244	Multiple
TRIQK	ENSG00000205133	PAR-CLIP
TSN	ENSG00000211460	Multiple
ZFP36L2	ENSG00000152518	Multiple
TMEM192	ENSG00000170088	PAR-CLIP
NUPL2	ENSG00000136243	PAR-CLIP

KHNYN	ENSG00000100441	PAR-CLIP
ABCC5	ENSG00000114770	PAR-CLIP
NKRF	ENSG00000186416	HITS-CLIP
MAGT1	ENSG00000102158	HITS-CLIP
GATAD1	ENSG00000157259	PAR-CLIP
CMTM6	ENSG00000091317	Multiple
ABHD17C	ENSG00000136379	PAR-CLIP
NR1D2	ENSG00000174738	Multiple
SGOL2	ENSG00000163535	HITS-CLIP
RIPK4	ENSG00000183421	HITS-CLIP
SRSF10	ENSG00000188529	PAR-CLIP
EXOC8	ENSG00000116903	PAR-CLIP
METTL3	ENSG00000165819	PAR-CLIP
GPR27	ENSG00000170837	HITS-CLIP
PDZD8	ENSG00000165650	HITS-CLIP
CLSTN1	ENSG00000171603	HITS-CLIP
XRCC5	ENSG00000079246	HITS-CLIP
KMT2E	ENSG00000005483	Multiple
USP3	ENSG00000140455	HITS-CLIP
YAE1D1	ENSG00000241127	PAR-CLIP
PPARGC1B	ENSG00000155846	HITS-CLIP
RAB11FIP1	ENSG00000156675	Multiple
CCNC	ENSG00000112237	HITS-CLIP
ZNF618	ENSG00000157657	PAR-CLIP
GTF2A1	ENSG00000165417	HITS-CLIP
TANC2	ENSG00000170921	HITS-CLIP
TRIM71	ENSG00000206557	PAR-CLIP
PHIP	ENSG00000146247	Multiple
CPEB3	ENSG00000107864	PAR-CLIP
ZC3H4	ENSG00000130749	PAR-CLIP
MTF2	ENSG00000143033	HITS-CLIP
HNRNPUL1	ENSG00000105323	Multiple
MYH6	ENSG00000197616	PAR-CLIP
TRIM25	ENSG00000121060	PAR-CLIP
CD55	ENSG00000196352	HITS-CLIP
TMEM55A	ENSG00000155099	HITS-CLIP
RNF213	ENSG00000173821	Multiple
BBX	ENSG00000114439	PAR-CLIP
OSMR	ENSG00000145623	HITS-CLIP
CSRP2	ENSG00000175183	HITS-CLIP
TRIB1	ENSG00000173334	Multiple
MAPK1	ENSG00000100030	Multiple
MAP3K7	ENSG00000135341	Multiple
FNDC3A	ENSG00000102531	HITS-CLIP

SLAIN2	ENSG00000109171	PAR-CLIP
NUP205	ENSG00000155561	Multiple
FGF1	ENSG00000113578	HITS-CLIP
NUS1	ENSG00000153989	Multiple
NECAB1	ENSG00000123119	HITS-CLIP
C5orf15	ENSG00000113583	PAR-CLIP
MAPK6	ENSG00000069956	Multiple
LATS2	ENSG00000150457	PAR-CLIP
LNX2	ENSG00000139517	HITS-CLIP
MED6	ENSG00000133997	Multiple
PTGFRN	ENSG00000134247	HITS-CLIP
FBXW11	ENSG00000072803	HITS-CLIP
ZNF146	ENSG00000167635	PAR-CLIP
CSF1	ENSG00000184371	PAR-CLIP
RANBP9	ENSG00000010017	Multiple
AP3M1	ENSG00000185009	PAR-CLIP
GULP1	ENSG00000144366	HITS-CLIP
SMCO4	ENSG00000166002	HITS-CLIP
KMT2C	ENSG00000055609	Multiple
TBPL1	ENSG00000028839	HITS-CLIP
MGAT4A	ENSG00000071073	Multiple
HELLS	ENSG00000119969	PAR-CLIP
GCNT1	ENSG00000187210	PAR-CLIP
HOXA10	ENSG00000253293	HITS-CLIP
C7orf31	ENSG00000153790	Multiple
PPM1D	ENSG00000170836	HITS-CLIP
USP22	ENSG00000124422	Multiple
ZSCAN9	ENSG00000137185	PAR-CLIP
PATL1	ENSG00000166889	PAR-CLIP
CPNE8	ENSG00000139117	PAR-CLIP
SLC23A2	ENSG00000089057	Multiple
LYSMD3	ENSG00000176018	PAR-CLIP
DIS3	ENSG00000083520	PAR-CLIP
TAF3	ENSG00000165632	PAR-CLIP
COL5A2	ENSG00000204262	HITS-CLIP
C17orf104	ENSG00000180336	PAR-CLIP
GNB4	ENSG00000114450	PAR-CLIP
RANBP6	ENSG00000137040	HITS-CLIP
SLMO2	ENSG00000101166	HITS-CLIP
ZNF623	ENSG00000183309	Multiple
TOP1	ENSG00000198900	PAR-CLIP
ZNF675	ENSG00000197372	HITS-CLIP
RNF139	ENSG00000170881	HITS-CLIP
CCDC113	ENSG00000103021	Multiple

STARD4	ENSG00000164211	PAR-CLIP
TNKS	ENSG00000173273	Multiple
TBL1XR1	ENSG00000177565	HITS-CLIP
ERO1LB	ENSG00000086619	PAR-CLIP
SETX	ENSG00000107290	PAR-CLIP
SPOP	ENSG00000121067	PAR-CLIP
DUSP1	ENSG00000120129	Multiple
RNASE1	ENSG00000129538	HITS-CLIP
IREB2	ENSG00000136381	PAR-CLIP
BZW2	ENSG00000136261	HITS-CLIP
SECISBP2L	ENSG00000138593	PAR-CLIP
TGFBR2	ENSG00000163513	HITS-CLIP
SLC1A5	ENSG00000105281	PAR-CLIP
MEF2A	ENSG00000068305	Multiple
BICC1	ENSG00000122870	HITS-CLIP
HIVEP3	ENSG00000127124	PAR-CLIP
RAB14	ENSG00000119396	PAR-CLIP
IRF1	ENSG00000125347	PAR-CLIP
ADRB1	ENSG00000043591	Multiple
MTSS1L	ENSG00000132613	HITS-CLIP
PVRL1	ENSG00000110400	HITS-CLIP
RPL15	ENSG00000174748	PAR-CLIP
TSPAN13	ENSG00000106537	HITS-CLIP
NPC1L1	ENSG00000015520	HITS-CLIP
ARHGAP5	ENSG00000100852	PAR-CLIP
RAPH1	ENSG00000173166	Multiple
KHDRBS1	ENSG00000121774	HITS-CLIP
RPA2	ENSG00000117748	PAR-CLIP
USH2A	ENSG00000042781	PAR-CLIP
AKAP11	ENSG00000023516	Multiple
SP9	ENSG00000217236	PAR-CLIP
BAG5	ENSG00000166170	HITS-CLIP
SLC39A14	ENSG00000104635	HITS-CLIP
TOMM6	ENSG00000214736	Multiple
LPGAT1	ENSG00000123684	Multiple
PRR11	ENSG00000068489	HITS-CLIP
TNRC6C	ENSG00000078687	Multiple
RNF216	ENSG00000011275	Multiple
FAM110C	ENSG00000184731	PAR-CLIP
MEIS1	ENSG00000143995	Western Blot
KIAA1551	ENSG00000174718	PAR-CLIP
PNISR	ENSG00000132424	PAR-CLIP
TMBIM6	ENSG00000139644	PAR-CLIP
ARHGAP12	ENSG00000165322	PAR-CLIP

FLVCR1	ENSG00000162769	PAR-CLIP
EIF4G2	ENSG00000110321	Multiple
BCL2L11	ENSG00000153094	Multiple
GTF2E1	ENSG00000153767	PAR-CLIP
NR2F2	ENSG00000185551	PAR-CLIP
KIF5B	ENSG00000170759	Multiple
ZNF740	ENSG00000139651	PAR-CLIP
MYO1B	ENSG00000128641	PAR-CLIP
LIN7C	ENSG00000148943	Multiple
GNAI1	ENSG00000127955	HITS-CLIP
MLLT10	ENSG00000078403	HITS-CLIP
ARHGEF3	ENSG00000163947	HITS-CLIP
USP34	ENSG00000115464	PAR-CLIP
EFHD1	ENSG00000115468	PAR-CLIP
CD44	ENSG00000026508	PAR-CLIP
MAP1B	ENSG00000131711	PAR-CLIP
CD47	ENSG00000196776	PAR-CLIP
DHX33	ENSG00000005100	HITS-CLIP
SLC29A2	ENSG00000174669	HITS-CLIP
UBR4	ENSG00000127481	HITS-CLIP
ZKSCAN1	ENSG00000106261	HITS-CLIP
LIG4	ENSG00000174405	HITS-CLIP
PPP1CB	ENSG00000213639	HITS-CLIP
TSHZ2	ENSG00000182463	HITS-CLIP
SYNCRIP	ENSG00000135316	Multiple
MEX3C	ENSG00000176624	PAR-CLIP
YTHDC1	ENSG00000083896	PAR-CLIP
SCFD2	ENSG00000184178	HITS-CLIP
INO80D	ENSG00000114933	Multiple
PANK1	ENSG00000152782	PAR-CLIP
CUL4B	ENSG00000158290	PAR-CLIP
NDEL1	ENSG00000166579	Multiple
TUSC2	ENSG00000114383	Multiple
C7orf55-LUC7L2	ENSG00000146963	PAR-CLIP
TXNIP	ENSG00000265972	Multiple
PCDHB16	ENSG00000272674	HITS-CLIP
ZNF792	ENSG00000180884	Multiple
VAMP3	ENSG00000049245	PAR-CLIP
SYNRG	ENSG00000275066	HITS-CLIP
PNLIPRP2	ENSG00000266200	HITS-CLIP
ZFP91	ENSG00000186660	Multiple
CALU	ENSG00000128595	Multiple
EFNB2	ENSG00000125266	Multiple
CCNT2	ENSG00000082258	PAR-CLIP

RTN4	ENSG00000115310	Multiple
C17orf85	ENSG00000074356	HITS-CLIP
DDI2	ENSG00000197312	HITS-CLIP
RPA1	ENSG00000132383	PAR-CLIP
VPS13C	ENSG00000129003	HITS-CLIP
BMPER	ENSG00000164619	HITS-CLIP
RBM14	ENSG00000239306	HITS-CLIP
NSL1	ENSG00000117697	HITS-CLIP
POU2F1	ENSG00000143190	HITS-CLIP
SFT2D1	ENSG00000198818	HITS-CLIP
CDC6	ENSG00000094804	HITS-CLIP
BLZF1	ENSG00000117475	PAR-CLIP
DUSP4	ENSG00000120875	HITS-CLIP
C22orf46	ENSG00000184208	HITS-CLIP
TMEM50A	ENSG00000183726	Multiple
FOXJ2	ENSG00000065970	HITS-CLIP
SERTAD3	ENSG00000167565	HITS-CLIP
PPP1R37	ENSG00000104866	Multiple
PICALM	ENSG00000073921	HITS-CLIP
JARID2	ENSG00000008083	Multiple
MTA3	ENSG00000057935	HITS-CLIP
CTDSP1	ENSG00000144579	HITS-CLIP
FAM120A	ENSG00000048828	HITS-CLIP
SOX4	ENSG00000124766	Multiple
CDIP1	ENSG00000089486	HITS-CLIP
PRKCA	ENSG00000154229	HITS-CLIP
CAMSAP3	ENSG00000076826	HITS-CLIP
VWA9	ENSG00000138614	Multiple
TUBGCP3	ENSG00000126216	HITS-CLIP
SPARC	ENSG00000113140	HITS-CLIP
UGCG	ENSG00000148154	Multiple
B3GNT5	ENSG00000176597	HITS-CLIP
RREB1	ENSG00000124782	HITS-CLIP
PDCL3	ENSG00000115539	HITS-CLIP
CEBPG	ENSG00000153879	Multiple
SIGMAR1	ENSG00000147955	HITS-CLIP
EFCAB14	ENSG00000159658	HITS-CLIP
HS1BP3	ENSG00000118960	HITS-CLIP
NBL1	ENSG00000158747	HITS-CLIP
TBC1D13	ENSG00000107021	HITS-CLIP
CDH13	ENSG00000140945	HITS-CLIP
MTERF	ENSG00000127989	HITS-CLIP
NPDC1	ENSG00000107281	HITS-CLIP
ADAMTS4	ENSG00000158859	Multiple

SNX18	ENSG00000178996	Multiple
ADAMTS5	ENSG00000154736	Multiple
ERBB2	ENSG00000141736	Multiple
LACTB	ENSG00000103642	HITS-CLIP
FAM53B	ENSG00000189319	HITS-CLIP
IRF4	ENSG00000137265	HITS-CLIP
MANEA	ENSG00000172469	HITS-CLIP
OAZ1	ENSG00000104904	PAR-CLIP
RAP2B	ENSG00000181467	HITS-CLIP
ARID3A	ENSG00000116017	Multiple
FAM129B	ENSG00000136830	HITS-CLIP
SLC6A6	ENSG00000131389	HITS-CLIP
SIPA1L3	ENSG00000105738	HITS-CLIP
CNOT11	ENSG00000158435	HITS-CLIP
MEGF9	ENSG00000106780	Multiple
SSH2	ENSG00000141298	HITS-CLIP
GRB10	ENSG00000106070	PAR-CLIP
PHF20L1	ENSG00000129292	HITS-CLIP
TTL	ENSG00000114999	HITS-CLIP
BCL9	ENSG00000116128	HITS-CLIP
STOX2	ENSG00000173320	Multiple
TACC2	ENSG00000138162	HITS-CLIP
CDR2L	ENSG00000109089	Multiple
EZR	ENSG00000092820	HITS-CLIP
YAP1	ENSG00000137693	HITS-CLIP
HMGB3	ENSG00000029993	HITS-CLIP
SSR1	ENSG00000124783	HITS-CLIP
ELF2	ENSG00000109381	HITS-CLIP
AREL1	ENSG00000119682	HITS-CLIP
C1orf43	ENSG00000143612	HITS-CLIP
RPS28	ENSG00000233927	HITS-CLIP
AFMID	ENSG00000183077	HITS-CLIP
ALPK3	ENSG00000136383	HITS-CLIP
FUS	ENSG00000089280	Multiple
NELFCD	ENSG00000101158	HITS-CLIP
PGR	ENSG00000082175	HITS-CLIP
FAM126B	ENSG00000155744	HITS-CLIP
PWWP2A	ENSG00000170234	Multiple
TMED2	ENSG00000086598	PAR-CLIP
TOR2A	ENSG00000160404	Multiple
ALDH4A1	ENSG00000159423	HITS-CLIP
POLR2C	ENSG00000102978	PAR-CLIP
ZNF12	ENSG00000164631	HITS-CLIP
POU3F2	ENSG00000184486	PAR-CLIP

NXPE3	ENSG00000144815	PAR-CLIP
ACSS1	ENSG00000154930	HITS-CLIP
USP36	ENSG00000055483	HITS-CLIP
BCL3	ENSG00000069399	HITS-CLIP
AMFR	ENSG00000159461	HITS-CLIP
H2AFX	ENSG00000188486	Multiple
NLRP2	ENSG00000022556	HITS-CLIP
CRKL	ENSG00000099942	HITS-CLIP
ATXN2L	ENSG00000168488	HITS-CLIP
MTHFD2	ENSG00000065911	HITS-CLIP
CD59	ENSG00000085063	Multiple
PIWIL3	ENSG00000184571	PAR-CLIP
RASA2	ENSG00000155903	HITS-CLIP
ZNF775	ENSG00000196456	PAR-CLIP
GLDC	ENSG00000178445	HITS-CLIP
GTF3C5	ENSG00000148308	HITS-CLIP
MMS22L	ENSG00000146263	HITS-CLIP
KIF1B	ENSG00000054523	HITS-CLIP
TMED7-TICAM2	ENSG00000251201	HITS-CLIP
ZFYVE1	ENSG00000165861	Multiple
BTBD1	ENSG00000064726	Multiple
NT5C2	ENSG00000076685	HITS-CLIP
MBTPS1	ENSG00000140943	HITS-CLIP
WDR55	ENSG00000120314	PAR-CLIP
WARS	ENSG00000140105	Multiple
BRPF3	ENSG00000096070	Multiple
HMGCR	ENSG00000113161	HITS-CLIP
SLC25A12	ENSG00000115840	PAR-CLIP
SLC35G1	ENSG00000176273	PAR-CLIP
EFEMP1	ENSG00000115380	HITS-CLIP
HMGA2	ENSG00000149948	HITS-CLIP
TMEM101	ENSG00000091947	PAR-CLIP
C21orf58	ENSG00000160298	HITS-CLIP
AGO3	ENSG00000126070	PAR-CLIP
BTN3A3	ENSG00000111801	PAR-CLIP
PIP5K1C	ENSG00000186111	HITS-CLIP
KIAA1522	ENSG00000162522	Multiple
ETV3	ENSG00000117036	Multiple
ARMCX4	ENSG00000196440	HITS-CLIP
PRRG4	ENSG00000135378	PAR-CLIP
GLRB	ENSG00000109738	HITS-CLIP
LENG8	ENSG00000167615	Multiple
PAK2	ENSG00000180370	Multiple
TMEM248	ENSG00000106609	HITS-CLIP

TPR	ENSG00000047410	HITS-CLIP
WNT10B	ENSG00000169884	HITS-CLIP
MTUS1	ENSG00000129422	PAR-CLIP
REST	ENSG00000084093	Multiple
PPP2CA	ENSG00000113575	PAR-CLIP
CEP128	ENSG00000100629	HITS-CLIP
GTF3C3	ENSG00000119041	HITS-CLIP
BCL2L13	ENSG00000099968	HITS-CLIP
ADAR	ENSG00000160710	Multiple
COPS7B	ENSG00000144524	Multiple
PLAGL2	ENSG00000126003	Multiple
ZDBF2	ENSG00000204186	HITS-CLIP
AAED1	ENSG00000158122	Multiple
LRRC8A	ENSG00000136802	HITS-CLIP
NR6A1	ENSG00000148200	Multiple
YIPF6	ENSG00000181704	HITS-CLIP
SORT1	ENSG00000134243	HITS-CLIP
CHD8	ENSG00000100888	Multiple
KIF3A	ENSG00000131437	PAR-CLIP
IST1	ENSG00000182149	Multiple
11-Sep	ENSG00000138758	HITS-CLIP
OGT	ENSG00000147162	HITS-CLIP
C2CD2L	ENSG00000172375	PAR-CLIP
MAZ	ENSG00000103495	Multiple
ARHGEF12	ENSG00000196914	HITS-CLIP
SGPL1	ENSG00000166224	Multiple
SZRD1	ENSG00000055070	Multiple
PPP1CC	ENSG00000186298	PAR-CLIP
DUSP6	ENSG00000139318	Multiple
NUP133	ENSG00000069248	HITS-CLIP
MRPL34	ENSG00000130312	PAR-CLIP
CAPNS1	ENSG00000126247	Multiple
GDNF	ENSG00000168621	HITS-CLIP
ACTG1	ENSG00000184009	Multiple
FOXM1	ENSG00000111206	HITS-CLIP
ZNF260	ENSG00000254004	PAR-CLIP
SATB2	ENSG00000119042	PAR-CLIP
PXN	ENSG00000089159	HITS-CLIP
TCEAL8	ENSG00000180964	HITS-CLIP
NUP210	ENSG00000132182	Multiple
MAPKAP1	ENSG00000119487	HITS-CLIP
HERPUD1	ENSG00000051108	Multiple
ANPEP	ENSG00000166825	HITS-CLIP
SPRED1	ENSG00000166068	PAR-CLIP

SH3BP2	ENSG00000087266	PAR-CLIP
PTPN14	ENSG00000152104	Multiple
ZFC3H1	ENSG00000133858	HITS-CLIP
TMEM63A	ENSG00000196187	PAR-CLIP
PKM	ENSG00000067225	HITS-CLIP
SNRK	ENSG00000163788	Multiple
PCTP	ENSG00000141179	Multiple
NOP10	ENSG00000182117	HITS-CLIP
STX3	ENSG00000166900	Multiple
WDR45	ENSG00000196998	HITS-CLIP
TPCN2	ENSG00000162341	PAR-CLIP
LOXL2	ENSG00000134013	HITS-CLIP
PTPRD	ENSG00000153707	HITS-CLIP
NBEAL2	ENSG00000160796	HITS-CLIP
ZNF121	ENSG00000197961	HITS-CLIP
ANKRD50	ENSG00000151458	PAR-CLIP
CDCA8	ENSG00000134690	Multiple
STK11	ENSG00000118046	Multiple
GPR180	ENSG00000152749	HITS-CLIP
HIST1H4C	ENSG00000197061	Multiple
STARD8	ENSG00000130052	CLASH
WAC	ENSG00000095787	HITS-CLIP
IQGAP1	ENSG00000140575	HITS-CLIP
WNK1	ENSG00000060237	CLASH
MNT	ENSG00000070444	HITS-CLIP
RUFY3	ENSG00000018189	Multiple
PLA2G4F	ENSG00000168907	CLASH
GSE1	ENSG00000131149	PAR-CLIP
GPATCH2	ENSG00000092978	Multiple
HOXB5	ENSG00000120075	HITS-CLIP
DDX42	ENSG00000198231	PAR-CLIP
CA12	ENSG00000074410	Multiple
SPATA13	ENSG00000182957	HITS-CLIP
ZNF562	ENSG00000171466	HITS-CLIP
MGA	ENSG00000174197	HITS-CLIP
CDH11	ENSG00000140937	HITS-CLIP
CDCA4	ENSG00000170779	HITS-CLIP
FBXO45	ENSG00000174013	PAR-CLIP
SMEK1	ENSG00000100796	Multiple
TMEM138	ENSG00000149483	PAR-CLIP
GINS3	ENSG00000181938	PAR-CLIP
PKD1	ENSG00000008710	HITS-CLIP
NBN	ENSG00000104320	HITS-CLIP
VPS36	ENSG00000136100	Multiple

IER2	ENSG00000160888	PAR-CLIP
AKAP1	ENSG00000121057	PAR-CLIP
AFAP1	ENSG00000196526	HITS-CLIP
CPSF6	ENSG00000111605	HITS-CLIP
ARID3B	ENSG00000179361	Multiple
CDKN1B	ENSG00000111276	PAR-CLIP
MYH9	ENSG00000100345	HITS-CLIP
CUL1	ENSG00000055130	HITS-CLIP
RPS6KA1	ENSG00000117676	Multiple
DDX6	ENSG00000110367	PAR-CLIP
ANKIB1	ENSG00000001629	HITS-CLIP
MDFIC	ENSG00000135272	HITS-CLIP
AGO2	ENSG00000123908	Multiple
OTUB1	ENSG00000167770	HITS-CLIP
LIN28B	ENSG00000187772	PAR-CLIP
RAB2A	ENSG00000104388	PAR-CLIP
ZSCAN29	ENSG00000140265	Multiple
EPG5	ENSG00000152223	HITS-CLIP
CSDE1	ENSG00000009307	Multiple
AXIN1	ENSG00000103126	PAR-CLIP
LMNB1	ENSG00000113368	HITS-CLIP
CDKN2B	ENSG00000147883	HITS-CLIP
TIAM1	ENSG00000156299	PAR-CLIP
FBXL20	ENSG00000108306	HITS-CLIP
ATP1B1	ENSG00000143153	HITS-CLIP
YWHAB	ENSG00000166913	HITS-CLIP
TNFSF15	ENSG00000181634	HITS-CLIP
KLHL21	ENSG00000162413	HITS-CLIP
THRA	ENSG00000126351	HITS-CLIP
SIRT5	ENSG00000124523	HITS-CLIP
GON4L	ENSG00000116580	HITS-CLIP
DNAJC14	ENSG00000135392	Multiple
CNST	ENSG00000162852	Multiple
MINK1	ENSG00000141503	PAR-CLIP
DDX17	ENSG00000100201	Multiple
PABPC4	ENSG00000090621	CLASH
DOCK11	ENSG00000147251	HITS-CLIP
HIST4H4	ENSG00000197837	HITS-CLIP
P4HB	ENSG00000185624	Multiple
UGP2	ENSG00000169764	HITS-CLIP
NME2	ENSG00000011052	CLASH
ZFHX3	ENSG00000140836	Multiple
TPX2	ENSG00000088325	HITS-CLIP
ARF1	ENSG00000143761	PAR-CLIP

COX7A2L	ENSG00000115944	HITS-CLIP
CLTC	ENSG00000141367	HITS-CLIP
C6orf48	ENSG00000204387	HITS-CLIP
ERCC6	ENSG00000225830	HITS-CLIP
IKZF4	ENSG00000123411	HITS-CLIP
PTCH2	ENSG00000117425	PAR-CLIP
NDRG1	ENSG00000104419	HITS-CLIP
IKZF3	ENSG00000161405	HITS-CLIP
ZNF768	ENSG00000169957	HITS-CLIP
LRIG1	ENSG00000144749	HITS-CLIP
EVC	ENSG00000072840	HITS-CLIP
TAF6	ENSG00000106290	HITS-CLIP
KDM2A	ENSG00000173120	HITS-CLIP
KIAA0196	ENSG00000164961	PAR-CLIP
MTMR2	ENSG00000087053	HITS-CLIP
QSER1	ENSG00000060749	PAR-CLIP
HNRNPU	ENSG00000153187	HITS-CLIP
IER3IP1	ENSG00000134049	Multiple
ATP6V1G1	ENSG00000136888	HITS-CLIP
RNF19B	ENSG00000116514	HITS-CLIP
SWSAP1	ENSG00000173928	PAR-CLIP
SPTAN1	ENSG00000197694	HITS-CLIP
VPS37B	ENSG00000139722	PAR-CLIP
DDX3X	ENSG00000215301	Multiple
IGF2BP1	ENSG00000159217	Multiple
SLC35A4	ENSG00000176087	Multiple
ECI2	ENSG00000198721	HITS-CLIP
SERINC1	ENSG00000111897	PAR-CLIP
TNFRSF1B	ENSG00000028137	HITS-CLIP
STXBP3	ENSG00000116266	HITS-CLIP
MORF4L2	ENSG00000123562	Multiple
RAD51D	ENSG00000185379	HITS-CLIP
RSPH3	ENSG00000130363	HITS-CLIP
APP	ENSG00000142192	HITS-CLIP
EPAS1	ENSG00000116016	HITS-CLIP
7-Mar	ENSG00000136536	Multiple
TAOK1	ENSG00000160551	PAR-CLIP
ALG9	ENSG00000086848	Multiple
CAPZA1	ENSG00000116489	PAR-CLIP
SV2A	ENSG00000159164	Multiple
NSUN3	ENSG00000178694	HITS-CLIP
PDS5A	ENSG00000121892	HITS-CLIP
ADIPOR2	ENSG00000006831	HITS-CLIP
FAM60A	ENSG00000139146	HITS-CLIP

CAPRIN1	ENSG00000135387	HITS-CLIP
SLC3A2	ENSG00000168003	Multiple
FAT4	ENSG00000196159	HITS-CLIP
VPS13A	ENSG00000197969	HITS-CLIP
NCOA3	ENSG00000124151	HITS-CLIP
WDR5	ENSG00000196363	Multiple
LMTK3	ENSG00000142235	HITS-CLIP
DPM2	ENSG00000136908	HITS-CLIP
EIF3K	ENSG00000178982	HITS-CLIP
GIGYF1	ENSG00000146830	PAR-CLIP
YWHAQ	ENSG00000134308	HITS-CLIP
NCL	ENSG00000115053	PAR-CLIP
FAM222B	ENSG00000173065	PAR-CLIP
MPP5	ENSG00000072415	HITS-CLIP
RHOV	ENSG00000104140	HITS-CLIP
SLC38A9	ENSG00000177058	HITS-CLIP
C15orf39	ENSG00000167173	HITS-CLIP
MFHAS1	ENSG00000147324	Multiple
CNP	ENSG00000173786	HITS-CLIP
ZNF543	ENSG00000178229	Multiple
FAM178A	ENSG00000119906	HITS-CLIP
RPP14	ENSG00000163684	Multiple
SEC61A2	ENSG00000065665	HITS-CLIP
MBD3	ENSG00000071655	HITS-CLIP
PCSK7	ENSG00000160613	HITS-CLIP
VARS	ENSG00000204394	PAR-CLIP
HIF1AN	ENSG00000166135	Multiple
ADAM17	ENSG00000151694	Multiple
EEF1D	ENSG00000104529	Multiple
MED1	ENSG00000125686	Multiple
BCAT1	ENSG00000060982	HITS-CLIP
GRN	ENSG00000030582	Multiple
UGT1A1	ENSG00000242366	HITS-CLIP
AMD1	ENSG00000123505	HITS-CLIP
CREB1	ENSG00000118260	PAR-CLIP
SS18	ENSG00000141380	HITS-CLIP
BOD1L2	ENSG00000228075	HITS-CLIP
ISYNA1	ENSG00000105655	HITS-CLIP
PRR14L	ENSG00000183530	Multiple
CHPF2	ENSG00000033100	HITS-CLIP
KLF13	ENSG00000169926	Multiple
EDEM3	ENSG00000116406	HITS-CLIP
CD81	ENSG00000110651	HITS-CLIP
MTR	ENSG00000116984	HITS-CLIP

MLLT4	ENSG00000130396	Multiple
ATP6V1B2	ENSG00000147416	HITS-CLIP
SLC7A1	ENSG00000139514	Multiple
ARID2	ENSG00000189079	Multiple
SOGA1	ENSG00000149639	Multiple
FURIN	ENSG00000140564	HITS-CLIP
EIF2S3	ENSG00000130741	HITS-CLIP
ZBTB37	ENSG00000185278	PAR-CLIP
MLEC	ENSG00000110917	HITS-CLIP
ZNF385A	ENSG00000161642	Multiple
SH3BP4	ENSG00000130147	PAR-CLIP
LARP1	ENSG00000155506	Multiple
ITFG2	ENSG00000111203	HITS-CLIP
DMWD	ENSG00000185800	HITS-CLIP
PPL	ENSG00000118898	HITS-CLIP
TRAK2	ENSG00000115993	HITS-CLIP
CSRNP1	ENSG00000144655	HITS-CLIP
DUS1L	ENSG00000169718	Multiple
PMAIP1	ENSG00000141682	Multiple
POP7	ENSG00000172336	HITS-CLIP
RAB11FIP2	ENSG00000107560	PAR-CLIP
REPIN1	ENSG00000214022	Multiple
NUDT16L1	ENSG00000168101	Multiple
YTHDF2	ENSG00000198492	Multiple
MFSD9	ENSG00000135953	Multiple
PSMG3	ENSG00000157778	HITS-CLIP
RBM39	ENSG00000131051	HITS-CLIP
KLHDC10	ENSG00000128607	HITS-CLIP
LFNG	ENSG00000106003	Multiple
PLEKHG3	ENSG00000126822	HITS-CLIP
KCTD21	ENSG00000188997	PAR-CLIP
S1PR3	ENSG00000213694	HITS-CLIP
CACHD1	ENSG00000158966	HITS-CLIP
GNB2	ENSG00000172354	HITS-CLIP
EEF2	ENSG00000167658	HITS-CLIP
CCDC142	ENSG00000135637	PAR-CLIP
GANAB	ENSG00000089597	HITS-CLIP
RBM20	ENSG00000203867	PAR-CLIP
ZNF395	ENSG00000186918	HITS-CLIP
B4GALT1	ENSG00000086062	Multiple
STX16	ENSG00000124222	HITS-CLIP
HOMEZ	ENSG00000215271	PAR-CLIP
RRP1	ENSG00000160214	HITS-CLIP
JUN	ENSG00000177606	Multiple

RPS12	ENSG00000112306	CLASH
CRTAP	ENSG00000170275	HITS-CLIP
FBXO28	ENSG00000143756	HITS-CLIP
DCP2	ENSG00000172795	HITS-CLIP
FBN2	ENSG00000138829	HITS-CLIP
LRCH2	ENSG00000130224	HITS-CLIP
TXLNA	ENSG00000084652	Multiple
GOLGA8A	ENSG00000175265	HITS-CLIP
CTB-102L5.4	ENSG00000267748	HITS-CLIP
NCAPD2	ENSG00000010292	PAR-CLIP
URM1	ENSG00000167118	HITS-CLIP
PPARA	ENSG00000186951	HITS-CLIP
IVL	ENSG00000163207	Multiple
FMNL3	ENSG00000161791	CLASH
CTNNB1	ENSG00000168036	PAR-CLIP
ZNF805	ENSG00000204524	HITS-CLIP
UNK	ENSG00000132478	HITS-CLIP
CDCA3	ENSG00000111665	Multiple
ACTR3	ENSG00000115091	HITS-CLIP
CTNNA1	ENSG00000044115	HITS-CLIP
RRP8	ENSG00000132275	PAR-CLIP
AXIN2	ENSG00000168646	HITS-CLIP
TRIM65	ENSG00000141569	HITS-CLIP
GFPT1	ENSG00000198380	HITS-CLIP
PABPC3	ENSG00000151846	HITS-CLIP
EPOR	ENSG00000187266	Multiple
SEC24C	ENSG00000176986	HITS-CLIP
SBNO1	ENSG00000139697	HITS-CLIP
BHLHE40	ENSG00000134107	PAR-CLIP
NPEPPS	ENSG00000141279	PAR-CLIP
COL18A1	ENSG00000182871	HITS-CLIP
POGK	ENSG00000143157	HITS-CLIP
RAPGEF2	ENSG00000109756	PAR-CLIP
PRR14	ENSG00000156858	HITS-CLIP
SEC23B	ENSG00000101310	Multiple
NFATC2IP	ENSG00000176953	HITS-CLIP
RNPS1	ENSG00000205937	HITS-CLIP
IGFBP4	ENSG00000141753	HITS-CLIP
DYNC1LI2	ENSG00000135720	PAR-CLIP
IFRD2	ENSG00000214706	PAR-CLIP
E2F3	ENSG00000112242	Multiple
SMARCA5	ENSG00000153147	HITS-CLIP
TSPAN15	ENSG00000099282	HITS-CLIP
SACS	ENSG00000151835	HITS-CLIP

HIST1H2AG	ENSG00000196787	PAR-CLIP
GTF2IRD1	ENSG00000006704	HITS-CLIP
PLXNB3	ENSG00000198753	PAR-CLIP
ZC3H7B	ENSG00000100403	HITS-CLIP
RIT1	ENSG00000143622	HITS-CLIP
STS	ENSG00000101846	HITS-CLIP
KIDINS220	ENSG00000134313	HITS-CLIP
DESI2	ENSG00000121644	PAR-CLIP
DLC1	ENSG00000164741	HITS-CLIP
ACTR2	ENSG00000138071	PAR-CLIP
IRS4	ENSG00000133124	HITS-CLIP
FAM120AOS	ENSG00000188938	HITS-CLIP
QKI	ENSG00000112531	HITS-CLIP
UBE3C	ENSG00000009335	HITS-CLIP
CENPF	ENSG00000117724	PAR-CLIP
SON	ENSG00000159140	Multiple
ASPHD1	ENSG00000174939	HITS-CLIP
TMEM14C	ENSG00000111843	HITS-CLIP
TXNRD1	ENSG00000198431	HITS-CLIP
BCKDK	ENSG00000103507	HITS-CLIP
TP53INP1	ENSG00000164938	Multiple
ELMSAN1	ENSG00000156030	PAR-CLIP
LIFR	ENSG00000113594	HITS-CLIP
HOXA3	ENSG00000105997	Multiple
DIAPH1	ENSG00000131504	HITS-CLIP
ATXN1	ENSG00000124788	Multiple
EPS15	ENSG00000085832	HITS-CLIP
DENND5B	ENSG00000170456	PAR-CLIP
AGPS	ENSG00000018510	HITS-CLIP
STK35	ENSG00000125834	Multiple
ANKRD42	ENSG00000137494	HITS-CLIP
MYL9	ENSG00000101335	HITS-CLIP
ZNF460	ENSG00000197714	Multiple
BIRC6	ENSG00000115760	Multiple
SPAG9	ENSG00000008294	HITS-CLIP
KRT10	ENSG00000186395	qPCR
KRT5	ENSG00000186081	qPCR
FCHO2	ENSG00000157107	HITS-CLIP
KIAA1462	ENSG00000165757	HITS-CLIP
SET	ENSG00000119335	PAR-CLIP
PSMD14	ENSG00000115233	PAR-CLIP
SLC11A2	ENSG00000110911	HITS-CLIP
WASF2	ENSG00000158195	HITS-CLIP
FBXO10	ENSG00000147912	HITS-CLIP

GABARAPL1	ENSG00000139112	PAR-CLIP
MPDU1	ENSG00000129255	HITS-CLIP
EIF1B	ENSG00000114784	HITS-CLIP
ZC3H13	ENSG00000123200	HITS-CLIP
SHISA5	ENSG00000164054	HITS-CLIP
XYLT2	ENSG00000015532	Multiple
NOL4L	ENSG00000197183	HITS-CLIP
SIM1	ENSG00000112246	PAR-CLIP
COPZ1	ENSG00000111481	Multiple
CDYL2	ENSG00000166446	HITS-CLIP
FLNA	ENSG00000196924	HITS-CLIP
ELOVL5	ENSG00000012660	Multiple
HIATL1	ENSG00000148110	HITS-CLIP
SLC39A9	ENSG00000029364	Multiple
TIMM13	ENSG00000099800	HITS-CLIP
MUC1	ENSG00000185499	Multiple
CDC42BPA	ENSG00000143776	HITS-CLIP
NCOA2	ENSG00000140396	HITS-CLIP
TMEM177	ENSG00000144120	Multiple
HIST1H4H	ENSG00000158406	HITS-CLIP
KCMF1	ENSG00000176407	PAR-CLIP
ZFP62	ENSG00000196670	HITS-CLIP
GPRIN1	ENSG00000169258	HITS-CLIP
TEAD1	ENSG00000187079	HITS-CLIP
SLFN13	ENSG00000154760	HITS-CLIP
AEN	ENSG00000181026	PAR-CLIP
SMARCC1	ENSG00000173473	HITS-CLIP
CFLAR	ENSG00000003402	Multiple
FAM118A	ENSG00000100376	Multiple
GGCX	ENSG00000115486	HITS-CLIP
PHC2	ENSG00000134686	HITS-CLIP
FBXL19	ENSG00000099364	HITS-CLIP
NRP1	ENSG00000099250	HITS-CLIP
SEMA4C	ENSG00000168758	Multiple
ZNF280D	ENSG00000137871	PAR-CLIP
CHAC1	ENSG00000128965	HITS-CLIP
SRRM2	ENSG00000167978	HITS-CLIP
KLHL15	ENSG00000174010	PAR-CLIP
SENP1	ENSG00000079387	HITS-CLIP
EEF1A1	ENSG00000156508	PAR-CLIP
PLEKHM3	ENSG00000178385	PAR-CLIP
TMEM259	ENSG00000182087	HITS-CLIP
DDX5	ENSG00000108654	Multiple
EIF5B	ENSG00000158417	HITS-CLIP

LZTS2	ENSG00000107816	HITS-CLIP
INSR	ENSG00000171105	PAR-CLIP
FLG	ENSG00000143631	qPCR
ZSWIM4	ENSG00000132003	HITS-CLIP
STC2	ENSG00000113739	Multiple
PTP4A1	ENSG00000112245	Multiple
KMT2D	ENSG00000167548	Multiple
SEPN1	ENSG00000162430	HITS-CLIP
POP1	ENSG00000104356	HITS-CLIP
PEA15	ENSG00000162734	PAR-CLIP
SAE1	ENSG00000142230	HITS-CLIP
PPP1R9B	ENSG00000108819	HITS-CLIP
BMP2	ENSG00000125845	HITS-CLIP
YRDC	ENSG00000196449	Multiple
AKAP2	ENSG00000241978	CLASH
TNFRSF10B	ENSG00000120889	Multiple
KDELR1	ENSG00000105438	HITS-CLIP
FAM210B	ENSG00000124098	HITS-CLIP
PUS7	ENSG00000091127	HITS-CLIP
FAM199X	ENSG00000123575	CLASH
TMEM2	ENSG00000135048	Multiple
LYPLA2	ENSG00000011009	Multiple
BUB1B	ENSG00000156970	HITS-CLIP
DYNLL2	ENSG00000264364	PAR-CLIP
BCL2L12	ENSG00000126453	Multiple
PPP1R12C	ENSG00000125503	Multiple
LCLAT1	ENSG00000172954	HITS-CLIP
FIGN	ENSG00000182263	HITS-CLIP
STRN	ENSG00000115808	Multiple
SMARCD2	ENSG00000108604	HITS-CLIP
CDK13	ENSG00000065883	HITS-CLIP
ZNF398	ENSG00000197024	PAR-CLIP
SDK2	ENSG00000069188	HITS-CLIP
DSG2	ENSG00000046604	Multiple
YY1	ENSG00000100811	PAR-CLIP
SLC16A6	ENSG00000108932	HITS-CLIP
SP1	ENSG00000185591	Multiple
MCRS1	ENSG00000187778	HITS-CLIP
MFSD5	ENSG00000182544	HITS-CLIP
SEMA4B	ENSG00000185033	HITS-CLIP
PSMB1	ENSG00000008018	Multiple
B3GALTL	ENSG00000187676	HITS-CLIP
KRT80	ENSG00000167767	HITS-CLIP
SMIM13	ENSG00000224531	HITS-CLIP

GTPBP2	ENSG00000172432	Multiple
AKIRIN1	ENSG00000174574	Multiple
UCHL3	ENSG00000118939	Multiple
MAX	ENSG00000125952	HITS-CLIP
GCSAM	ENSG00000174500	PAR-CLIP
RASSF3	ENSG00000153179	Multiple
TNKS1BP1	ENSG00000149115	Multiple
GFM1	ENSG00000168827	HITS-CLIP
STX6	ENSG00000135823	HITS-CLIP
PEX13	ENSG00000162928	HITS-CLIP
NFE2L1	ENSG00000082641	HITS-CLIP
TSPAN14	ENSG00000108219	Multiple
KLC2	ENSG00000174996	HITS-CLIP
DGKB	ENSG00000136267	HITS-CLIP
RCE1	ENSG00000173653	HITS-CLIP
SUN1	ENSG00000164828	PAR-CLIP
BBC3	ENSG00000105327	Multiple
ZNF35	ENSG00000169981	HITS-CLIP
MGEA5	ENSG00000198408	HITS-CLIP
ZBTB7A	ENSG00000178951	HITS-CLIP
THUMPD1	ENSG00000066654	PAR-CLIP
PARP12	ENSG00000059378	PAR-CLIP
TWISTNB	ENSG00000105849	PAR-CLIP
CLN6	ENSG00000128973	HITS-CLIP
C18orf54	ENSG00000166845	PAR-CLIP
CDK12	ENSG00000167258	HITS-CLIP
POMK	ENSG00000185900	HITS-CLIP
CNOT1	ENSG00000125107	Multiple
ZNF281	ENSG00000162702	Multiple
FNDC4	ENSG00000115226	HITS-CLIP
WHSC1	ENSG00000109685	HITS-CLIP
STARD13	ENSG00000133121	HITS-CLIP
INO80	ENSG00000128908	PAR-CLIP
COX10	ENSG00000006695	HITS-CLIP
CREB3L2	ENSG00000182158	Multiple
SCAF11	ENSG00000139218	Multiple
PARP1	ENSG00000143799	Multiple
SEC24B	ENSG00000138802	Multiple
CD4	ENSG00000010610	HITS-CLIP
RNF167	ENSG00000108523	Multiple
BRPF1	ENSG00000156983	Multiple
SUZ12	ENSG00000178691	PAR-CLIP
ARCN1	ENSG00000095139	PAR-CLIP
PDGFD	ENSG00000170962	HITS-CLIP

RC3H1	ENSG00000135870	Multiple
BSG	ENSG00000172270	HITS-CLIP
CDC27	ENSG00000004897	PAR-CLIP
SEC23IP	ENSG00000107651	Multiple
CAPN15	ENSG00000103326	HITS-CLIP
GPSM2	ENSG00000121957	HITS-CLIP
SCD	ENSG00000099194	HITS-CLIP
SUV420H2	ENSG00000133247	Multiple
EDC3	ENSG00000179151	HITS-CLIP
KIF18B	ENSG00000186185	PAR-CLIP
IL6ST	ENSG00000134352	HITS-CLIP
KDM7A	ENSG00000006459	HITS-CLIP
PLEKHA1	ENSG00000107679	HITS-CLIP
FAM102A	ENSG00000167106	HITS-CLIP
ZNF704	ENSG00000164684	HITS-CLIP
KIAA0368	ENSG00000136813	HITS-CLIP
PHF16	ENSG00000102221	PAR-CLIP
UBAP2L	ENSG00000143569	HITS-CLIP
ZBTB48	ENSG00000204859	HITS-CLIP
VWA5A	ENSG00000110002	HITS-CLIP
CHD5	ENSG00000116254	HITS-CLIP
UBN1	ENSG00000118900	HITS-CLIP
SEC22C	ENSG00000093183	PAR-CLIP
IGSF3	ENSG00000143061	HITS-CLIP
VPS37C	ENSG00000167987	HITS-CLIP
DDR1	ENSG00000204580	HITS-CLIP
DHX9	ENSG00000135829	HITS-CLIP
TIMM44	ENSG00000104980	HITS-CLIP
STARD7	ENSG00000084090	HITS-CLIP
UBN2	ENSG00000157741	PAR-CLIP
GATAD2B	ENSG00000143614	Multiple
ABHD3	ENSG00000158201	HITS-CLIP
EPS15L1	ENSG00000127527	HITS-CLIP
COBL	ENSG00000106078	HITS-CLIP
LRIF1	ENSG00000121931	HITS-CLIP
COG3	ENSG00000136152	HITS-CLIP
PPAN-P2RY11	ENSG00000243207	HITS-CLIP
RBM12B	ENSG00000183808	HITS-CLIP
SUPV3L1	ENSG00000156502	HITS-CLIP
ZBTB14	ENSG00000198081	HITS-CLIP
SLC25A19	ENSG00000125454	HITS-CLIP
TNFRSF12A	ENSG00000006327	HITS-CLIP
ARF6	ENSG00000165527	HITS-CLIP
TMEM201	ENSG00000188807	HITS-CLIP

PDXDC1	ENSG00000179889	HITS-CLIP
EIF6	ENSG00000242372	HITS-CLIP
CSPG4	ENSG00000173546	PAR-CLIP
PLD3	ENSG00000105223	PAR-CLIP
CCDC80	ENSG00000091986	Multiple
CHAMP1	ENSG00000198824	HITS-CLIP
MON1B	ENSG00000103111	HITS-CLIP
CARM1	ENSG00000142453	Multiple
EIF4EBP2	ENSG00000148730	Multiple
PDZD11	ENSG00000120509	Multiple
LDLR	ENSG00000130164	PAR-CLIP
EPO	ENSG00000130427	Multiple
EIF1AD	ENSG00000175376	HITS-CLIP
POLG	ENSG00000140521	PAR-CLIP
PER2	ENSG00000132326	Multiple
NUP155	ENSG00000113569	HITS-CLIP
ZNF483	ENSG00000173258	HITS-CLIP
LRP3	ENSG00000130881	HITS-CLIP
ADARB1	ENSG00000197381	HITS-CLIP
TNPO2	ENSG00000105576	Multiple
EMD	ENSG00000102119	HITS-CLIP
ATP2A2	ENSG00000174437	HITS-CLIP
AHNAK	ENSG00000124942	Multiple
YTHDF3	ENSG00000185728	Multiple
SH2B1	ENSG00000178188	HITS-CLIP
DGKD	ENSG00000077044	HITS-CLIP
SMAD7	ENSG00000101665	Multiple
UBE2W	ENSG00000104343	HITS-CLIP
NDUFS7	ENSG00000115286	PAR-CLIP
MKNK2	ENSG00000099875	Multiple
PPIF	ENSG00000108179	Multiple
ADD1	ENSG00000087274	PAR-CLIP
OSBPL8	ENSG00000091039	HITS-CLIP
PLEKHA6	ENSG00000143850	HITS-CLIP
AURKB	ENSG00000178999	CLASH
ZNF106	ENSG00000103994	HITS-CLIP
FAM134A	ENSG00000144567	Multiple
GPR157	ENSG00000180758	HITS-CLIP
KHNYN	ENSG00000100441	Multiple
MOAP1	ENSG00000165943	HITS-CLIP
FAM65A	ENSG00000039523	HITS-CLIP
LEPROT	ENSG00000213625	HITS-CLIP
PTAR1	ENSG00000188647	PAR-CLIP
LAMP1	ENSG00000185896	HITS-CLIP

FGFR2	ENSG00000066468	Multiple
MOB1A	ENSG00000114978	Multiple
PRDM4	ENSG00000110851	PAR-CLIP
VCPIP1	ENSG00000175073	HITS-CLIP
GFOD2	ENSG00000141098	PAR-CLIP
ACIN1	ENSG00000100813	HITS-CLIP
TTC9C	ENSG00000162222	HITS-CLIP
SNX5	ENSG00000089006	Multiple
EHD1	ENSG00000110047	Multiple
C10orf76	ENSG00000120029	HITS-CLIP
LSM14B	ENSG00000149657	PAR-CLIP
NME1	ENSG00000239672	HITS-CLIP
XPO1	ENSG00000082898	Multiple
EPHA4	ENSG00000116106	PAR-CLIP
UNC13B	ENSG00000198722	HITS-CLIP
PHIP	ENSG00000146247	HITS-CLIP
SCNN1A	ENSG00000111319	HITS-CLIP
VEGFA	ENSG00000112715	Multiple
PCF11	ENSG00000165494	PAR-CLIP
NFATC3	ENSG00000072736	HITS-CLIP
FGFR1	ENSG00000077782	HITS-CLIP
HNRNPUL1	ENSG00000105323	PAR-CLIP
ATL3	ENSG00000184743	HITS-CLIP
FAM160A2	ENSG00000051009	HITS-CLIP
AP1B1	ENSG00000100280	HITS-CLIP
IRAK1	ENSG00000184216	Multiple
DAAM1	ENSG00000100592	HITS-CLIP
RNF213	ENSG00000173821	HITS-CLIP
CTPS1	ENSG00000171793	HITS-CLIP
MUM1	ENSG00000160953	PAR-CLIP
EXOC7	ENSG00000182473	HITS-CLIP
DYNC2H1	ENSG00000187240	HITS-CLIP
TRIB1	ENSG00000173334	PAR-CLIP
NSFL1C	ENSG00000088833	HITS-CLIP
NIPBL	ENSG00000164190	HITS-CLIP
DNTTIP2	ENSG00000067334	Multiple
HIST1H2AC	ENSG00000180573	Multiple
SRF	ENSG00000112658	HITS-CLIP
C22orf29	ENSG00000215012	Multiple
PPP2R1B	ENSG00000137713	HITS-CLIP
ABL2	ENSG00000143322	Multiple
ZHX1	ENSG00000165156	PAR-CLIP
TET2	ENSG00000168769	HITS-CLIP
SERPINE1	ENSG00000106366	Multiple

SCARB2	ENSG00000138760	PAR-CLIP
CDK16	ENSG00000102225	Multiple
PUS1	ENSG00000177192	PAR-CLIP
HELLS	ENSG00000119969	HITS-CLIP
GCNT1	ENSG00000187210	Multiple
ZNF654	ENSG00000175105	HITS-CLIP
SNX17	ENSG00000115234	HITS-CLIP
PCDH9	ENSG00000184226	PAR-CLIP
IBA57	ENSG00000181873	PAR-CLIP
ATP5G2	ENSG00000135390	Multiple
PARD6B	ENSG00000124171	Multiple
PDHB	ENSG00000168291	PAR-CLIP
SLC25A33	ENSG00000171612	PAR-CLIP
GPR107	ENSG00000148358	Multiple
MAPKAPK2	ENSG00000162889	HITS-CLIP
SIM2	ENSG00000159263	PAR-CLIP
BRWD1	ENSG00000185658	Multiple
LETM1	ENSG00000168924	HITS-CLIP
SOX12	ENSG00000177732	HITS-CLIP
LYPLA1	ENSG00000120992	HITS-CLIP
SLC1A5	ENSG00000105281	Multiple
FBXO48	ENSG00000204923	PAR-CLIP
TECR	ENSG00000099797	PAR-CLIP
RAB14	ENSG00000119396	HITS-CLIP
ORC5	ENSG00000164815	HITS-CLIP
TMEM126A	ENSG00000171202	PAR-CLIP
TMEM200A	ENSG00000164484	HITS-CLIP
MED22	ENSG00000148297	Multiple
PRRC2A	ENSG00000204469	HITS-CLIP
MBOAT2	ENSG00000143797	HITS-CLIP
XKR8	ENSG00000158156	PAR-CLIP
NUP37	ENSG00000075188	PAR-CLIP
ELOVL4	ENSG00000118402	PAR-CLIP
DYNLRB1	ENSG00000125971	HITS-CLIP
ARMCX2	ENSG00000184867	HITS-CLIP
CMPK1	ENSG00000162368	PAR-CLIP
MTDH	ENSG00000147649	HITS-CLIP
TNRC6C	ENSG00000078687	HITS-CLIP
SYVN1	ENSG00000162298	HITS-CLIP
MYLK	ENSG00000065534	HITS-CLIP
INTS5	ENSG00000185085	HITS-CLIP
SUV39H1	ENSG00000101945	HITS-CLIP
ULBP1	ENSG00000111981	HITS-CLIP
RTN3	ENSG00000133318	HITS-CLIP

TMBIM6	ENSG00000139644	Multiple
NNT	ENSG00000112992	Multiple
EIF4G2	ENSG00000110321	HITS-CLIP
CLOCK	ENSG00000134852	HITS-CLIP
ZNF703	ENSG00000183779	PAR-CLIP
KBTBD7	ENSG00000120696	PAR-CLIP
ZMYND8	ENSG00000101040	HITS-CLIP
LSM4	ENSG00000130520	Multiple
KIF5B	ENSG00000170759	HITS-CLIP
C8orf88	ENSG00000253250	PAR-CLIP
MYO1B	ENSG00000128641	HITS-CLIP
PRRX1	ENSG00000116132	HITS-CLIP
SIN3A	ENSG00000169375	PAR-CLIP
USP34	ENSG00000115464	Multiple
EFHD1	ENSG00000115468	HITS-CLIP
NPTX1	ENSG00000171246	HITS-CLIP
ATR	ENSG00000175054	HITS-CLIP
MAP1B	ENSG00000131711	HITS-CLIP
DHX33	ENSG00000005100	Multiple
NUDT19	ENSG00000213965	HITS-CLIP
CDC25A	ENSG00000164045	HITS-CLIP
ANAPC16	ENSG00000166295	PAR-CLIP
TBR1	ENSG00000136535	PAR-CLIP
RPL8	ENSG00000161016	HITS-CLIP
ELF1	ENSG00000120690	Multiple
KMT2B	ENSG00000272333	HITS-CLIP
HIST1H4E	ENSG00000276966	Multiple
PIGW	ENSG00000277161	PAR-CLIP
HIST1H4B	ENSG00000278705	Multiple
ZNF776	ENSG00000152443	PAR-CLIP
PIP4K2B	ENSG00000276293	Multiple
NR4A1	ENSG00000123358	HITS-CLIP
PALM2-AKAP2	ENSG00000157654	Multiple
RASL10B	ENSG00000270885	HITS-CLIP
PSG7	ENSG00000221878	HITS-CLIP
TMEM189-UBE2V1	ENSG00000124208	HITS-CLIP
HIST1H4D	ENSG00000277157	Multiple
HIST1H2AD	ENSG00000196866	Multiple
MUC16	ENSG00000181143	HITS-CLIP
BRD4	ENSG00000141867	PAR-CLIP
RASSF5	ENSG00000266094	HITS-CLIP

Table S5. Upregualted DEMs shared between benign PNs, AIS/MIA, and invasive adenocarcinomas.

Categories	Total	Elements
Benign vs AIS/MIA<br>Benign vs Invasive<br>AIS/MIA vs Invasive	2	Let-7b-3p, miR-125b-5p
Benign vs AIS/MIA<br>Benign vs Invasive	6	miR-10b-5p, miR-92b-3p, miR-10a-5p, miR-328-3p, miR-1228-5p, miR-99a-5p
AIS/MIA vs Invasive<br>Benign vs Invasive	1	miR-100-5p
Benign vs Invasive	11	miR-125a-5p, miR139-5p, miR-27b-3p, miR-193a-5p, miR-99b-5p, miR-122-5p, let7d-3p, miR-483-5p, miR-150-5p, miR-3168, miR-24-3p
Benign vs AIS/MIA	3	miR-30d-5p, miR-625-3p, miR-423-3p
AIS/MIA vs Invasive	3	miR-126-5p, miR-30c-5p, miR-152-3p

Table S6. Down-regualted DEMs shared between benign PNs, AIS/MIA, and invasive adenocarcinomas.

Categories	Total	Elements
Benign vs AIS/MIA<br>Benign vs Invasive	7	miR-30e-5p, miR-502-3p, miR-503-5p, miR-101-3p, miR-144-3p, let-7g-5p, miR-500a-3p
Benign vs Invasive<br>AIS/MIA vs Invasive	3	miR-7-5p, miR-532-5p, miR-361-3p
Benign vs Invasive	7	miR-16-5p, miR-186-5p, miR-399-3p, let-7i-5p, miR-19a-3p, miR-148b-3p, miR-363-3p
Benign vs AIS/MIA	4	miR-21-5p, miR-103a-3p, miR-144-5p, miR-107
AIS/MIA vs Invasive	6	miR-486-3p, miR-25-3p, miR-106-3p, miR-223-5p, miR-128-3p, let-7b-5p

Table S7. Quality control of small RNA sequencing.

SampleID	Cohort	Total number of reads	Number of mapped reads	Mapping rate (%)	miRNA Mapping rate (%)	antisense Mapping rate (%)	intergenic Mapping rate (%)	intron Mapping rate (%)	lincRNA Mapping rate (%)	protein_coding Mapping	rRNA Mapping rate (%)
Training_P01	Training	6420144	6210073	96.727939	65.012314	2.0837785	1.5915433	17.678359	0.1438228	2.4432966	0.0858739
Training_P02	Training	7030808	6755119	96.078843	64.192956	2.8275347	1.7637883	20.020713	0.1046466	2.9181993	0.1219075
Training_P03	Training	6388398	6110294	95.646733	60.463925	2.3611753	2.028724	22.534611	0.1511766	2.9894934	0.134969
Training_P04	Training	7968751	7717493	96.846959	49.200815	2.8234039	1.6111191	17.831581	0.0859476	2.5577315	0.1020236
Training_P05	Training	5738301	5491814	95.70453	62.679762	2.9450105	2.2837081	24.395582	0.1207676	3.411605	0.1059031
Training_P06	Training	8368425	8058830	96.300439	63.96921	2.9020238	1.725449	19.163055	0.0943458	2.6005895	0.1821625
Training_P07	Training	8580149	7999226	93.229453	47.175842	4.0311733	5.302138	38.126264	0.1297751	3.104765	0.09448
Training_P08	Training	7305660	6740400	92.262711	42.766912	4.0512198	5.5316153	39.486855	0.1253041	4.3110201	0.1194909
Training_P09	Training	8717943	8215254	94.233858	52.820398	3.4828179	4.3877889	32.214817	0.1141292	3.3010533	0.154132
Training_P10	Training	6641324	6453619	97.173681	74.478294	1.8669886	1.4142298	16.040271	0.1004682	2.3355397	0.0930021
Training_P11	Training	8622964	8033675	93.166051	40.549977	4.5837241	6.2496803	43.603482	0.1424182	3.0840078	0.0987946
Training_P12	Training	8062412	7618913	94.499177	44.503628	4.0110884	6.0069199	40.136723	0.1571834	3.1294075	0.1155283
Training_P13	Training	8173494	7975203	97.573975	73.057745	1.4425443	1.270789	14.931607	0.1054707	2.4946899	0.057497
Training_P14	Training	8334820	7989530	95.857259	59.341459	2.7660263	1.7094497	20.521395	0.085625	2.5983468	0.0786905
Training_P15	Training	8692719	8265328	95.083345	55.904669	3.1158534	4.3687679	30.933836	0.1132784	2.8040871	0.0951505
Training_P16	Training	10023971	9679886	96.567378	68.50353	2.6603688	2.8110455	20.500551	0.0877438	2.3409108	0.09106
Training_P17	Training	7985174	7792781	97.590622	74.767877	1.6009946	1.175357	14.527766	0.0843259	2.2744736	0.0579703
Training_P18	Training	8536967	8289869	97.105553	73.898287	2.1279267	1.2076427	15.303933	0.0940445	2.9343578	0.0807291
Training_P19	Training	6811535	6518277	95.694686	65.511473	2.3000685	1.7849809	21.464046	0.1007373	2.8107581	0.1949672
Training_P20	Training	6670790	6377753	95.607162	61.295091	2.0375018	1.7424319	20.695126	0.1187592	2.7734624	0.1127017
Training_P21	Training	9012390	8719139	96.746135	63.813778	3.5495707	1.4078684	17.158747	0.0775535	2.3228928	0.1241006
Training_P22	Training	9485040	9199585	96.990471	58.675352	2.2000703	1.5396129	17.888796	0.0927524	2.9080026	0.1200453
Training_P23	Training	9540743	9341317	97.909744	77.652402	1.7113683	0.9382938	11.971503	0.0789949	2.6043232	0.1020056
Training_P24	Training	9717269	9131628	93.973194	37.911957	4.1267559	6.2277614	42.164782	0.1472227	3.5547906	0.0654593
Training_P25	Training	9724480	9333147	95.975795	65.353234	2.5316532	1.8556656	21.239856	0.1000984	3.4969912	0.1689516
Training_P26	Training	9461001	8953965	94.640779	27.482542	6.1691887	7.9899352	51.695847	0.1556582	3.1445641	0.0607608
Training_P27	Training	8830031	7904391	89.517138	31.906011	5.0903711	6.8018902	51.063162	0.1747215	3.2658114	0.1262315
Training_P28	Training	9517554	9308675	97.805329	76.198908	4.4416937	1.086782	12.827261	0.0954916	2.218163	0.0697092
Training_P29	Training	8986452	8392802	93.393945	42.984822	3.7649663	5.1011331	37.801214	0.1230757	3.3226408	0.0584946

Training_P30	Training	15039641	13969091	92.881812	47.555525	3.6496636	4.4129428	36.302062	0.1423995	2.9772994	0.0921618
Training_P31	Training	8565209	7962167	92.959401	36.528525	3.9531031	3.333816	36.144695	0.1357911	3.1794747	0.1259665
Training_P32	Training	8443278	8001885	94.772256	59.292112	2.7054188	2.3527831	26.86427	0.1115487	2.713896	0.1187345
Training_P33	Training	8898123	8507612	95.61131	51.537631	4.2881579	4.1629661	29.4869	0.1103639	3.070561	0.1220025
Training_P34	Training	8259814	7697632	93.193769	52.148628	3.6096653	4.275094	34.633495	0.1447924	2.8920019	0.2507243
Training_P35	Training	8215197	7599264	92.502517	40.942682	4.5617883	5.7807572	42.739652	0.1460141	3.0906173	0.1020691
Training_P36	Training	10546827	9826142	93.166807	34.373664	4.663056	6.4966902	48.357219	0.1575525	3.3911936	0.0857559
Training_P37	Training	7607870	7193838	94.557846	59.010502	3.0037281	2.4853354	27.08632	0.1721311	2.757105	0.1267775
Training_P38	Training	6877268	6504394	94.578167	50.540686	3.4980481	2.9721908	31.185473	0.1452044	3.5443591	0.1284337
Training_P39	Training	7978539	7744101	97.061643	60.1709	2.3663818	1.8844666	20.781044	0.0935658	2.7621067	0.1157698
Training_P40	Training	8626596	7630493	88.453116	30.892751	5.1174806	5.4304224	52.045916	0.1611298	3.6091934	0.105334
Training_P41	Training	8807754	8191165	92.999475	35.399531	4.510264	6.0783295	44.664233	0.1498322	3.1569166	0.0593872
Training_P42	Training	8187251	7883123	96.285347	51.340867	2.8072631	2.1762949	23.527008	0.1157642	2.8456446	0.0827189
Training_P43	Training	11657123	10958427	94.006274	52.123158	3.3685233	4.0903863	31.752851	0.185857	2.9839106	0.0735872
Training_P44	Training	10441650	9397702	90.002078	25.643669	5.6913736	7.8663592	55.690508	0.1729829	3.1926333	0.0797784
Training_P45	Training	12609717	11940591	94.693569	45.116026	4.1579502	5.4252842	37.647123	0.1300166	3.7992082	0.0635521
Training_P46	Training	10296580	9636985	93.594038	45.179365	4.3349243	5.5300283	39.034127	0.1283847	2.8295978	0.0725884
Training_P47	Training	9776103	8678220	88.769728	26.290765	5.7816484	7.8388886	55.315503	0.1687356	3.0946141	0.0700278
Testing_P48	Testing	8798653	8391964	95.377827	40.812294	3.8227762	5.5469494	36.934989	0.1439969	3.1561583	0.1634302
Testing_P49	Testing	6090018	5830626	95.740702	37.654522	4.8061277	6.421506	41.863515	0.1419116	3.1096861	0.1031822
Testing_P50	Testing	7113184	6801123	95.612921	47.304861	3.4059057	5.0827194	34.677347	0.1342229	3.4248229	0.1200782
Testing_P51	Testing	6995655	6690716	95.641023	50.504046	3.143616	4.0662165	30.821709	0.1312254	3.5601147	0.1911878
Testing_P52	Testing	7671052	7415133	96.663834	49.314861	2.8719939	3.8601331	26.765629	0.1089214	3.7328584	0.1277702
Testing_P53	Testing	7912879	7636533	96.507643	53.631546	3.1050216	4.5060239	30.286375	0.1129505	2.8883362	0.0886659
Testing_P54	Testing	8147273	7820632	95.990794	63.12943	4.1369888	3.464362	24.948009	0.0897413	1.9136806	0.0321756
Testing_P55	Testing	8146181	7826305	96.073301	47.07567	4.0794368	4.5632773	30.611125	0.1060058	2.6849761	0.0559332
Testing_P56	Testing	7397034	6884627	93.072805	40.907176	4.1949719	5.3171653	39.870875	0.1354213	3.5128192	0.1155328
Testing_P57	Testing	8153490	7708717	94.544999	46.678645	3.4360884	4.9050705	35.637474	0.1227813	3.4338036	0.1906024
Testing_P58	Testing	6839206	6441841	94.189896	30.662469	4.7936685	6.3028255	49.465021	0.1535017	3.7193224	0.0967011
Testing_P59	Testing	7664048	7334378	95.698487	53.514191	2.7514694	3.5390322	27.202661	0.110799	3.6921763	0.1820604
Testing_P60	Testing	8697882	8457393	97.235086	48.195872	2.1000916	2.7980726	20.974537	0.0773367	2.6077914	0.1098309
Testing_P61	Testing	9882099	9420815	95.332125	58.600051	2.5842969	3.8797599	28.063007	0.1040807	3.0382262	0.2735468

Testing_P62	Testing	6453830	6189655	95.906694	50.763007	3.4554861	4.6935572	32.914839	0.1163258	3.1482395	0.4345207
Testing_P63	Testing	6071162	5692474	93.762512	40.623711	4.4076471	5.719341	42.582856	0.1361447	3.1870223	0.1327572
Testing_P64	Testing	7207365	6548304	90.855729	53.263813	3.1282986	3.0923885	30.378889	0.117379	3.5576197	0.1357476
Testing_P65	Testing	7728240	7149117	92.506405	66.18604	2.8675056	2.106442	22.182516	0.0953777	3.2691543	0.1650623
Testing_P66	Testing	10335349	9718440	94.031077	64.836858	2.1786281	2.3034664	22.361449	0.0871428	4.016484	0.1640301
Testing_P67	Testing	8842456	8025820	90.764602	48.307326	2.9329277	2.8245338	30.826719	0.1108599	3.5843793	0.1286598
Testing_P68	Testing	9267396	8456051	91.245168	52.813731	2.8428696	2.6458568	28.450171	0.1319292	3.9670202	0.1861231
Testing_P69	Testing	5093530	4722612	92.71786	49.814502	2.5966986	2.3777096	24.924809	0.1088628	3.457557	0.0725058
Testing_P70	Testing	6837055	6181701	90.414674	58.689794	2.8401244	2.2817344	26.171842	0.1144682	3.2398459	0.4111759
Testing_P71	Testing	6088264	5608943	92.127132	51.372831	3.0550438	2.7017746	28.166287	0.1365052	3.8230183	0.186999
Testing_P72	Testing	7522844	6862122	91.217125	54.212358	2.9711485	2.7927659	29.600887	0.1249893	3.5260701	0.0781342
Testing_P73	Testing	6108211	5645778	92.429322	54.833278	2.3707184	2.252887	24.612551	0.1509801	3.8075606	0.1270802
Testing_P74	Testing	5579557	5153862	92.370452	55.26582	3.0230372	3.2176259	30.479939	0.1293706	3.6387147	0.149713
Testing_P75	Testing	5725124	5384489	94.050173	52.880636	2.543618	2.6856959	25.167198	0.1538215	3.5095299	0.1554744
Testing_P76	Testing	5464800	4902942	89.718599	50.100464	3.4788568	3.0789677	31.778308	0.1131504	3.7703356	0.1304761
Testing_P77	Testing	5953024	5290497	88.870749	59.337926	2.8680607	2.2738317	27.316432	0.1316385	3.3165992	0.2445233
Testing_P78	Testing	7977749	7557868	94.736849	67.293915	2.2059859	1.8947671	20.276419	0.078662	3.2441865	0.140604
Testing_P79	Testing	8188578	7821221	95.513788	72.776438	1.5802285	1.3423607	15.144132	0.0867362	3.425843	0.1197916
Testing_P80	Testing	5713940	5186723	90.773144	50.773934	3.6543241	3.2791418	31.989505	0.1190524	3.1317221	0.2225908
Testing_P81	Testing	5168901	4387355	84.879842	45.75506	3.7811772	3.2921202	36.948572	0.1460086	3.8450046	0.2219257
Testing_P82	Testing	5561755	5280336	94.940104	40.575467	6.1206837	5.4475511	39.878618	0.130118	2.685492	0.2003471
Testing_P83	Testing	7540631	7096751	94.11349	54.593979	2.8465679	2.7261912	26.129816	0.1038362	4.9407433	0.1542419
Testing_P84	Testing	5721595	5216472	91.17164	50.115698	3.5680277	3.4611324	32.239644	0.1126208	3.682933	0.2723552
Testing_P85	Testing	6087467	5492993	90.23446	44.745667	3.4382895	3.5460631	34.513734	0.1331059	3.6286031	0.1739337
Testing_P86	Testing	7315514	7028738	96.079893	65.986238	1.4481476	1.3810018	14.288753	0.1112243	3.7268023	0.1221675
Testing_P87	Testing	11246754	10094139	89.751576	62.356387	2.3287771	1.9493887	23.471036	0.0964421	4.414646	0.2749137
Testing_P88	Testing	10416017	9961098	95.632505	50.394497	3.3892549	4.0312022	32.323706	0.1188231	4.2458112	0.1168178
Testing_P89	Testing	8236835	7948590	96.500537	55.322771	2.7539109	3.4672313	25.304186	0.1013513	3.0241894	0.1310379
Testing_P90	Testing	7917555	7745275	97.824076	49.731828	3.1811653	4.453399	31.017595	0.1112054	3.2454088	0.1107966
Testing_P91	Testing	6631221	6351325	95.779118	42.649361	3.5324781	5.2917147	34.718378	0.1190959	3.6113003	0.1171094
Testing_P92	Testing	5828762	5434153	93.229969	37.105704	4.1778023	5.883824	41.883455	0.1417348	3.9539511	0.1874472
Testing_P93	Testing	6222768	5829665	93.682827	48.08434	3.4364868	4.5273442	34.401188	0.1230631	3.4495621	0.1441421

Testing_P94	Testing	8361269	7931636	94.861629	47.586095	3.2820593	3.9225829	33.91516	0.1134591	3.3948408	0.0963357
Testing_P95	Testing	7572777	7152678	94.452511	48.32135	3.1266751	4.354439	32.236513	0.1348155	3.4128464	0.1199551
Testing_P96	Testing	5702259	5420285	95.055048	28.988302	5.0835396	7.3789109	49.590769	0.1616797	3.3223791	0.0789411
Testing_P97	Testing	6610880	6208939	93.920008	32.121629	4.730105	6.9114063	47.856453	0.1668374	3.1744299	0.1226135
Testing_P98	Testing	6601956	6202312	93.946582	29.546358	5.522532	7.4201846	50.495864	0.2000517	2.923602	0.0666579
Testing_P99	Testing	6378678	5938977	93.106706	31.878481	4.7458089	6.1098906	48.334604	0.1827868	3.5384152	0.1377145
Testing_P100	Testing	6906790	6601317	95.577207	48.591972	2.7283722	3.5130263	26.581968	0.1049589	3.2326201	0.1542697
Testing_P101	Testing	12112806	11040357	91.146156	52.948644	2.555097	2.1565335	24.991275	0.1112789	4.6550216	0.4254814
Testing_P102	Testing	17877526	16345415	91.429961	57.885558	2.9464328	1.9318017	23.921944	0.1321737	4.0641912	0.3041719
Testing_P103	Testing	9700641	8362116	86.201685	51.646968	3.6236382	2.7513969	32.173448	0.1202865	4.0092025	0.1684163
Testing_P104	Testing	11180702	9938137	88.886521	40.701934	3.8412431	3.9040315	38.81798	0.162247	3.8225935	0.1479872
Testing_P105	Testing	13826070	12902715	93.321638	57.585715	2.3800546	1.9469778	22.910791	0.0967477	4.7231326	0.2696861
Testing_P106	Testing	14142420	13126680	92.817778	55.315315	3.2480477	2.3760616	27.092068	0.1062931	4.2354756	0.3015284
Testing_P107	Testing	13853107	13372137	96.528071	26.393347	1.6428283	1.3065376	14.684302	0.0745194	2.0001048	0.0283463
Testing_P108	Testing	12410136	11915628	96.015289	25.278224	2.2364271	1.3714342	16.013164	0.0973903	2.3386304	0.0577169
Testing_P109	Testing	13862046	12715028	91.725478	62.216553	2.5854904	1.8185096	23.150763	0.0946649	3.4905809	0.2130104
